# A Crystalline Bismuth(II) Radical Anion: Synthesis, Characterization, and Reactivity

**DOI:** 10.1002/anie.202515545

**Published:** 2025-10-12

**Authors:** Sotirios Pavlidis, Christian Teutloff, Ana Guilherme Buzanich, Konstantin B. Krause, Franziska Emmerling, Robert Bittl, Josh Abbenseth

**Affiliations:** ^1^ Institut für Chemie Humboldt‐Universität zu Berlin Brook‐Taylor‐Str. 2 12489 Berlin Germany; ^2^ Fachbereich Physik Freie Universität Berlin 14195 Berlin Germany; ^3^ Department of Materials Chemistry Federal Institute for Materials Research and Testing Richard‐Willstätter‐Str. 11 Berlin 12489 Germany; ^4^ Department of Chemistry University of Manchester Oxford Road Manchester M13 9PL U.K

**Keywords:** Bismuth, EPR spectroscopy, EXAFS, Radicals, XANES

## Abstract

We report the synthesis of a planarized tris‐amidobismuthane supported by a rigid, bulky NNN pincer ligand, which enforces a T‐shaped geometry at the bismuth center. The Bi(NNN) complex features a low‐lying LUMO with distinct Bi(6p) orbital character as shown by DFT calculations. Cyclic voltammetry reveals a fully reversible one‐electron reduction at *E*_1/2_ = –1.85 V versus Fc^0/+^ in THF. Chemical reduction with KC_8_ in the presence of 4,7,13,16,21,24‐hexaoxa‐1,10‐diazabicyclo[8.8.8]hexacosane (222‐crypt) enables the isolation of an unprecedented Bi(II) radical anion in high isolated yields. Multi‐frequency EPR, X‐ray absorption spectroscopy and SQUID magnetometry complemented by theoretical calculations confirm localization of the unpaired electron on the bismuth center. Preliminary reactivity studies display radical reactivity as shown by single‐electron transfer chemistry and radical coupling reactions.

## Graphical abstract

A T‐shaped Bi(III) trisamide featuring a sterically demanding NNN pincer ligand was prepared. The planarization of the Bi center furnishes a lowest unoccupied molecular orbital with distinct Bi(6p) orbital character which unlocks clean one‐electron reduction to afford a crystalline Bi(II) radical anion. Comprehensive spectroscopic analysis reveals the localization of the unpaired electron at the bismuth center further confirmed by reactivity studies.

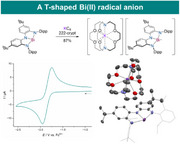

The synthesis and characterization of open‐shell main group compounds is a highly challenging task due to their inherent high reactivity and tendency to oligomerize.^[^ ^1^ ^]^ While numerous isolable radical species have been reported for lighter p‐block elements, the heavier analogues remain severely underexplored.^[^ ^2^, ^3^ ^]^ Bismuth, as the heaviest isolable pnictogen, represents an especially compelling case as recent studies have shown that Bi compounds can facilitate redox catalysis via both one‐ and two‐electron pathways.^[^ ^4^, ^5^, ^6^, ^7^, ^8^ ^]^ While Bi(II) radicals have been postulated as reactive intermediates, e.g., formed by (irradiation induced) Bi(III)–X bond homolysis, the number of isolated examples remains limited to neutral (Figure 1a–c) and cationic Bi radicals (Figure 1d–f).^[^ ^9^, ^10^, ^11^, ^12^, ^13^, ^14^, ^15^, ^16^, ^17^, ^18^, ^19^, ^20^, ^21^, ^22^, ^23^, ^24^, ^25^, ^26^, ^27^, ^28^, ^29^ ^]^ Both compound classes are usually stabilized upon utilization of sterically demanding and electron donating substituents to prevent undesired follow‐up reactivity such as dimerization. Their comprehensive characterization poses a significant challenge, as the large hyperfine interactions of the ^209^Bi isotope (*I* = –9/2; 100% natural abundance), which typically lie in the GHz range, necessitate multifrequency EPR spectroscopy at cryogenic temperatures, and in some cases have prevented complete analysis. Moreover, the accurate prediction of Bi hyperfine interaction coupling constants by theory remains elusive due to significant relativistic effects.^[^ ^15^, ^30^ ^]^ In stark contrast to the small number of neutral and cationic examples, no structurally characterized anionic Bi(II) radical has been reported to date, and the general chemistry of isolable Bi radicals as a whole remains largely undeveloped.

**Figure 1:**
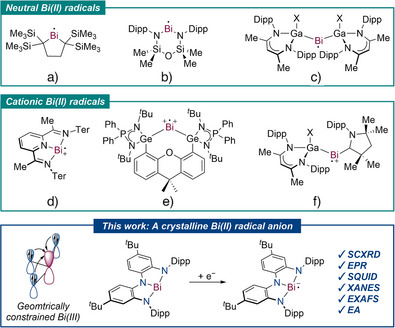
Reported neutral a)–c), top) and cationic d)–f), middle) Bi(II) radicals; Synthesis of a crystalline Bi(II) radical anion (bottom); Dipp = 2,6‐di‐*iso*‐propylphenyl; Ter = terphenyl, H; X = Cl, I.

Accessing such an anionic Bi(II) species presents both electronic and geometric challenges. In typical *C*_3v_‐symmetric BiR_3_ compounds, the lowest unoccupied molecular orbitals (LUMOs) correspond to a degenerate set of Bi–R σ*‐antibonding orbitals, rendering one‐electron reduction unfeasible due to facile bond cleavage. To stabilize an anionic Bi(II) radical, a bespoke Bi(III) starting material must feature a low‐lying acceptor orbital localized at Bi which is achievable via geometric distortion. A perturbation of tetrahedral PnR_3_ (Pn = pnictogen) species to bent (*C_S_‐*symmetric) structures and finally T‐shaped (*C*_2v_‐symmetric) geometries furnishes a low‐lying empty p‐orbital located at the pnictogen center which represents an intriguing design concept to access unprecedented radical species. VSEPR non‐compliant pnictogen pincer compounds have recently been shown to feature unique reactivity profiles, accessible radical anionic states and even allow for catalysis in selected cases.^[^ ^31^, ^32^, ^33^, ^34^, ^35^, ^36^, ^37^, ^38^, ^39^, ^40^, ^41^, ^42^ ^]^

However, the number of rigorously T‐shaped pnictogen species remains small, and their reactivity is not well understood. In this regard, T‐shaped phosphines display vast potential in small molecule activation reactions as well as unique bonding properties to transition metal complexes. Rigid NNN pincer ligand scaffolds are particularly efficient in this context.^[^ ^31^, ^36^, ^39^, ^40^, ^43^, ^44^, ^45^, ^46^ ^]^ For T‐shaped Bi(III), its empty 6p‐orbital can be coupled with redox‐active NNN pincer ligands which results in ambiphilic reactivity patterns as exemplified by adduct formation with nucleophiles, oxygen activation, coordination of transition metals or polymerization catalysis as recently demonstrated by the groups of Chitnis, Hwang, and our research group.^[^ ^40^, ^42^, ^43^, ^44^, ^47^, ^48^, ^49^, ^50^, ^51^ ^]^ Yet, whether this geometry can be leveraged to access isolable Bi(II) radical anions has not been explored. Here, we report the synthesis and isolation of a crystalline Bi(II) radical anion, made possible by one‐electron reduction of a geometrically constrained tris‐amidobismuthane featuring a bulky NNN pincer ligand.

Reaction of the sterically demanding NNN protioligand **1** ^[^ ^52^ ^]^ recently reported by Su, Wang, and coworkers with Bi(NMe_2_)_3_ in toluene at room temperature yields the T‐shaped tris‐amidobismuthane [Bi(NNN)] (**2**) as dark blue crystals in 68% isolated yield (Figure 2). ^1^H and ^13^C{^1^H} NMR spectroscopic results are in line with the formation of a *C*_2v_ symmetric species on the NMR timescale, which is further confirmed by single‐crystal X‐ray diffraction (scXRD, Figure 2) revealing a planarized T‐shaped Bi(NNN) complex. The Bi–N, C–N, and C–C bond lengths within the pincer scaffold compare well with prior reported examples of planarized Bi(NNN) species indicating extensive electronic coupling between the redox‐active ligand scaffold and the empty Bi(6p) orbital (see Supporting Information).^[^ ^43^, ^48^, ^51^ ^]^ The frontier molecular orbitals are in line with this description showing the LUMO to be an antibonding combination of a Bi(6p) orbital and the π‐manifold of the NNN pincer ligand (Figure 2). The highest occupied molecular orbital (HOMO) represents its bonding combination while the HOMO–1 and HOMO–2 are ligand centered (see Supporting Information).

**Figure 2:**
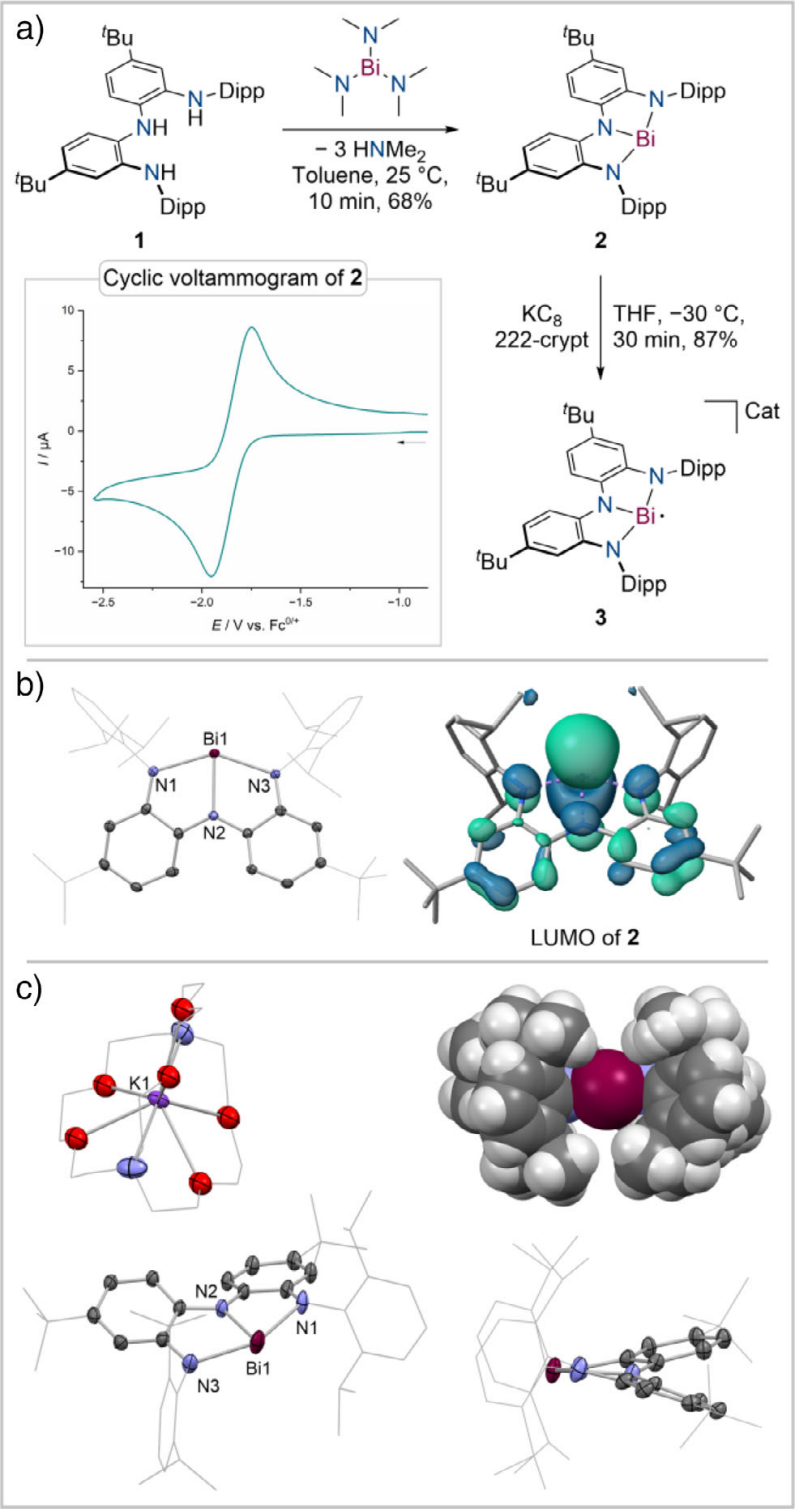
a): Synthesis of **2** and **3**, Cat = K@222‐crypt⋅THF; Inset: Cyclic voltammogram of **2** in THF, 200 mV s^−1^, 25 °C, 1 mM, 0.1 M [NBu_4_][PF_6_] as electrolyte (top); b): Bond lengths of **2** in Å, angles in °: Bi1–N1 2.2722(19); Bi1–N2 2.2053(18); Bi1–N3 2.2736(19); N1–Bi1–N3 143.79(7) (left), computed LUMO of **2** (right); c): Bond lengths of **3** in Å, angles in °: Bi1–N1 2.315(5); Bi1–N2 2.225(4); Bi1–N3 2.303(5); N1–Bi1–N3 143.4(2); Space‐filling representation of **3**, with all atoms scaled to their van der Waals radii. Molecular structures of **2** and **3** in the solid state derived by scXRD are shown at 50% ellipsoid probability; solvent molecules and hydrogen atoms were omitted for clarity.

The possibility of reducing **2** by one electron to yield a Bi(II) radical anion was initially evaluated by cyclic voltammetry (CV) measurements in THF. We have recently reported a PBiP pincer complex featuring a T‐shaped Bi(NNN) fragment as the central donor moiety. CV revealed a quasi‐reversible reduction at *E*_1/2_ = –1.98 V versus Fc^0/+^ suggesting that insufficient steric shielding of the generated Bi(II) center leads to rapid follow‐up reactivity.^[^ ^51^ ^]^ In contrast, a fully reversible one‐electron reduction was detected for **2** at *E*_1/2_ = –1.85 V versus Fc^0/+^ in THF (Figure 2). Chemical reduction of **2** with one equivalent of KC_8_ in THF at –30 °C in the presence of the sequestering agent 222‐crypt results in the formation of dark turquoise crystals from a THF/hexane layer after being stored at –30 °C for 3 days. scXRD as well as combustion analysis confirmed the successful isolation of the Bi(II) radical anion **3** as a [K@222‐crypt]^+^ salt with one equivalent of co‐crystallized THF in 87% isolated yield (Figure 2). The ^1^H NMR spectrum of a suspension of **3** in THF‐d_8_ features signals for [K@222‐crypt]^+^ as well as one equivalent of co‐crystallized THF‐d_0_ while no indicative signals for the Bi(NNN)^–^ moiety could be assigned. The overall planarity of the Bi center is preserved in **3**, accompanied by an elongation of the Bi–N bonds compared to neutral **2**, while the ligand backbone exhibits an almost identical twist angle to that in **2** to accommodate the large Bi center. Interestingly, the pincer ligand's C–N bond lengths elongate and aromatic C–C bonds become more equidistant reflecting a substantial decrease in electron donation from the redox‐active pincer ligand to the Bi center in **3** upon population of the vacant Bi(6p)‐orbital of **2** with an excess electron (Figure 2, see Supporting Information).

Superconducting quantum interference device (SQUID) magnetometry confirms the presence of an *S* = ½ compound yielding an effective magnetic moment of *χ*_P_ *T* = 0.335 cm^3^ mol^−1^ K (*µ*_eff_ = 1.64 *µ*_B_) at 300 K close to the expected value for a *g* = 2 system (*χ*_P_ *T* = 0.376 cm^3^ mol^−1^ K; *µ*_eff_ = 1.73 *µ*_B_). A *g*‐value of *g*_exp_ = 1.71 is obtained from fitting procedures, in line with the presence of a less than half filled p‐shell at the Bi(II) center. Significant temperature‐independent paramagnetism^[^ ^53^, ^54^, ^55^, ^56^ ^]^ is observed with *χ_TIP_* = 1.76 10^−4^ cm^3^ mol^−1^ stemming from the large degree of spin‐orbit coupling of Bi resulting in linear decrease of the effective magnetic moment at lower temperatures (15 K: *χ*_P_ *T* = 0.274 cm^3^ mol^−1^ K; *µ*_eff_ = 1.48 *µ*_B_).^[^ ^57^ ^]^ These values compare well with the prior reported neutral and cationic Bi(II) radicals that also consistently exhibit *g*‐values below two and significant temperature independent paramagnetism.^[^ ^9^, ^10^, ^11^, ^12^, ^13^, ^14^, ^15^ ^]^

**2** and **3** were further characterized by X‐ray absorption near edge structure (XANES) spectroscopy at the Bi L_1_‐ and L_3_‐edge and extended X‐ray absorption fine structure (EXAFS) spectroscopy at the Bi L_3_‐edge. In contrast to isolable Bi(I) and Bi(III) compounds, X‐ray absorption spectroscopic characterization of molecular Bi(II) species has not been reported so far.^[^ ^58^ ^]^ **2** features edge energies of 16386.6 eV (L_1_‐edge) and 13417.8 eV (L_3_‐edge). The white line energies are determined to be 16394.9 eV (L_1_‐edge) and 13438.9 eV (L_3_‐edge). Interestingly, **3** exhibits quite similar edge (L_1_: 16386.6 eV; L_3_: 13419.0 eV) as well as white line energies (L_1_: 16394.9 eV; L_3_: 13437.8 eV) which contrasts with previously reported investigations of Bi(I) and Bi(III) complexes in which energy shifts of the edge positions between 0.8–2.3 eV have been observed (Figure 3).^[^ ^58^ ^]^ Compared to the reference BiPh_3_ (L_1_‐edge: 16388.5 eV; L_1_‐whiteline: 16397.5 eV), a shift to lower energies is being observed for **2** and **3** in line with previous results on planarized phosphorus and Bi(NNN) species featuring low‐lying LUMOs with high Pn(p) character.^[^ ^51^, ^59^ ^]^ This provides further evidence for the substantial transfer of electron density from the redox‐active pincer ligand toward the T‐shaped Bi center in **2** that was also found to unlock metal coordination in previous studies on related Bi(NNN) complexes.^[^ ^43^, ^51^ ^]^ Computations by Chitnis, Andrada, Salvador, and us showed that planarized Bi(III) NNN pincer compounds feature a strongly polarized Bi–N π‐bond and overall pronounced delocalization of electron density from the ligand toward the Bi(6p) orbital as further evidenced by a formal population of the empty Bi(6p) orbital by approximately one electron as derived by natural bonding orbital calculations.^[^ ^43^, ^51^, ^60^ ^]^ Reduction of **2** to **3** overwrites this donation resulting in similar XANES profiles for both compounds. The enlargement of the Bi–N bonds upon reduction of **2** to **3** leads to a shift in the scattering response in the extended X‐ray absorption fine structure (EXAFS) spectra at the Bi L_3_‐edge due to increased distances, in full agreement with our scXRD results (see Supporting Information).

**Figure 3:**
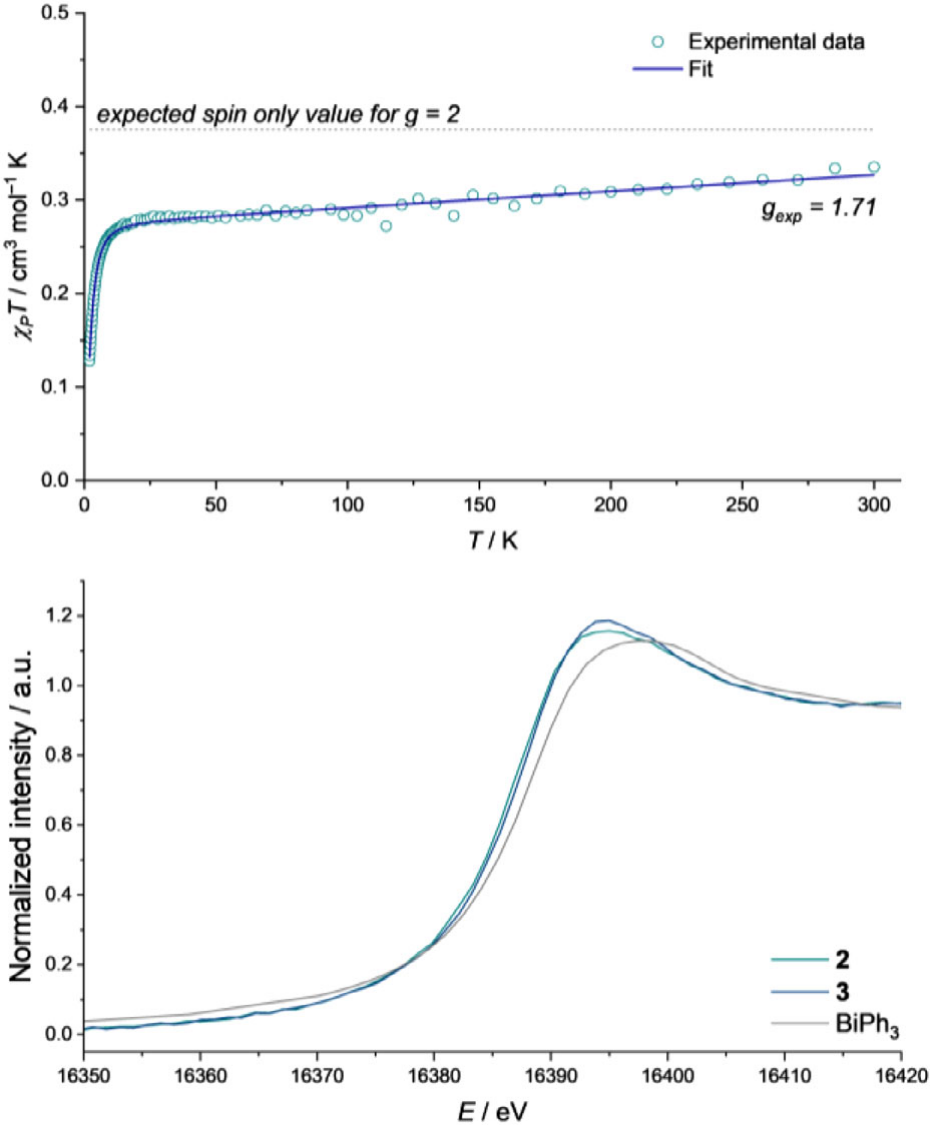
Magnetic susceptibility data of **3**, experimental data in turquoise circles, fitted data as a blue line (top); XANES spectra of **2**, **3**, and BiPh_3_ at the Bi L_1_‐edge.

The presence of a bismuth‐centered radical was unequivocally confirmed by EPR spectroscopy. Attempts to detect **3** in a frozen 2‐methyltetrahydrofuran solution at 100 K yielded no observable signals. However, at 10 K, a complex and broad signal was detected, indicating the existence of a heavy‐element radical species exhibiting rapid spin relaxation at higher temperatures. Localization of the unpaired electron at the Bi(II) center produces hyperfine coupling constants in the GHz range, resulting in very broad and complex EPR spectra. To fully resolve the entire signal, which spans approximately 1500 mT, W‐band EPR spectroscopy (94 GHz) was required, as lower‐frequency techniques such as X‐band (9.5 GHz) and Q‐band (34 GHz) did not provide sufficient magnetic field coverage to capture the complete signal. Spectrum simulation proved challenging due to a combination of low *g*‐anisotropy, large hyperfine coupling constants, and line broadening, which collectively hindered satisfactory spectral reproduction. Nevertheless, simulation of the experimental W‐band spectrum revealed a rhombic *g*‐matrix, and a nearly axial ^209^Bi hyperfine coupling. Critically, resolution of the *g*_z_ component (*g*_z_ = 1.79) enabled extraction of a large bismuth hyperfine coupling constant of *A*_z_(^209^Bi) = 4.0 GHz. This enormous value is fully consistent with the presence of a Bi(II) radical species with spin density being mainly located in the Bi(6p) orbital (see Supporting Information for further details.)

Theoretical calculations further elucidated the electronic structure of **3**. The α‐SOMO represents the antibonding combination of the Bi(6p) orbital with the π‐system of the NNN pincer ligand scaffold which rationalizes the observed bond alteration upon one‐electron reduction of **2** toward **3** (see Supporting Information). The calculated spin densities indicate significant electron localization on the planarized Bi center with partial delocalization over the ligand scaffold, fully in line with our experimental results (Figure 4). A low *g* anisotropy is reproduced by theoretical calculations, in stark contrast to the priorly reported neutral and cationic Bi radicals **B**, **D**, and **E**, likely caused by partial delocalization of electron density toward the NNN pincer ligand.^[^ ^10^, ^14^, ^15^, ^28^ ^]^

**Figure 4:**
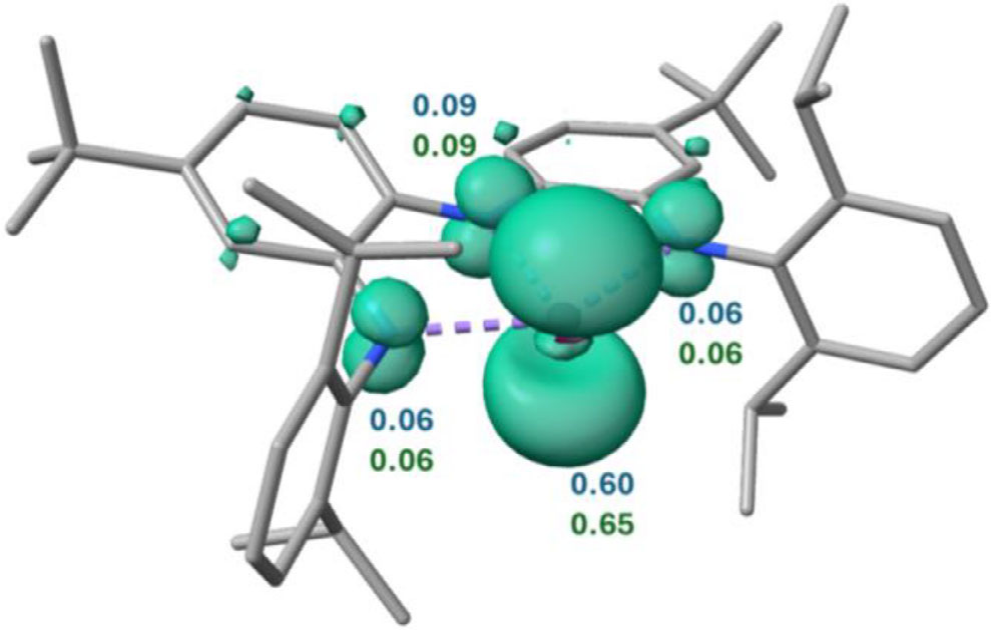
Spin density plot of **3** (selected Löwdin and Mulliken spin densities of the Bi center and the adjacent nitrogen atoms in blue and green, respectively); Computational method: D3BJ‐RIJCOSX‐PBE0/ZORA‐TZVPP//D3BJ‐RIJCOSX‐PBE0/def2‐SVP{Bi:def2‐TZVP}.

Due to pronounced relativistic effects, bismuth compounds often exhibit markedly different reactivities and bond strengths compared to lighter pnictogen congeners. The poor orbital overlap resulting from bismuth's large, diffuse, and essentially non‐hybridized frontier orbitals (6s, 6p) leads to weaker Bi–element bonds and distinct chemical behavior, making reactivity studies on Bi radical compounds particularly compelling.^[^ ^1^, ^4^, ^5^ ^]^ However, such species remain rare, and anionic derivatives are entirely unexplored. When **3** reacts with the stable 2,4,6‐tris‐*tert*‐butylphenoxyl radical (Ph*O**·**), clean regeneration of **2** occurs via single‐electron transfer (Figure 5). **2** and the corresponding potassium phenoxide anion were observed in a 1:1 ratio by ^1^H NMR spectroscopy. The latter crystallized from the reaction mixture upon layering with hexane at −30 °C and was further characterized by independent synthesis (see Supporting Information). Mixing **3** and Ph_3_C**·**resulted in no observable reaction, while reacting **3** with the slightly smaller (2,2,6,6‐Tetramethylpiperidin‐1‐yl)oxyl (TEMPO) radical results in formation of the diamagnetic complex anion [K@222‐crypt][Bi(TEMPO)(NNN)] (**4**, Figure 5) featuring a Bi–O bond. **4** can be isolated in 73% yield and displays extremely high reactivity in solution as well as in the solid state and could not be characterized via scXRD due to immediate decomposition when exposed to air. NMR spectroscopy in combination with elemental analysis, however; confirms the successful synthesis of **4**. In addition, reaction of **3** with the radical trapping reagent diphenyl diselenide yields the anionic Bi(III) complex [K@222‐crypt][Bi(SePh)(NNN)] (**5**, Figure 5) resulting from a Bi–Se coupling reaction in 80% isolated yield. scXRD reveals the presence of a four‐coordinate Bi compound in which the NNN pincer ligand adopts a markedly non‐planar geometry (see Supporting Information). DFT calculations demonstrate that Bi–Se bond formation lifts the pronounced coupling between the NNN pincer scaffold and the Bi center since no accessible acceptor orbital is present at the Bi center to allow for electron delocalization as shown by the calculated HOMO and LUMO (see Supporting Information). Consequently, the pincer ligand features almost equidistant aromatic C–C bonds. As observed for **4**, **5** features limited stability in solution and in the solid state and immediately decompose when exposed to trace amounts of water or oxygen.

**Figure 5:**
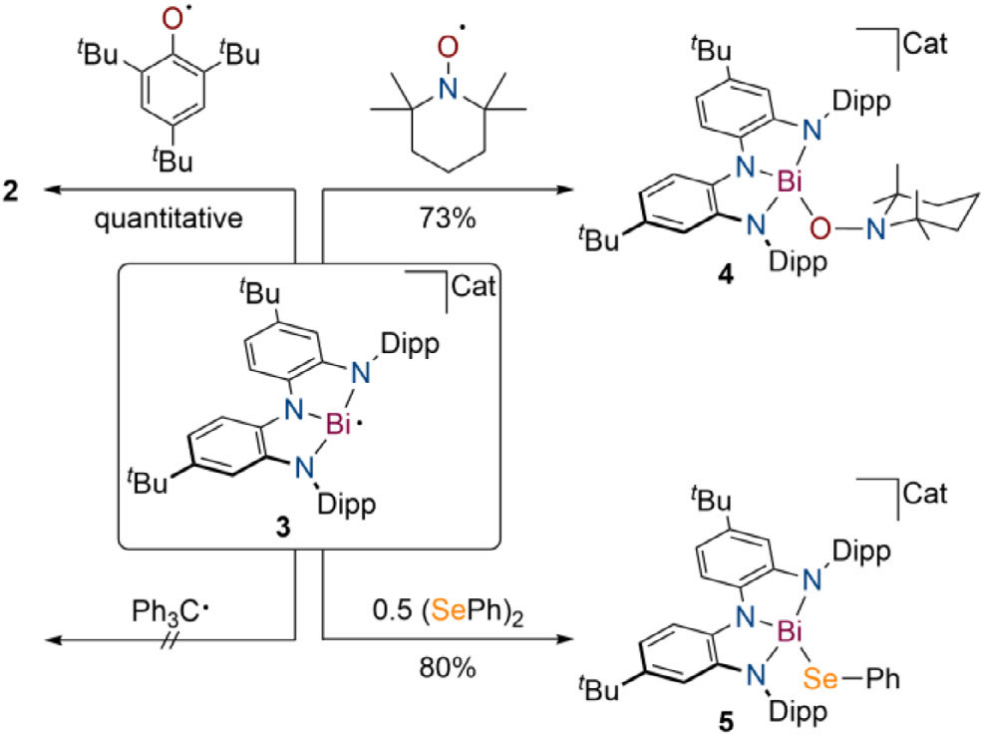
Reactivity of **3** toward Ph*O**·**, Ph_3_C**·**, TEMPO and diphenyl diselenide; Cat = [K@222‐crypt].

In conclusion, we report the isolation of a structurally defined anionic Bi(II) radical, filling a longstanding gap in the chemistry of bismuth‐based open‐shell species. Planarization of a Bi(III) center via a rigid, redox‐active NNN pincer ligand creates a low‐lying acceptor orbital, enabling a clean one‐electron reduction to the radical anion. A combination of SQUID magnetometry, multifrequency EPR, and XAS spectroscopy, supported by DFT calculations confirms the Bi‐centered radical character. Preliminary reactivity studies reveal typical radical‐type behavior, establishing this species as a rare and versatile platform for exploring the redox chemistry of heavy p‐block elements. Ongoing work in our laboratories aims to harness anionic Bi(II) radicals in synthetic and catalytic transformations.

## Supporting Information

Synthetic details; recorded NMR, IR, XAS and EPR spectra; SQUID and CV data; scXRD details; Computational details;. xyz coordinates of computed species. CCDC 2467330, 2467405 and 2467406 contain the supplementary crystallographic data for this paper. These data can be obtained free of charge from The Cambridge Crystallographic Data Centre via www.ccdc.cam.ac.uk/structures. The authors have cited additional references within the Supporting Information.^[^ ^61^, ^62^, ^63^, ^64^, ^65^, ^66^, ^67^, ^68^, ^69^, ^70^, ^71^, ^72^, ^73^, ^74^, ^75^, ^76^, ^77^, ^78^, ^79^, ^80^, ^81^, ^82^, ^83^, ^84^, ^85^, ^86^, ^87^, ^88^, ^89^, ^90^, ^91^, ^92^, ^93^, ^94^, ^95^, ^96^, ^97^ ^]^

## Conflict of Interests

The authors declare no conflict of interest.

## Supporting information



Supporting Information

## Data Availability

The data that support the findings of this study are available in the supplementary material of this article.
