# Supplementary material for: A Crystalline Bismuth(II) Radical Anion: Synthesis, Characterization, and Reactivity
Source: Angew Chem Int Ed Engl. 2025 Oct 12;64(49):e202515545. doi: 10.1002/anie.202515545 (PMC12668313; doi:10.1002/anie.202515545)
Supplement: Supplementary file 1 — Supporting Information [file ANIE-64-e202515545-s001.pdf]

Electronic Supporting Information

A Crystalline Bismuth(II) Radical Anion: Synthesis, Characterization and Reactivity

Sotirios Pavlidis,[a] Christian Teutloff,[b] Ana Guilherme Buzanich,[c] Konstantin B. Krause,[a] Franziska Emmerling,[c] Robert Bittl,[b] Josh Abbenseth[a,d]*

[a] Institut für Chemie, Humboldt-Universität zu Berlin, Brook-Taylor-Str. 2, 12489 Berlin, Germany

[b] Fachbereich Physik, Freie Universität Berlin, 14195 Berlin, Germany

[c] Department of Materials Chemistry, Federal Institute for Materials Research and Testing, Richard-Willstätter-Str. 11, 12489 Berlin, Germany

[d] Department of Chemistry, University of Manchester, Oxford Road, Manchester M13 9PL, U.K.

E-Mail: josh.abbenseth@manchester.ac.uk

Table of Contents

Materials and Methods S4

Synthetic procedures S6

Synthesis of 1 .... S6

Synthesis of 2 .... S7

Synthesis of 3 .... S8

One-electron oxidation of 3 by Ph*O S10

Synthesis of [K@222-crypt][Ph*O] S9

Unsuccessful Reaction of 3 with Gomberg's dimer S10

Synthesis of 4 .... S11

Synthesis of 5 .... S12

NMR and IR spectroscopy S13

NMR and IR spectroscopy of 1 .... S13

NMR and IR spectroscopy of 2 .... S14

NMR and IR spectroscopy of 3 .... S17

NMR spectroscopy of [K@222-crypt][Ph*O] S18

NMR spectroscopy of the one-electron oxidation of 3 by Ph*O S21

NMR spectroscopy of 3 with Gomberg's dimer S22

NMR and IR spectroscopy of 4 .... S23

NMR and IR spectroscopy of 5 .... S25

Single Crystal X-ray Diffraction S29

Crystallographic Details of 2 .... S29

Crystallographic Details of 3 .... S32

Crystallographic Details of [K@222-crypt][Ph*O] S36

Crystallographic Details of 5 .... S39

Cyclic voltammetry S47

EPR spectroscopy S48

SQUID magnetometry S52

X-ray absorption spectroscopy S56

Density functional theory calculations S60

Frontier molecular orbitals of 2 .... S61

Frontier molecular orbitals and spin densities of 3 .... S62

Frontier molecular orbitals of 4 .... S63
Structural comparison of computed structures S64
References S67

Materials and Methods

All experiments with air-sensitive compounds were carried out in a glovebox or in a fume hood employing Schlenk techniques under a dry Ar atmosphere. Traces of water and oxygen were removed via heating of glassware under vacuum prior to use.

All solvents except THF were dried and degassed by a MBraun solvent purification system. THF was dried over sodium, distilled and stored over molecular sieves. Deuterated solvents were degassed via three freeze-pump-thaw cycles and stored over molecular sieves.

All commercially available chemicals were used without purification unless otherwise noted. Boron nitride was dried at 120 °C for seven days under a dynamic vacuum. $BiCl_3$ was sublimed and grinded under an Ar atmosphere prior to use. $[NBu_4][PF_6]$ was dried at 120 °C for three days under a dynamic vacuum prior to use. 2,6-Diisopropylaniline was distilled and stored under inert atmosphere prior to use. (2,2,6,6-Tetramethylpiperidin-1-yl)oxyl (TEMPO) was sublimed at room temperature prior to use.

Tris-*tert*-butylphenoxyl radical (Ph*O),[1] KC_8,[2] $Bi(NMe_2)_3$[3], bis(2-bromo-4-*tert*-butylphenyl)amine (**I**)[4] and the Gomberg's dimer (1-(Diphenylmethylene)-4-trityl-2,5-cyclohexadiene)[5] were synthesized according to literature procedures.

NMR spectra were recorded on AVANCE II 300 MHz, Bruker Avance 400 MHz and Bruker Avance III 500 MHz NMR spectrometers. Chemical shifts are referenced to the signal of residual protonated solvent.

IR spectra were recorded on a Bruker ALPHA spectrometer with an ATR sampling unit.

Elemental analyses were performed with a HEKA Euro 3000EA elemental analyzer.

Cyclic voltammetry was performed in an Ar filled glovebox at room temperature with a PalmSens Emstat3+ Blue potentiostat using a glassy carbon working electrode, a Pt wire as counter electrode and an Ag wire as pseudo-reference electrode.

Magnetic measurements were performed with a QuantumDesign MPMS3 SQUID magnetometer. The samples were prepared in a glove box in VSM powder capsules which were sealed with a piece of Teflon tape. Both the capsules and the Teflon tape were dried in a Schlenk flask under vacuum at 110 °C for five days. A brass sample holder was used. The measurement was carried out in VSM mode from 2 K to 300 K in a magnetic field of 7 T after cooling down in a field of 7 T. A background correction was applied by subtracting the magnetic moments of an empty capsule sealed with a piece of Teflon tape using the same measurement sequence as for the sample. A diamagnetic correction was performed using Pascal's constants.[6] The simulation of the experimental magnetic data was performed with N. F. Chilton's PHI software (version 3.1.6)[7] using the following spin Hamiltonian:

$$\hat{H} = g * \mu_B * B_0 * \hat{S}$$

XAS measurements were performed at the BAMline located at BESSY-II storage ring operated by Helmholtz Centre Berlin (HZB).[8]

The incident energy was tuned by a double crystal monochromator in a Si(111) arrangement (delivering an intrinsic resolution of $\Delta E/E= 2x10^{-4}$). The measurements were carried out in transmission using three ionisation chambers to measure the signal before the sample (I_0), after the samples (I_1) and after a Bi metal reference foil (I_2). The energy range scanned comprised in total 1000 eV, starting at -200 eV below and ending at 800eV above the Bi L_1- and L_3-edges in a continuous mode. For XANES measurements we used equidistant 0.25 eV energy steps, and for EXAFS 0.5 eV. The samples were prepared and diluted with boron nitride in 2mm thick sample holders, to produce an absorption edge jump of 2. A total of 3 repetitions were collected for each sample. The acquired spectra were extracted, calibrated, and normalized using the IFFEFIT software package containing ATHENA and ARTEMIS software.[9] The Fourier Transformed EXAFS data were made in k-space (between 1.5-12 $Å^{-1}$)

EPR measurements at cryogenic temperature (10-20 K) were performed using an Elexsys E580 spectrometer (Bruker Biospin, Karlsruhe, Germany) equipped either with an ER 4118X-MD5 probehead (X-band) or a home-built probehead (Q-band), and an Elexsys E680 W-band (94 GHz) spectrometer equipped with a Teraflex EN600-1021H probe head (both Bruker). Samples were prepared in a glovebox under strictly anaerobic and water-free conditions. The crystalline material was dissolved in Me-THF, filtrated and filled in to heat-dried EPR tubes (Quartz EPR tubes for X-Band (3.0/4.0 mm i.d./o.d.), Q-band (2.0/2.9 mm i.d./o.d., QsIl, Germany) and for W-band (0.70 /0.87 mm i.d./o.d., VitroCom Inc, USA)). The Tubes were immediately frozen in liquid nitrogen, flame-sealed under He atmosphere at 600 mbar (X-/Q-band), or closed with Critoseal (W-band). Temperature control was achieved by a CF935 helium bath cryostat and an ITC503 temperature controller (both Oxford Instruments, UK). Simulation of the EPR spectra were performed with the MATLAB toolbox EasySpin version 6.0.6.

Synthetic procedures

Synthesis of 1

The NNN pincer ligand **1** was synthesized according to a protocol reported by Su, Wang and co-workers with minor modifications.[10]

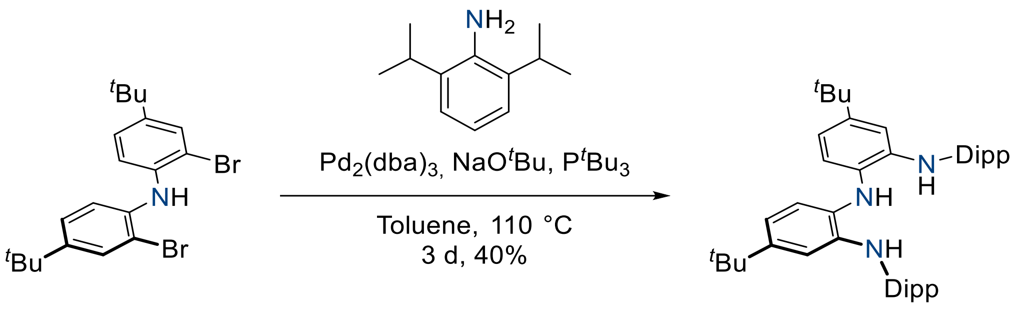

I **1**

I (2.00 g, 4.55 mmol, 1.00 eq.), tris(dibenzylideneacetone)dipalladium (210 mg, 0.229 mmol, 5 mol%), sodium *tert*-butoxide (1.31 g, 13.6 mmol, 3.00 eq.) and tri-*tert*-butylphosphine (93.0 mg, 455 µmol, 10 mol%) are added to a J-Young flask and suspended in toluene (80 mL). 2,6-Di-*iso*-propylaniline (2.57 mL, 13.6 mmol, 3.00 eq.) is added to the stirring suspension. The black reaction suspension is heated to 110 °C and stirred for three days in the dark. After cooling the reaction suspension to 25 °C, a degassed, saturated aqueous NH_4Cl solution (80 mL) is added and the mixture is stirred for 15 min, followed by decanting off the aqueous phase and subsequent washing with degassed water (2 x 50 mL) under inert atmosphere. The organic phase is dried over $MgSO_4$ and filtered. The solvent is removed with a rotary evaporator under reduced pressure. The crude product is purified via silica flash column chromatography DCM/hexane (1:3), followed by recrystallization from dry, degassed methanol under an inert atmosphere. Decanting the supernatant solution and removing residual solvent in vacuo yields **1** as white crystals (1.16 g, 1.84 mmol, 40%).

See

Figure S1 and **Figure S2** for NMR and IR spectra, respectively.

NMR: ($CDCl_3$, 25 °C) ^{1}H NMR (300 MHz) δ(ppm)= 7.35 – 7.22 (m, 6H, (Dipp)Ar*H*), 6.91 (d, *J* = 8.1 Hz, 2H, Ar*H*), 6.78 (dd, *J* = 8.1, *J* = 2.2 Hz, 2H, Ar*H*), 6.30 (d, *J* = 2.1 Hz, 2H, Ar*H*), 5.36 (s, 2H, N*H*), 5.09 (s, 1H, N*H*), 3.14 (hept, *J* = 6.9 Hz, 4H, C*H*(CH_3)$_2$), 1.19 (s, 18H, C(CH_3)$_3$), 1.12 (d, *J* = 6.9 Hz, 24H, (CH(CH_3)$_2$).

ATR-IR (solid): $\tilde{\nu}$ (cm^{-1}) = 3407.9 (w), 3358.7 (w), 3332.0 (w), 2958.3 (s), 1398.1 (s), 1256.4 (s) 798.6 (s).

Elem. Anal. found (calcd) for ($C_{44}H_{61}N_3$): C, 83.58 (83.62); H, 9.46 (9.73); N, 6.74 (6.65).

Synthesis of 2

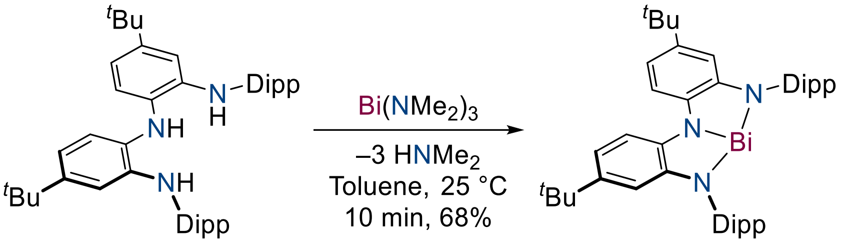

1 **2**

1 (300 mg, 475 μmol, 1.00 eq.) is dissolved in toluene (15 mL) and added to a Schlenk flask with $Bi(NMe_2)_3$ (162 mg, 475 μmol, 1.00 eq.) dissolved in toluene (5 mL) under an inert atmosphere. The dark blue reaction solution is stirred for 10 min at 25 °C, followed by removal of all volatiles under reduced pressure and extraction with hexane (2 x 5 mL). The concentrated filtrate is stored at −80 °C for three days. After filtration and removing of residual solvent in vacuo **2** is obtained as dark blue crystals (270 mg, 322 μmol, 68%).

See **Figure S3 - Figure S7** and **Figure S8** for NMR and IR spectra, respectively.

NMR: (C_6D_6, 25 °C) ^{1}H NMR (500 MHz) δ(ppm)= 8.48 (d, J = 9.1 Hz, 2H, ArH), 7.37 (d, J = 7.72 Hz, 4H, DippCH), 7.25 (t, J = 7.72 Hz, 2H, DippCH), 6.97 (d, J = 2.2 Hz, 2H, ArH), 6.71 (dd, J = 9.1, J = 2.3 Hz, 2H, ArH), 2.69 (hept, J = 6.9 Hz, 4H, CH(CH_3)$_2$), 1.32 (s, 18H, C(CH_3)$_3$), 1.12 (d, J = 6.9 Hz, 12H, CH(CH_3)$_2$), 1.09 (d, J = 7.0 Hz, 12H, (CH(CH_3)$_2$). ^{13}C{^{1}H} NMR (126 MHz) δ(ppm)= 156.19(ArC), 147.36(ArC), 146.87(Ar^{Dipp}C), 144.54(ArC), 139.62(Ar^{Dipp}C), 127.13(Ar^{Dipp}CH), 123.58(Ar^{Dipp}CH), 121.38(ArCH), 115.58(ArCH), 113.67(ArCH), 33.61(CH(CH_3)$_3$, 32.48(CH(CH$_3$)$_3$), 28.03(CH(CH_3)$_2$), 25.96(CH(CH$_3$)$_2$), 25.37(CH(CH$_3$)$_2$).

ATR-IR (solid): $\tilde{\nu}$ (cm^{-1}) = 2952.2 (s), 2863.9 (m), 1441.2 (s), 1283.1 (s), 1252.3 (s), 790.4 (s).

Elem. Anal. found (calcd) for ($C_{44}H_{58}BiN_3$): C, 63.26 (63.07); H, 7.10 (6.98); N, 4.89 (5.01).

Synthesis of 3

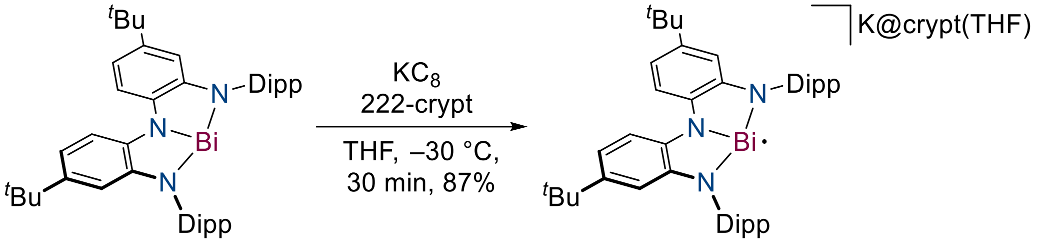

2 (36.7 mg, 43.8 µmol, 1.00 eq) and 222-crypt (16.5 mg, 43.8 µmol, 1.00 eq) are added to a scintillation vial and dissolved in THF (4 mL) along with a stirring bar and cooled to −30 °C for 30 min. While stirring, KC_8 (5.9 mg, 43.6 µmol, 1.00 eq) is added to the stirring solution and warmed slowly to 25 °C. After stirring for 40 min at 25 °C, the suspension is filtered followed by layering of the filtrate with hexane (15 mL). After three days at –30 °C, the supernatant is decanted off and the crystalline residue is washed with hexane (4 x 5 mL). Removing residual solvent in vacuo yields **3** as dark turquoise crystals (50.5 mg, 38.1 µmol, 87%).

See **Figure S9** and **Figure S10** for NMR and IR spectra, respectively.

Crystalline **3** displays low solubility in THF and consequently no peaks for **3** could be detected via NMR spectroscopy. The cation (δ_H = 3.33, 2.38, 2.13 ppm) as well as the co-crystallized THF (δ_H = 3.62, 1.77 ppm) can be identified in the ^{1}H NMR spectrum (see **Figure S9**). Attempted measurements in other organic solvents either lead to decomposition or insufficient solubility was observed.

ATR-IR (solid): $\tilde{\nu}$ (cm^{-1}) = 2952.2 (s), 2856.8 (m), 1437.1 (s), 1287.2(m), 1100.4 (s), 1075.8 (s).

Elem. Anal. found (calcd) for ($C_{66}H_{102}BiKN_5O_7$): C, 59.92 (59.80); H, 7.96 (7.76); N, 5.07 (5.28).

Synthesis of [K@222-crypt][Ph*O]

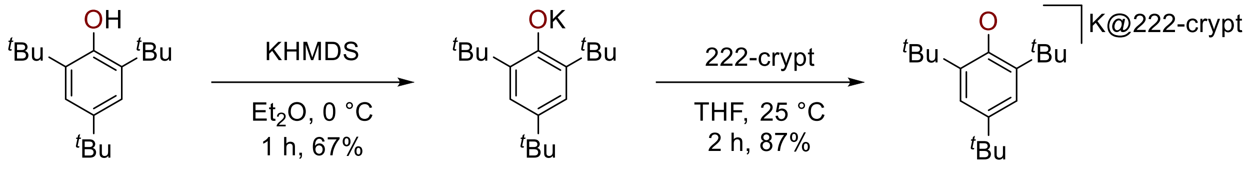

2,4,6-Tri-tert-butylphenol (1.00 g, 3.81 mmol, 1.00 eq.) is dissolved in Et_2O (30 mL) and cooled to −20 °C. A solution of potassium bis(trimethylsilyl)amide (690 mg, 3.46 mmol, 0.91 eq.) in Et_2O (30 mL) is added dropwise under intense stirring. The reaction solution is slowly warmed to 25 °C and stirred for 1 h. Volatiles are removed under reduced pressure and the white residue is washed with hexane (4 x 10 mL) and dried in vacuo overnight to afford crude K[Ph*O] as a white powder (765 mg, 2.55 mmol, 67%).

Potassium 2,4,6-tri-tert-butylphenolate (40.8 mg, 136 µmol, 1.00 eq.) and 222-crypt (46.7 mg, 143 µmol, 1.05 eq.) are added to a scintillation vial and dissolved in THF (5 mL) followed by stirring for 10 minutes. The stirring bar is removed and the reaction mixture is layered with hexane and stored at −30 °C for three days. The supernatant is decanted and the crystalline residue is washed with hexane (3 x 3 mL). After removing residual solvent in vacuo **[K@222-crypt][Ph*O]** is obtained as colorless crystals (77.5 mg, 114 µmol, 84%).

See **Figure S11 - Figure S15** and **Figure S16** for NMR and IR spectra, respectively.

NMR: (CD_2Cl_2, 25 °C) ^{1}H NMR (500 MHz) δ(ppm)= 7.28 (s, 2H, Ar*H*), 3.59 (s, 12H, 222-crypt), 3.55 – 3.50 (m, 12H, 222-crypt), 2.56 – 2.51 (m, 12H), 1.42 (s, 18H, C(C*H*$_3$)$_3$), 1.28 (s, 9H, C(C*H*$_3$)$_3$). ^{13}C{^{1}H} NMR (126 MHz) δ(ppm)= 151.17 (Ar*C*O), 146.30 (ArC_{para}(CH$_3$)$_3$), 142.69 (ArC_{ortho}(CH$_3$)$_3$), 124.04 (Ar*C*H), 70.46 (222-crypt), 67.49 (222-crypt), 53.78 (222-crypt), 35.88 (C_{ortho}*C*(C*H*$_3$)$_3$), 34.53 (C_{para}*C*(C*H*$_3$)$_3$), 31.98 (C_{ortho}C(*C*H$_3$)$_3$), 31.16 (C_{para}C(*C*H$_3$)$_3$).

ATR-IR (solid): $\tilde{\nu}$ (cm^{-1}) = 2937.8 (m) 2876.2 (s), 2812.6 (m), 1424.8 (s), 1140.5 (s), 1077.8 (s), 950.52 (s).

Elem. Anal. found (calcd) for ($C_{36}H_{65}KN_2O_7$): C, 64.00 (63.87); H, 9.70 (9.68); N, 4.10 (4.14).

One-electron oxidation of 3 by Ph*O

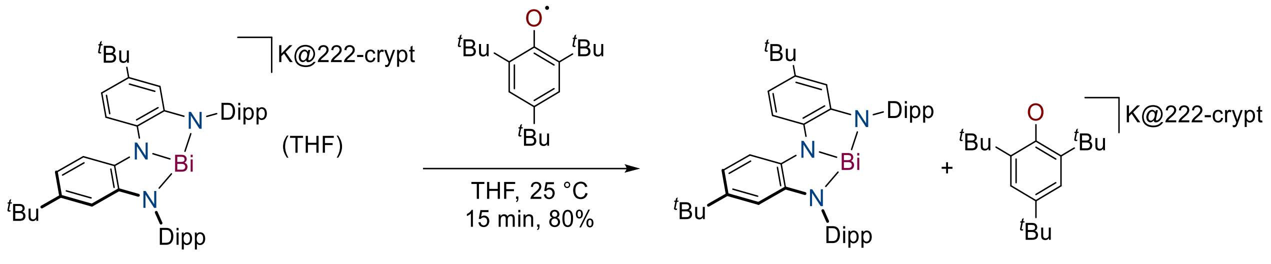

3 2

3 (30.0 mg, 22.6 µmol, 1.00 eq.) is added to a scintillation vial and suspended in THF-d_8 (0.2 mL). 2,4,6-tris-tert-butylphenoxyl radical (5.9 mg, 22.6 µmol, 1.00 eq.) is added in one portion. After shaking for 2 min at 25 °C the reaction mixture is transferred to a J-Young-NMR tube and the vial rinsed with THF-d_8 (0.3 mL). The reaction mixture is analyzed by ^{1}H NMR spectroscopy (See **Figure S17 + Figure S18**). The presence of exactly one equivalent of co-crystallized THF in **3** is confirmed by integration.

The formation of Ph*O$^-$ (δ_H = 1.20, 1.40, 6.74(superimposed) ppm) is in accordance with the literature.[11]

Unsuccessful Reaction of 3 with Gomberg's dimer

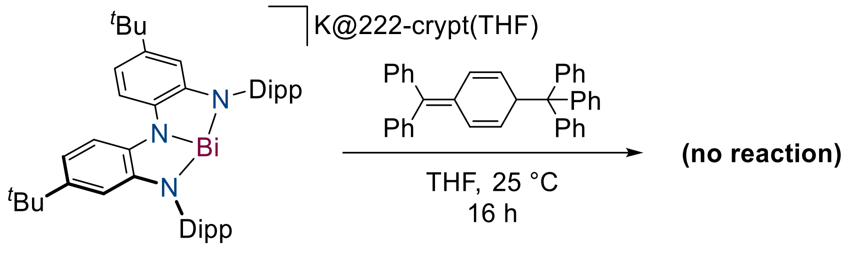

3

3 (29.1 mg, 22.9 µmol, 1.00 eq.) is suspended in THF-d8 (0.5 mL) Gomberg's dimer (5.6 mg, 11.5 µmol, 0.5 eq.) is added. The reaction solution is stirred at 25 °C for 16 h and monitored via NMR Spectroscopy. After this, an excess of the dimer (17.5 mg, 36.0 µmol, 1.5 eq.) was added. and the reaction mixture stirred for another 48 h at 25 °C. Only signals of the dimer, solvents and K@222-crypt were detected indicating that no reaction occurred.

See **Figure S19 - Figure S20** and for NMR spectra.

Synthesis of 4

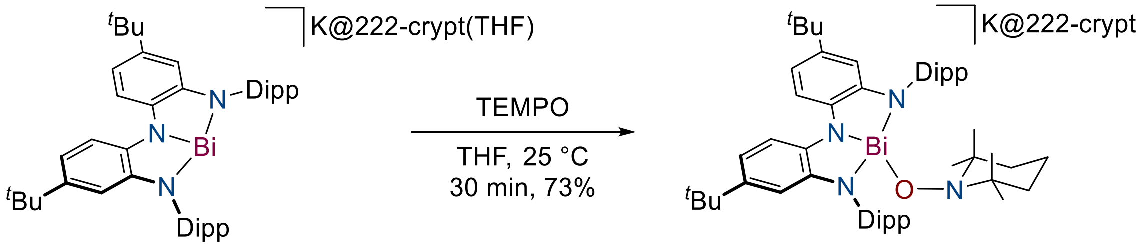

3 **4**

3 (71.2 mg, 53.7 µmol, 1.00 eq.) is suspended in THF (2 mL) and TEMPO (8.4 mg, 53.8 µmol, 1.00 eq.) is added followed by stirring for 30 min at 25 °C. Filtration followed by layering with hexane (15 mL) and storing at −30 °C for three days results in the formation of red crystals. The supernatant is decanted off and the crystalline residue is washed with hexane (4 x 5 mL). Removing residual solvent in vacuo yields **4** as dark red crystals (55.4 mg, 39.3 mmol, 73%).

The reaction solution of freshly prepared **4** was further examined by NMR-spectroscopy: **3** (30.0 mg, 22.6 µmol, 1.00 eq.) is suspended in THF-d_8 (0.7 mL) and cooled to –30 °C for 15 min. TEMPO (3.5 mg, 23.0 µmol, 1.02 eq.) is added and stirred for 15 min at 25 °C. The reaction solution is filtered into a J.-Young NMR tube and analyzed by NMR spectroscopy, showing quantitative formation of **4**. The presence of exactly one equivalent of co-crystallized THF in **3** is demonstrated by integration. The red solution is filtered, layered with hexane (5 mL) and storing at −30 °C for recrystallization.

The ^{1}H NMR of **4** displays significant broadening in the high field region . However, integration over this area showed a total of 60 proton signals in line with the proposed structure. One C*H*(CH_3)$_2$ group is only visible by measurement at –15 °C.

See **Figure S21 - Figure S23** and **Figure S24** for NMR and IR spectra, respectively.

NMR: (THF-d_8, –15 °C) ^{1}H NMR (400 MHz) δ(ppm)= 7.62 (d, *J* = 8.2 Hz, 1H, Ar*H*), 7.47 (d, *J* = 8.2 Hz, 1H, Ar*H*), 7.15 (d overlapping, *J* = 7.6 Hz, 2H, DippC*H*), 7.08 (d overlapping, *J* = 7.6 Hz, 2H, DippC*H*), 6.95 (t, *J* = 7.6 Hz, 1H, DippC*H*), 6.87 (t, *J* = 7.6 Hz, 1H, DippC*H*), 6.18 (d, *J* = 8.2 Hz, 1H Ar*H*), 6.14 (d, *J* = 8.2 Hz, 1H, Ar*H*), 5.75 (s, 2H, Ar*H*), 4.57 (s, 1H, C*H*(CH_3)$_2$), 3.66 (hept, *J* = 6.7 Hz, 1H, C*H*(CH_3)$_2$), 3.51 (s, 12H, 222-crypt), 3.49 – 3.44 (m, *J* = 4.5 Hz, 12H, 222-crypt), 3.06 (hept, *J* = 6.7 Hz, 1H, C*H*(CH_3)$_2$), 2.72 (s, 1H, C*H*(CH_3)$_2$), 2.55 – 2.44 (m, *J* = 4.5 Hz, 12H), 1.57 – 1.48 (m, 2H, TEMPO-C*H*$_2$), 1.44 (s, 2H, TEMPO-C*H*$_2$), 1.34 – 1.20 (m overlapping*, 2H, TEMPO-C*H*$_2$), 1.14 (s, 12H, TEMPO-C(C*H*$_3$)$_2$), 1.11 (s, 18H, C(C*H*$_3$)$_3$), 1.07 – 0.96 (m overlapping, *J* = 6.7 Hz, 12H, CH(C*H*$_3$)$_2$), 0.93 – 0.78 (m overlapping*, 12H, CH(C*H*$_3$)$_2$).*hexane impuritiy

ATR-IR (solid): $\tilde{\nu}$ (cm^{-1}) = 2954.2(m), 1283.1(s), 1248,2(s), 1102.4(s), 950.52(s)

Elem. Anal. found (calcd) for ($C_{71}H_{112}BiKN_6O_7$): C, 60.30 (60.49); H, 7.98 (8.01); N, 5.70 (5.96).

Synthesis of 5

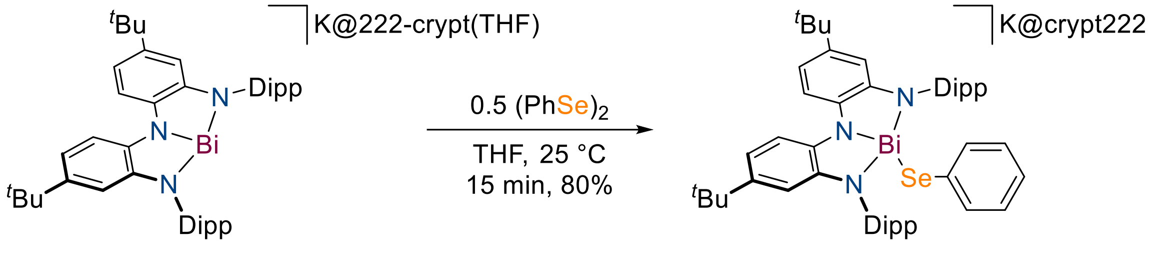

3 (63.1 mg, 47.6 µmol, 1.00 eq.) is suspended in THF (2 mL) and diphenyl diselenide (7.43 mg, 23.8 µmol, 0.50 eq.) is added followed by stirring for 15 min at 25 °C. Filtration followed by layering with hexane (15 mL) and storing at −30 °C for three days results in the formation of dark red crystals. The supernatant is decanted off and the crystalline residue is washed with hexane (4 x 5 mL). Removing residual solvent in vacuo yields **5** as dark red crystals (53.4 mg, 37.9 mmol, 80%).

The reaction was further followed by NMR-spectroscopy: **3** (24.0 mg, 18.1 µmol, 1.00 eq.) and diphenyl diselenide (2.8 mg, 9.05 µmol, 0.50 eq.) are suspended in THF-d_8 (0.5 mL), shaken for 2 minutes and analyzed by ^{1}H NMR spectroscopy showing quantitative formation of **5**. The presence of exactly one equivalent of co-crystallized THF in **3** is demonstrated by integration. No ^{77}Se{^{1}H} NMR signals could be detected, likely due to fast relaxation and large line broadening caused by the presence of Bi.

See **Figure S25 - Figure S31** and **Figure S32** for NMR and IR spectra, respectively.

NMR: (THF-d_8, 25 °C) ^{1}H NMR (600 MHz) δ(ppm)= 7.58 (d, *J* = 8.3 Hz, 2H, Ar*H*), 7.00 (d, *J* = 7.7 Hz, 4H, Ar*H*), 6.87 – 6.84 (m, 2H, DippC*H*), 6.84 (t, *J* = 7.7 Hz, 2H, DippC*H*), 6.54 – 6.47 (m, 3H), 6.13 (dd, *J* = 8.3, *J* = 2.4 Hz, 2H, Ar*H*), 5.77 (d, *J* = 2.4 Hz, 2H, Ar*H*), 3.39 (s, 12H, 222-crypt), 3.36 – 3.32 (m, 12H, 222-crypt), 3.21 (hept, *J* = 6.9 Hz, 4H, (C*H*(CH_3)$_2$), 2.39 – 2.34 (m, 12H, 222-crypt), 1.05 (s, 18H, C(CH_3)$_3$), 1.01 (d, *J* = 6.9 Hz, 12H, CH(CH_3)$_2$), 0.96 (d, *J* = 6.8 Hz, 12H, CH(CH_3)$_2$). ^{13}C{^{1}H} NMR (151 MHz) δ(ppm)= 151.87(ArC), 149.03(Ar^{Dipp}*C*), 146.08(Ar^{Dipp}*C*), 144.61(Ar*C*), 139.74(Ar*C*), 137.47(SeArCH), 128.04(SeAr*C*H), 124.38(Ar^{Dipp}*C*H), 123.89(Ar^{Dipp}*C*H), 123.33(SeAr*C*H), 116.30(Ar*C*H), 111.73(Ar*C*H), 108.60(Ar*C*H), 71.48(222-crypt), 68.63(222-crypt), 54.91(222-crypt), 34.54(*C*(CH_3)$_3$), 32.76(C(CH_3)$_3$), 28.24(*C*H(CH_3)$_2$), 26.70(CH(*C*H_3)$_2$), 26.11(CH(*C*H_3)$_2$).

ATR-IR (solid): $\tilde{\nu}$ (cm^{-1}) = 2954.2 (m), 2861.8 (m), 1435.0 (s) 1287.2 (s), 1100.4 (s), 948.5 (s).

Elem. Anal. found (calcd) for ($C_{68}H_{99}BiKN_5O_6Se$): C, 57.92 (57.94); H, 6.94 (7.08); N, 4.93 (4.97).

NMR and IR spectroscopy

NMR and IR spectroscopy of 1

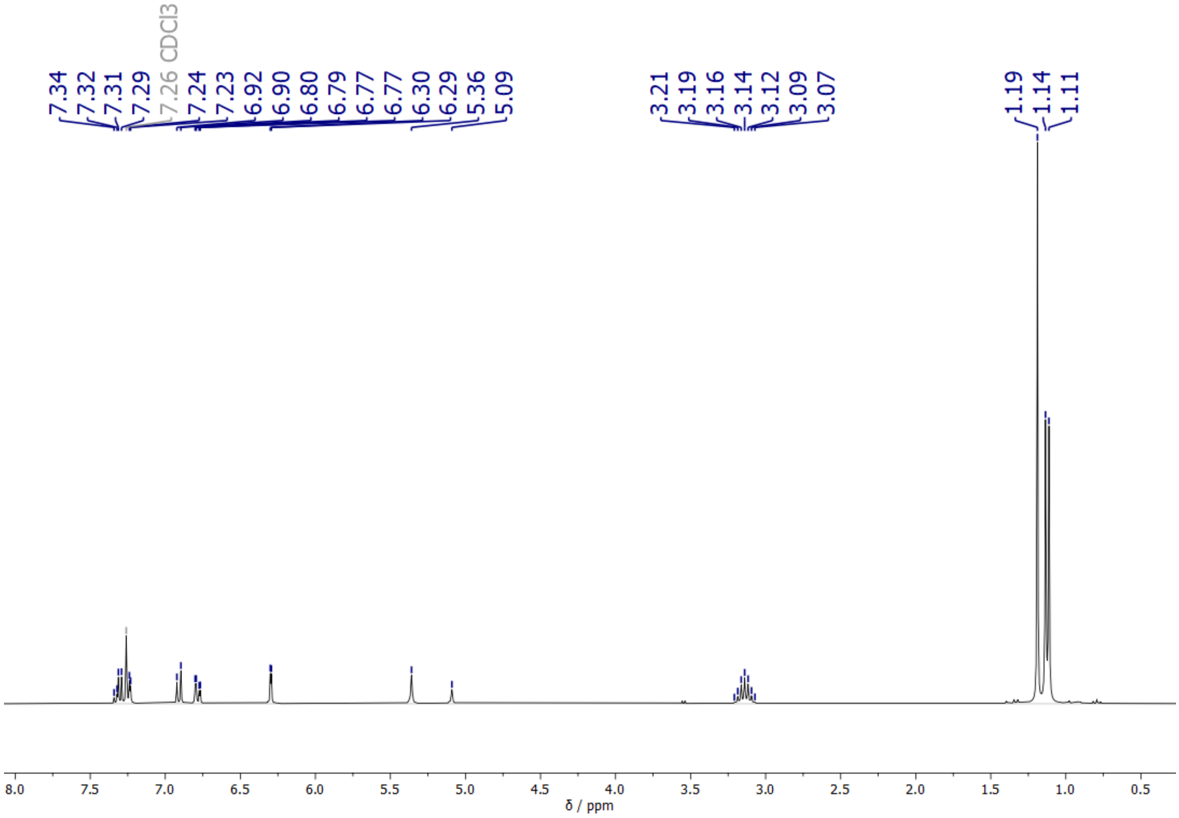

Figure S1. ^{1}H NMR spectrum of **1**, $CDCl_3$, 25 °C.

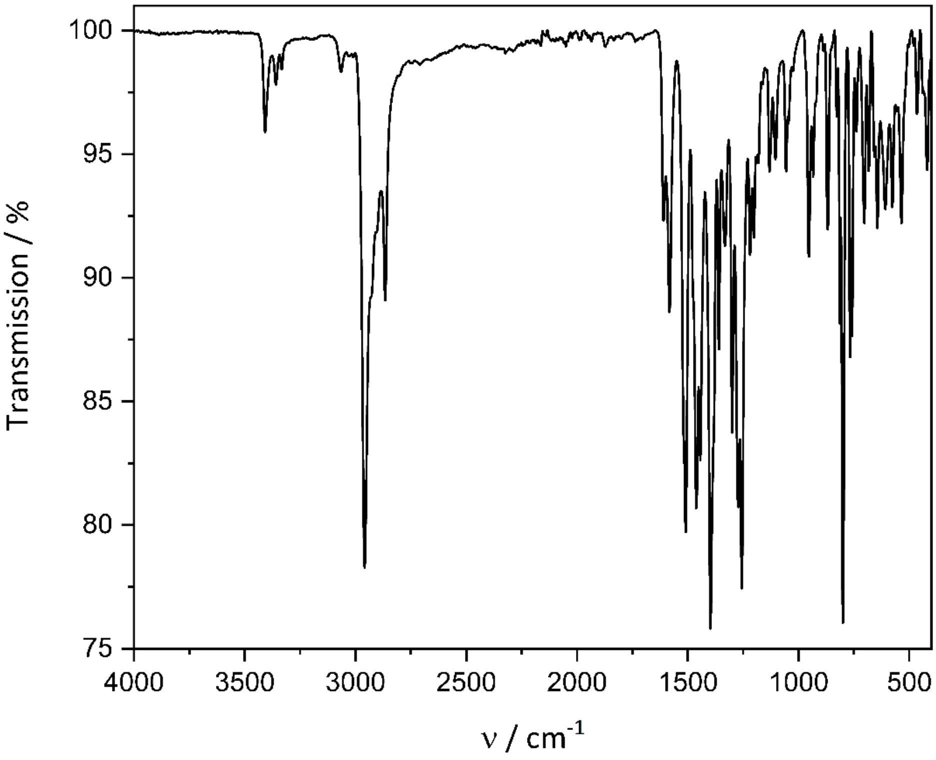

Figure S2. ATR-IR spectrum of **1**, solid, 25 °C.

NMR and IR spectroscopy of 2

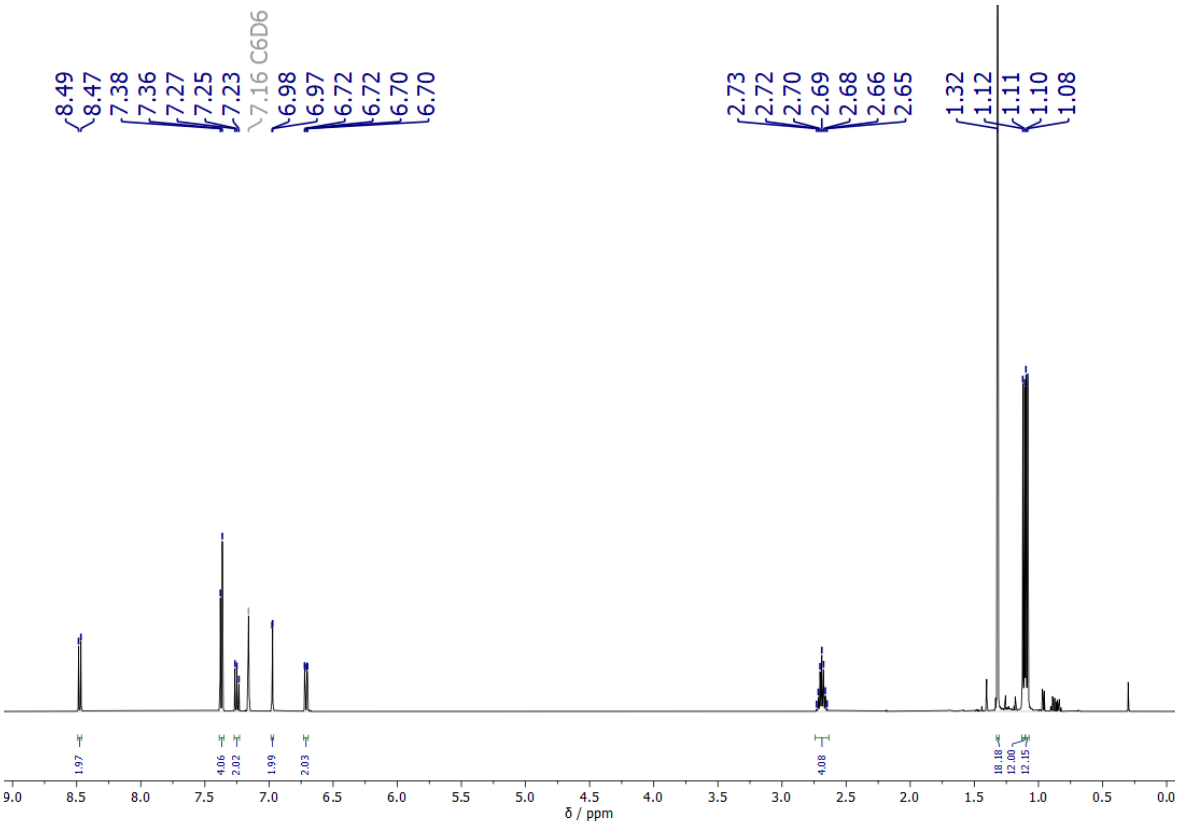

Figure S3. ^{1}H NMR spectrum of **2**, C_6D_6, 25 °C.

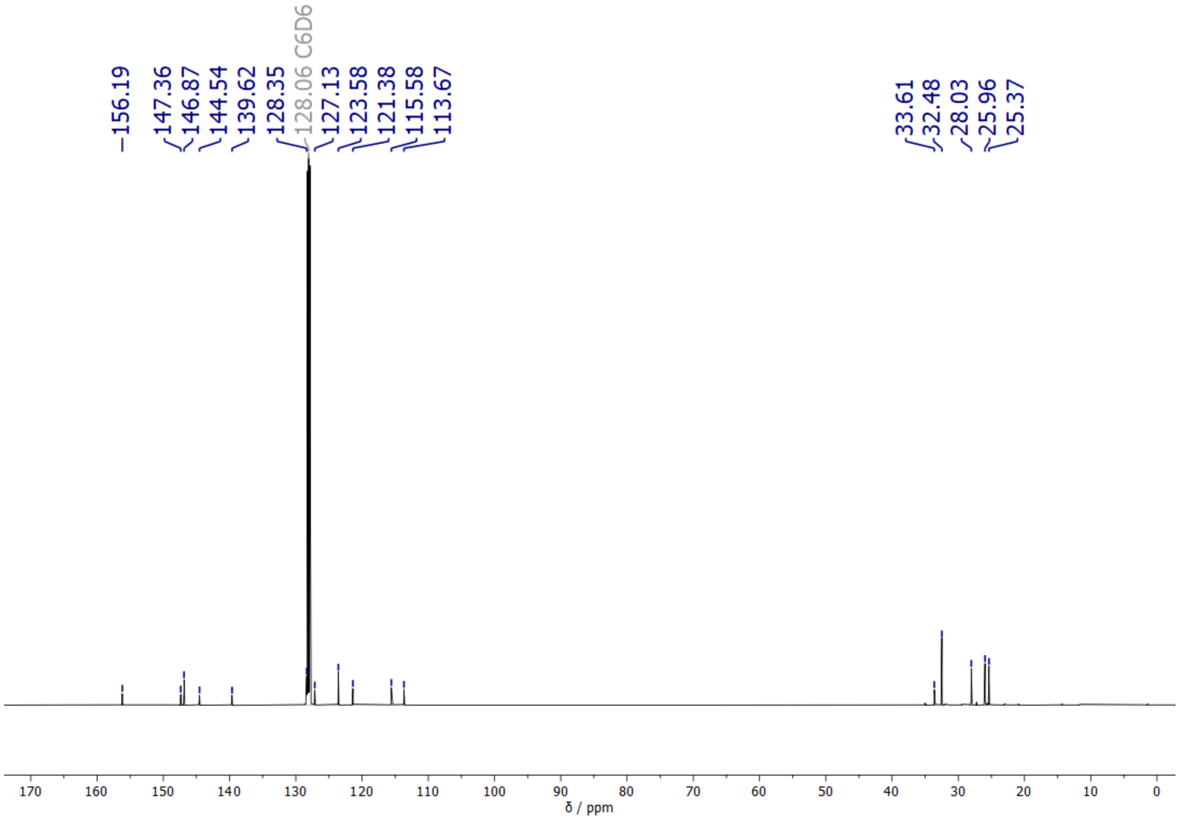

Figure S4. $^{13}C\{^{1}H\}$ NMR spectrum of **2**, C_6D_6, 25 °C.

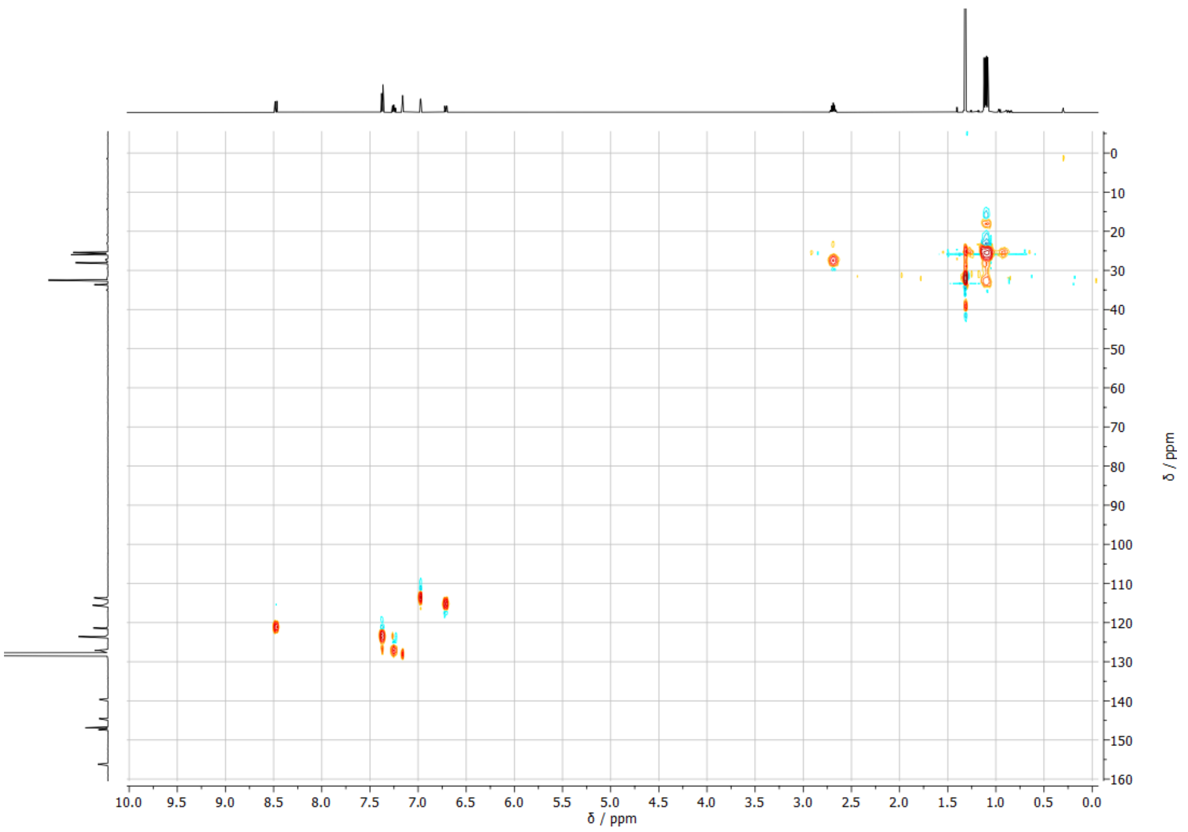

Figure S5. HSQC NMR spectrum of **2**, C_6D_6, 25 °C.

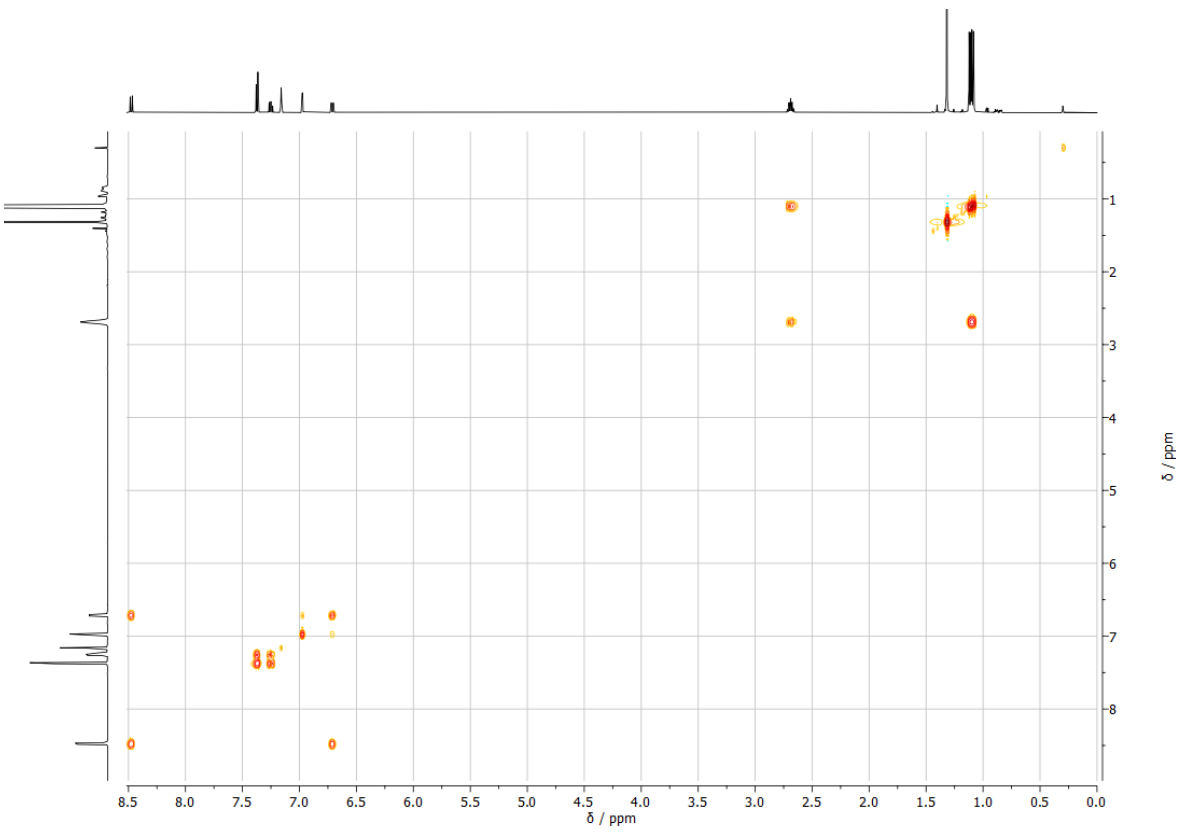

Figure S6. COSY NMR spectrum of **2**, C_6D_6, 25 °C.

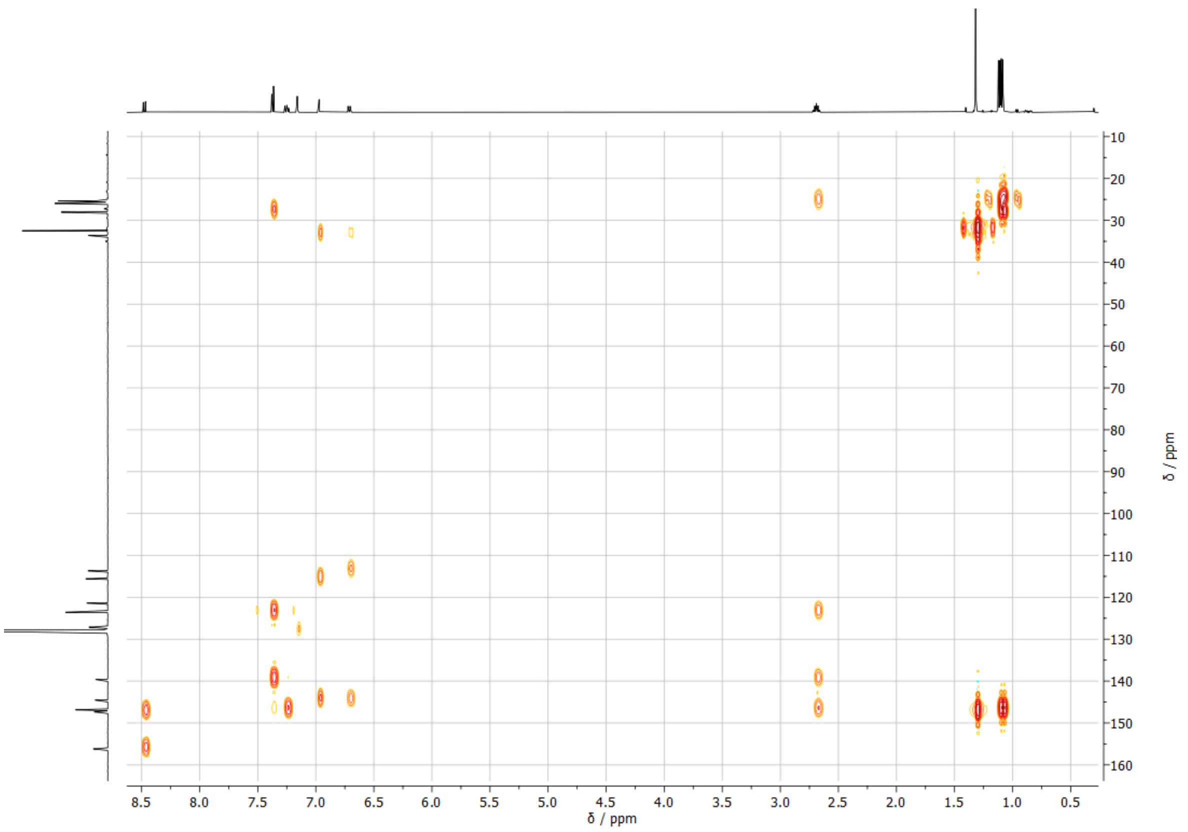

Figure S7. HMBC NMR spectrum of **2**, C_6D_6, 25 °C.

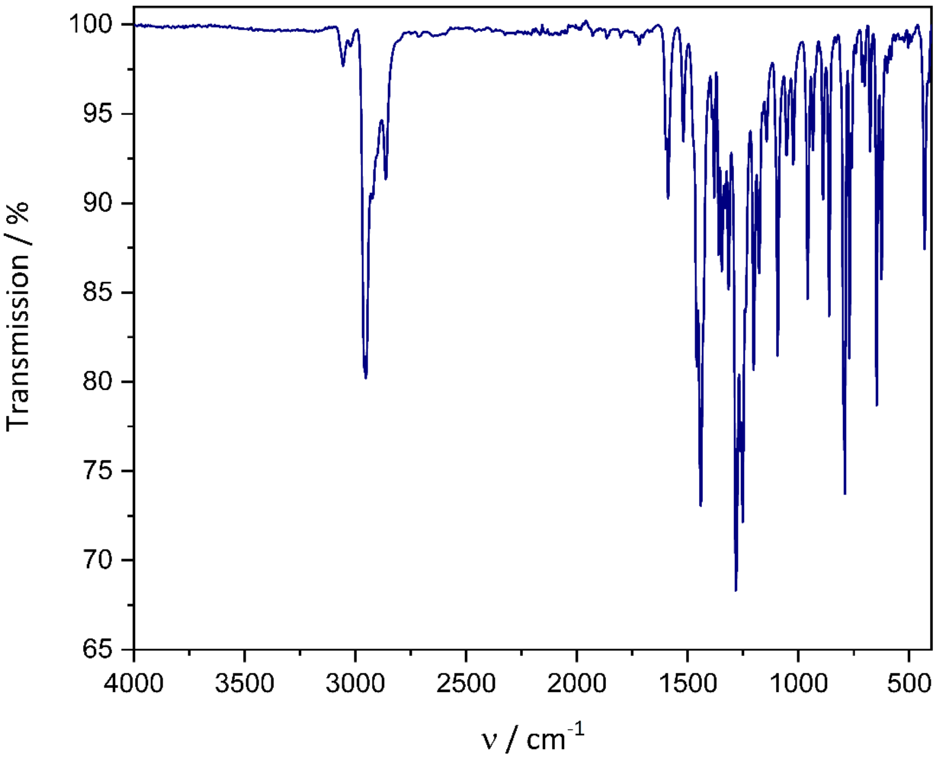

Figure S8. ATR-IR spectrum of **2**, solid, 25 °C.

NMR and IR spectroscopy of 3

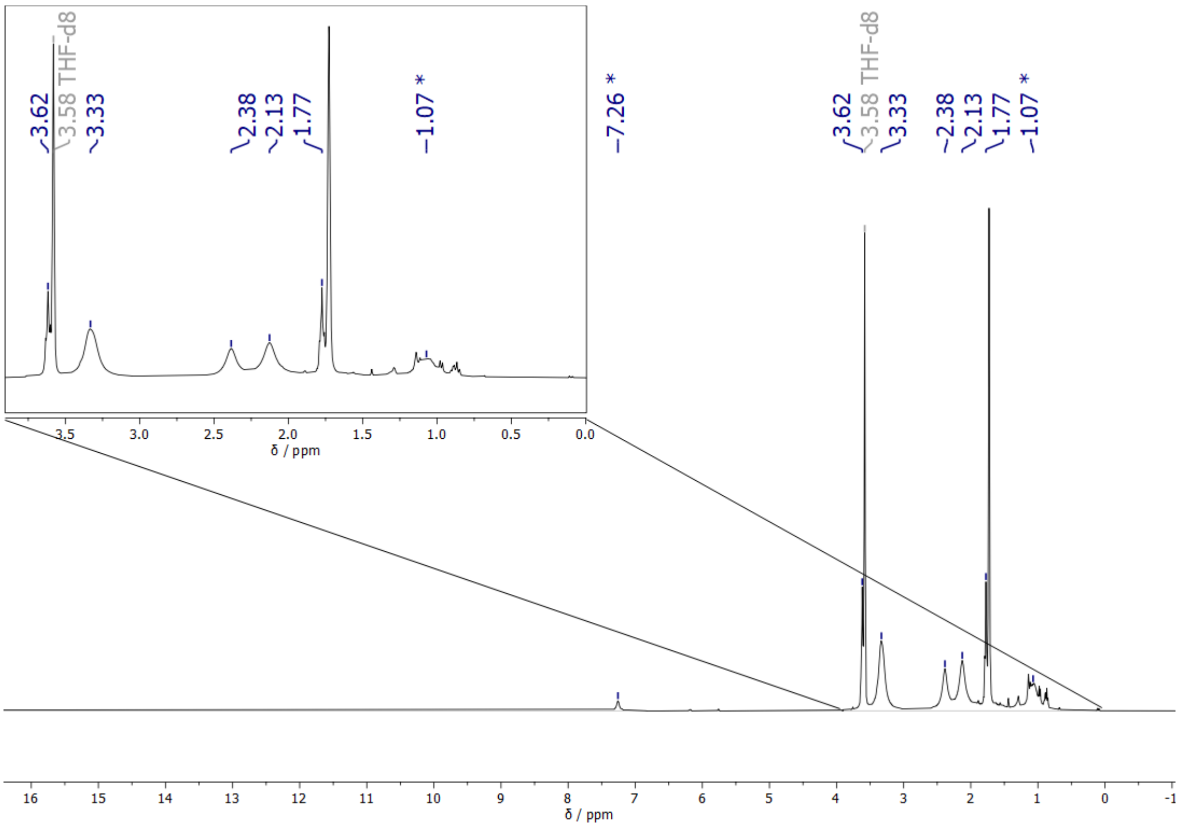

Figure S9. ^{1}H NMR spectrum of **3**, THF-d_8, 25 °C. *Minor Impurities hexane, H-grease.

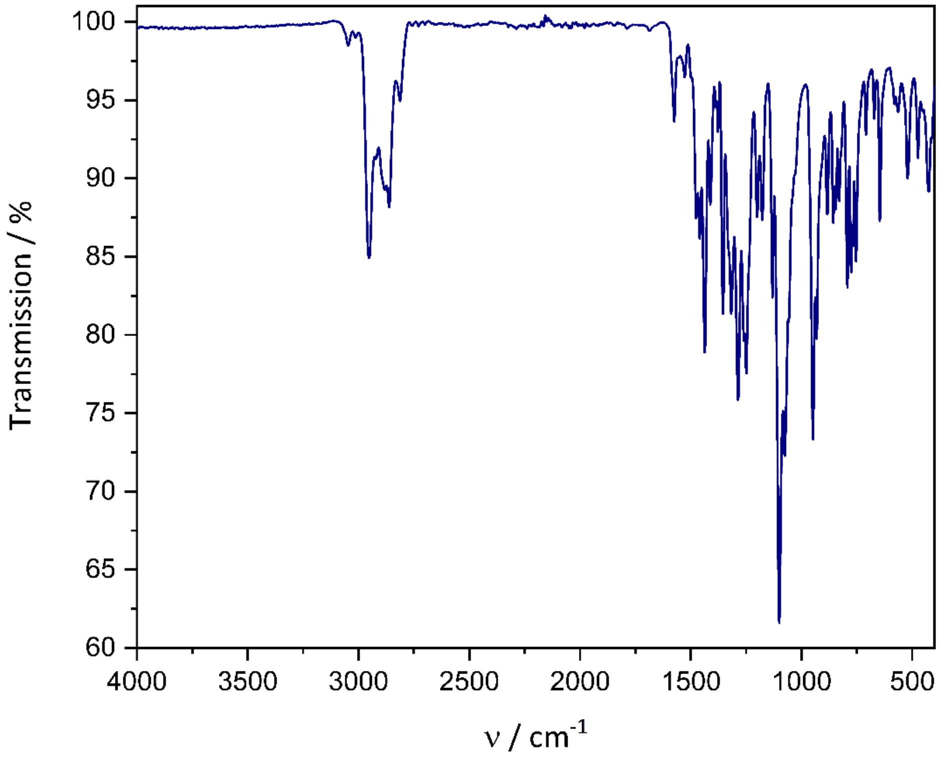

Figure S10. ATR-IR spectrum of **3**, solid, 25 °C.

NMR spectroscopy of [K@222-crypt][Ph*O]

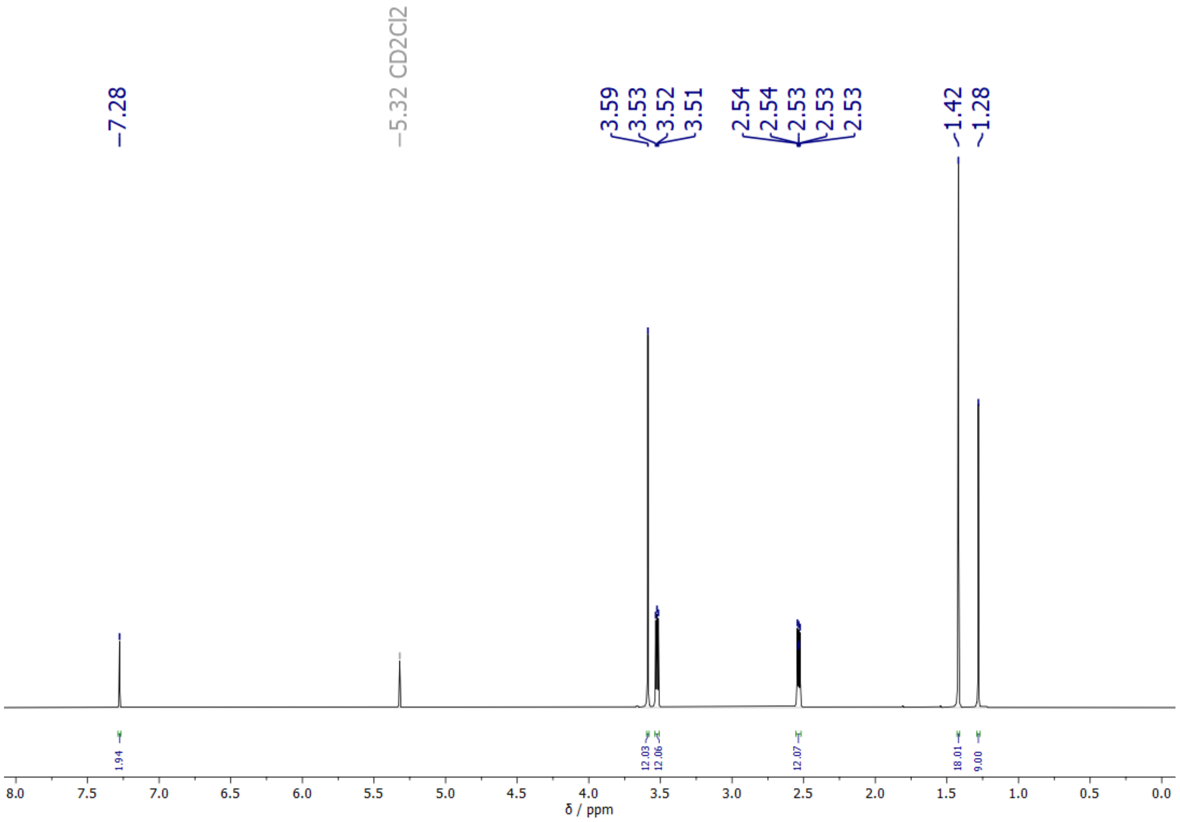

Figure S11. ^{1}H NMR spectrum of **[K@222-crypt][Ph*O]**, CD_2Cl_2, 25 °C.

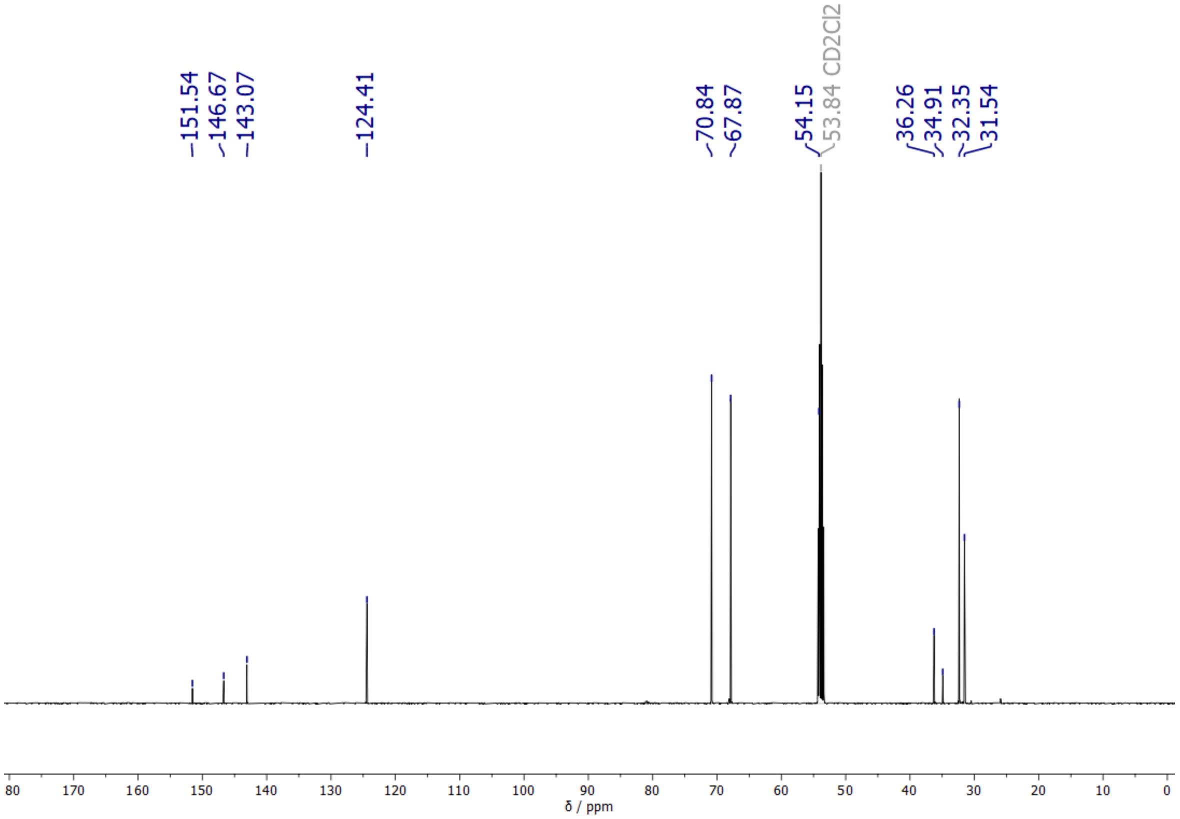

Figure S12. $^{13}C\{^{1}H\}$ NMR spectrum of **[K@222-crypt][Ph*O]**, CD_2Cl_2, 25 °C.

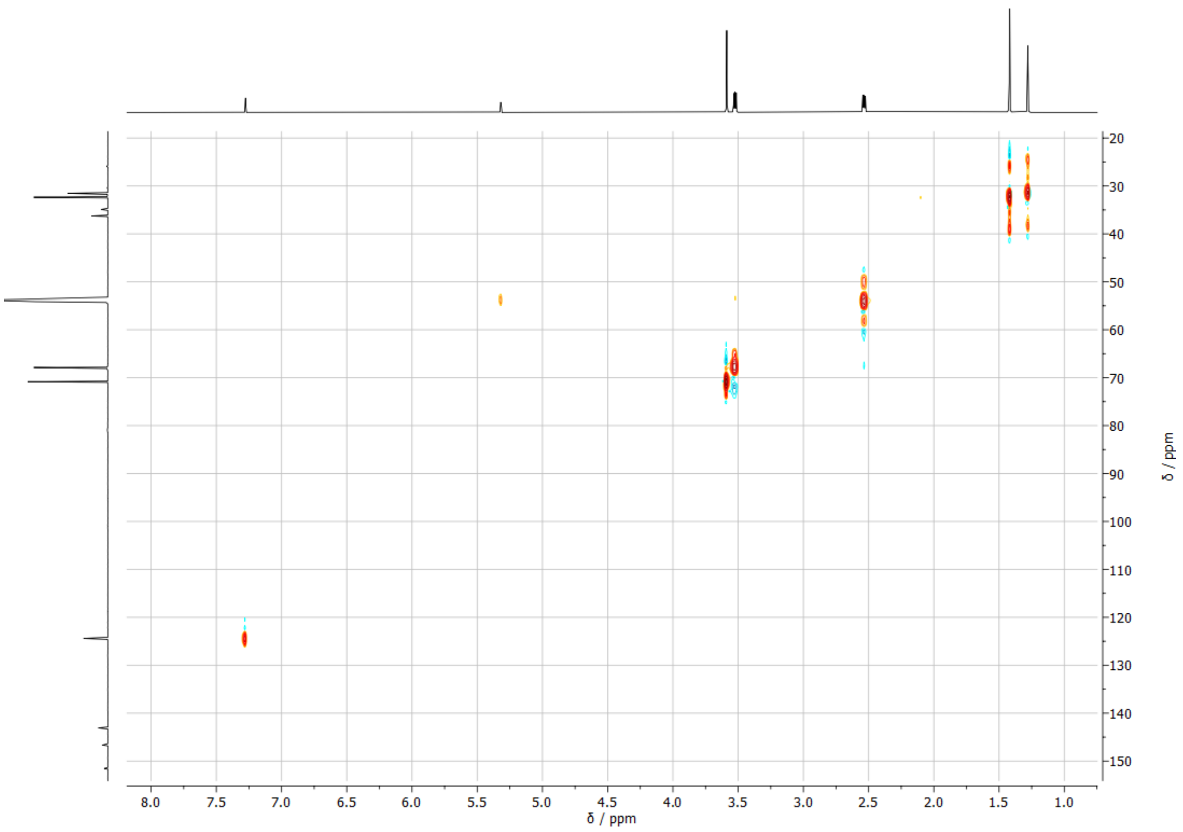

Figure S13. HSQC NMR spectrum of **[K@222-crypt][Ph*O]**, CD_2Cl_2, 25 °C.

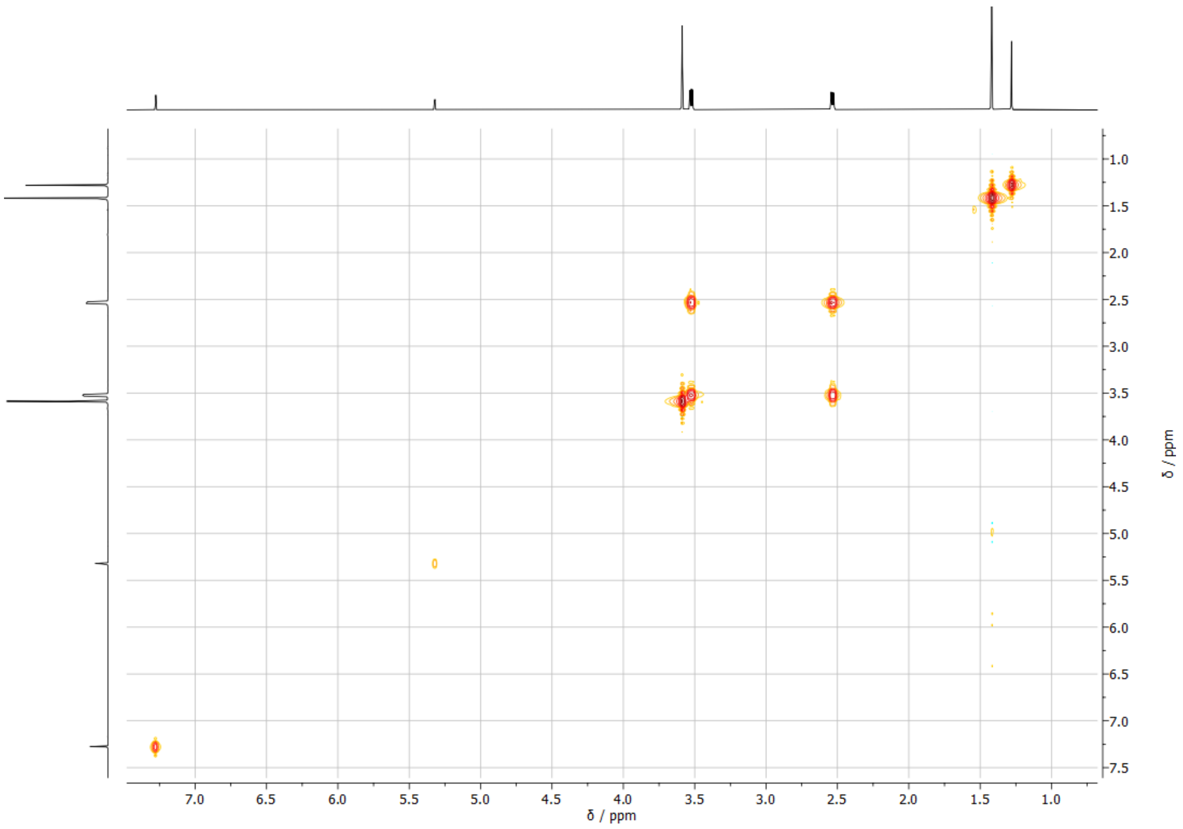

Figure S14. COSY NMR spectrum of **[K@222-crypt][Ph*O]**, CD_2Cl_2, 25 °C.

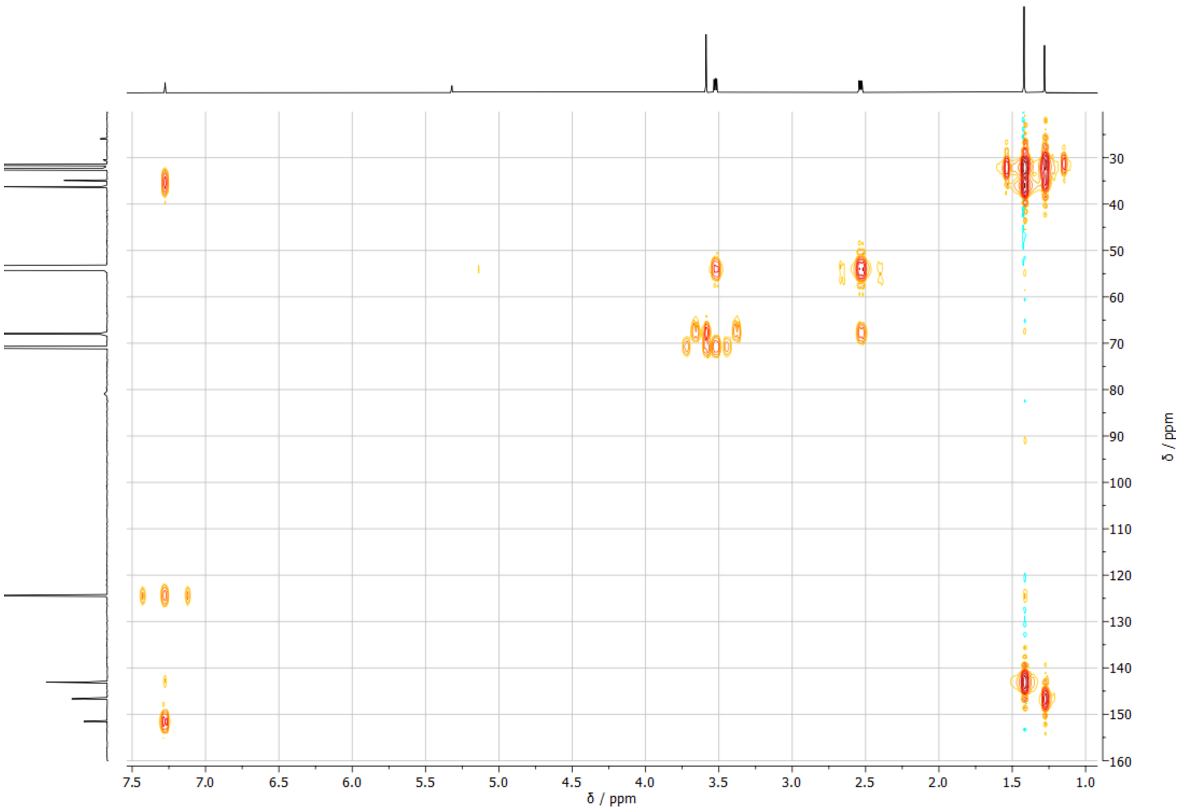

Figure S15. HMBC NMR spectrum of **[K@222-crypt][Ph*O]**, CD_2Cl_2, 25 °C.

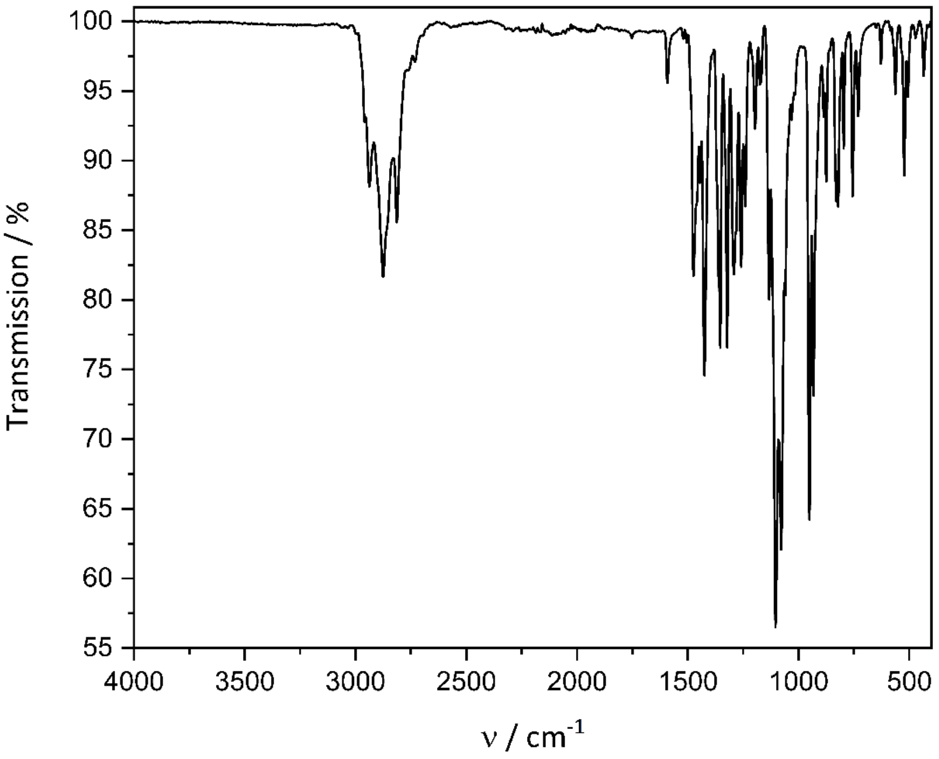

Figure S16. ATR-IR spectrum of **[K@222-crypt][Ph*O]**, solid, 25 °C.

NMR spectroscopy of the one-electron oxidation of 3 by Ph*O

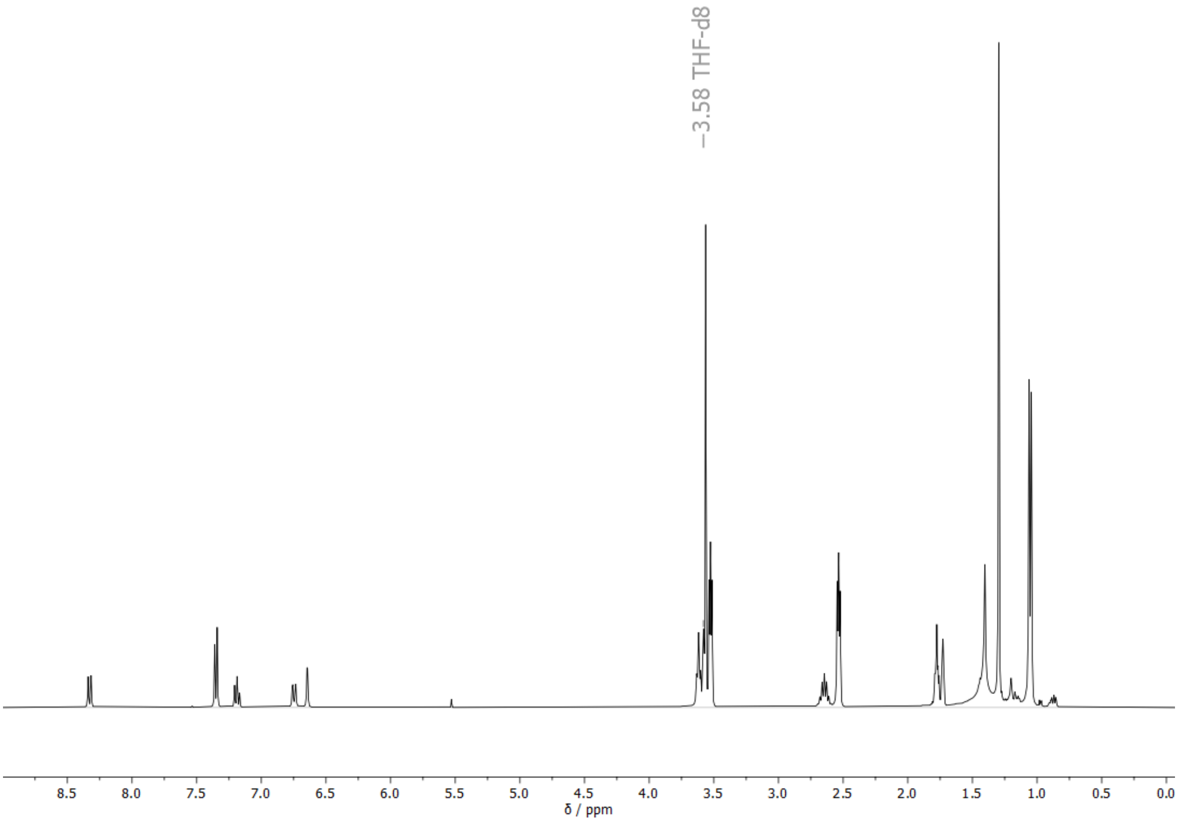

Figure S17. ^{1}H NMR spectrum of **3** with Ph*O after 5 min, THF-d_8, 25 °C.

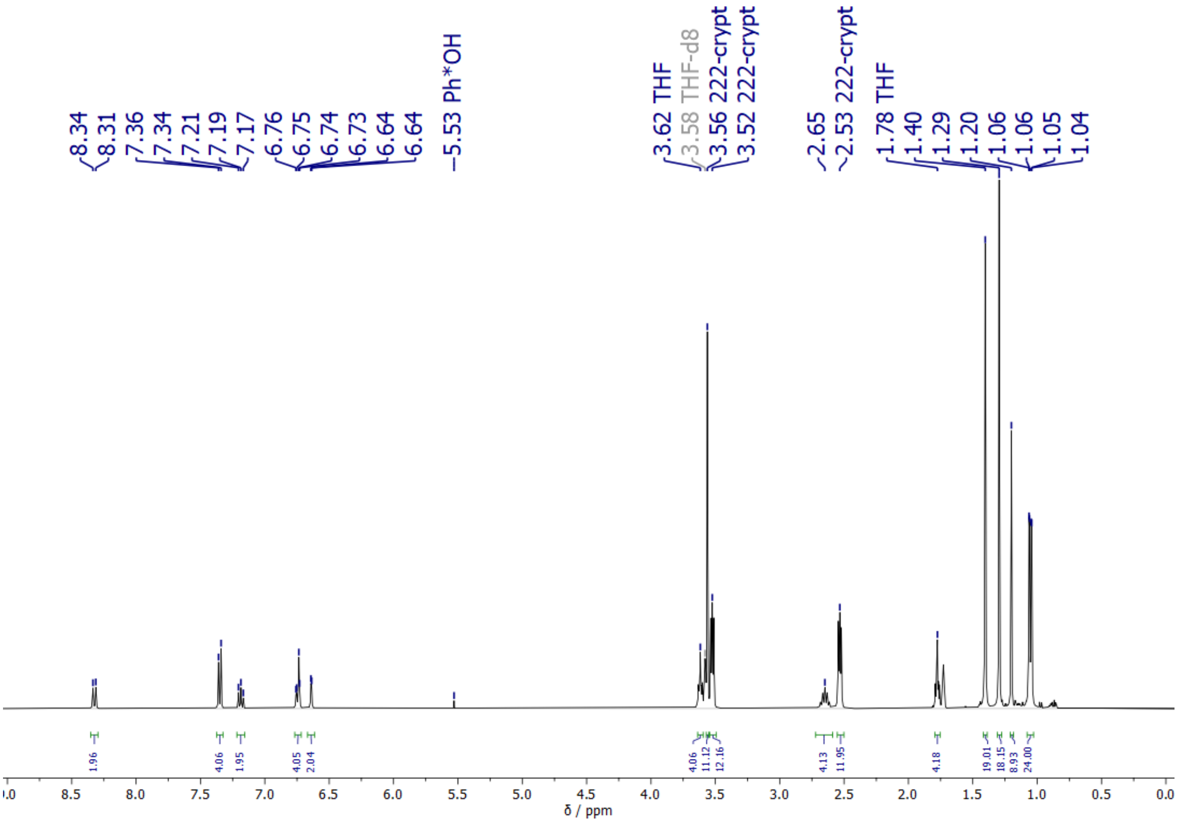

Figure S18. ^{1}H NMR spectrum of **3** with Ph*O after 1 h, THF-d_8, 25 °C.

NMR spectroscopy of 3 with Gomberg's dimer

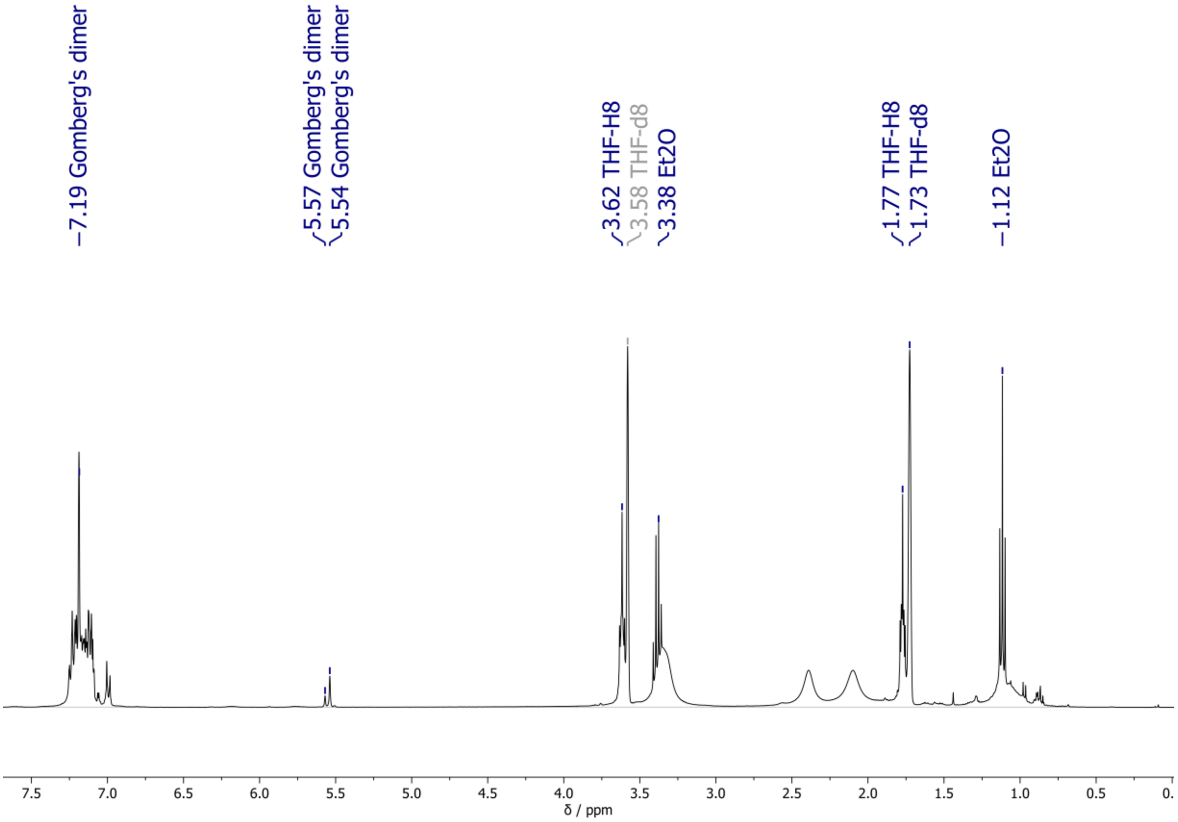

Figure S19. ^{1}H NMR spectrum of **3** with 0.5 eq of Gomberg's dimer, THF-d_8, 25 °C.

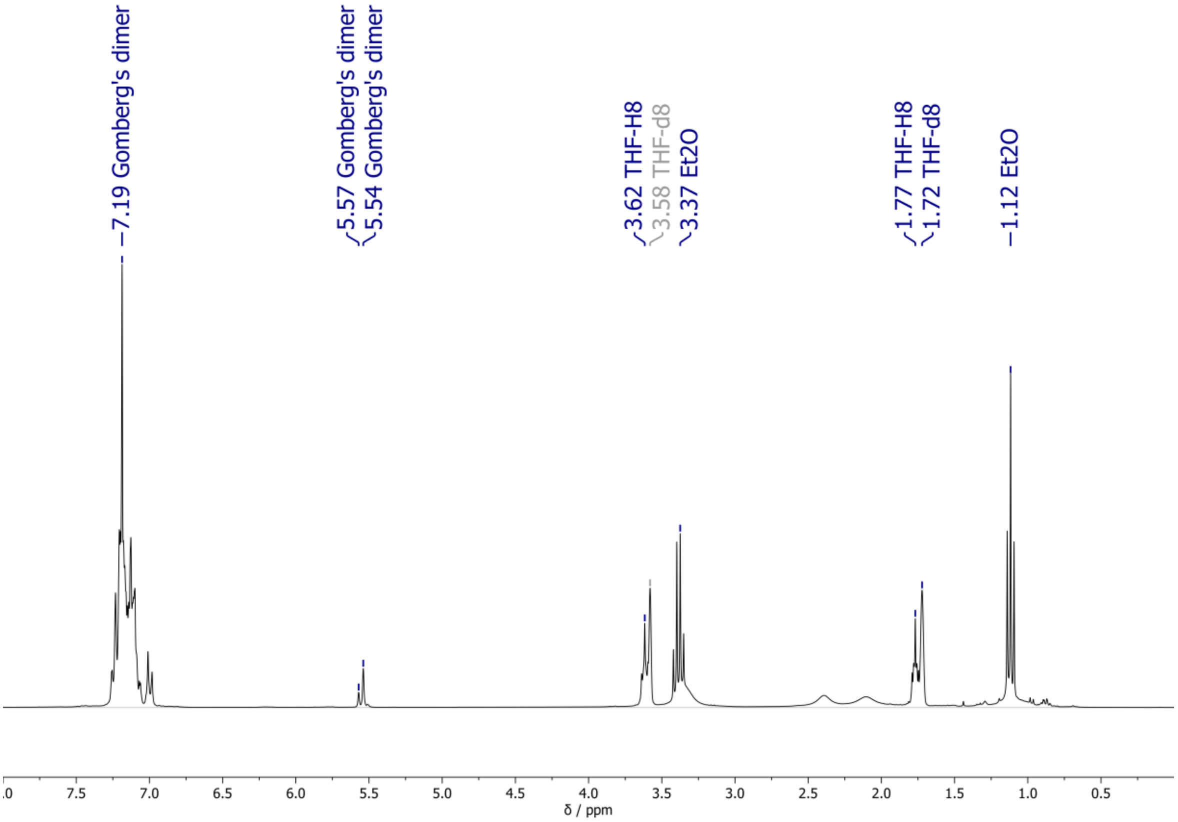

Figure S20. ^{1}H NMR spectrum of **3** with 2.0 eq of Gomberg's dimer, THF-d_8, 25 °C.

NMR and IR spectroscopy of 4

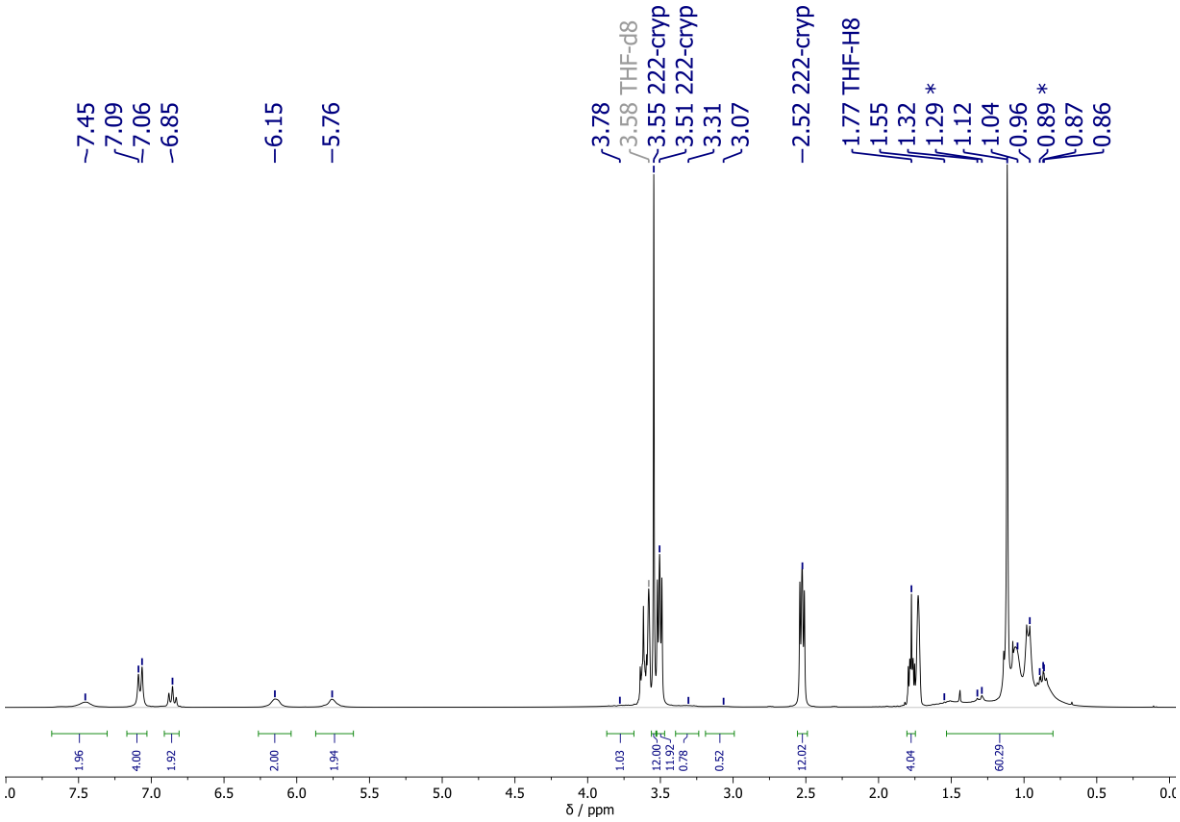

Figure S21. ^{1}H NMR spectrum of freshly prepared **4**, THF-d_8, 25 °C. Hexane is marked with *.

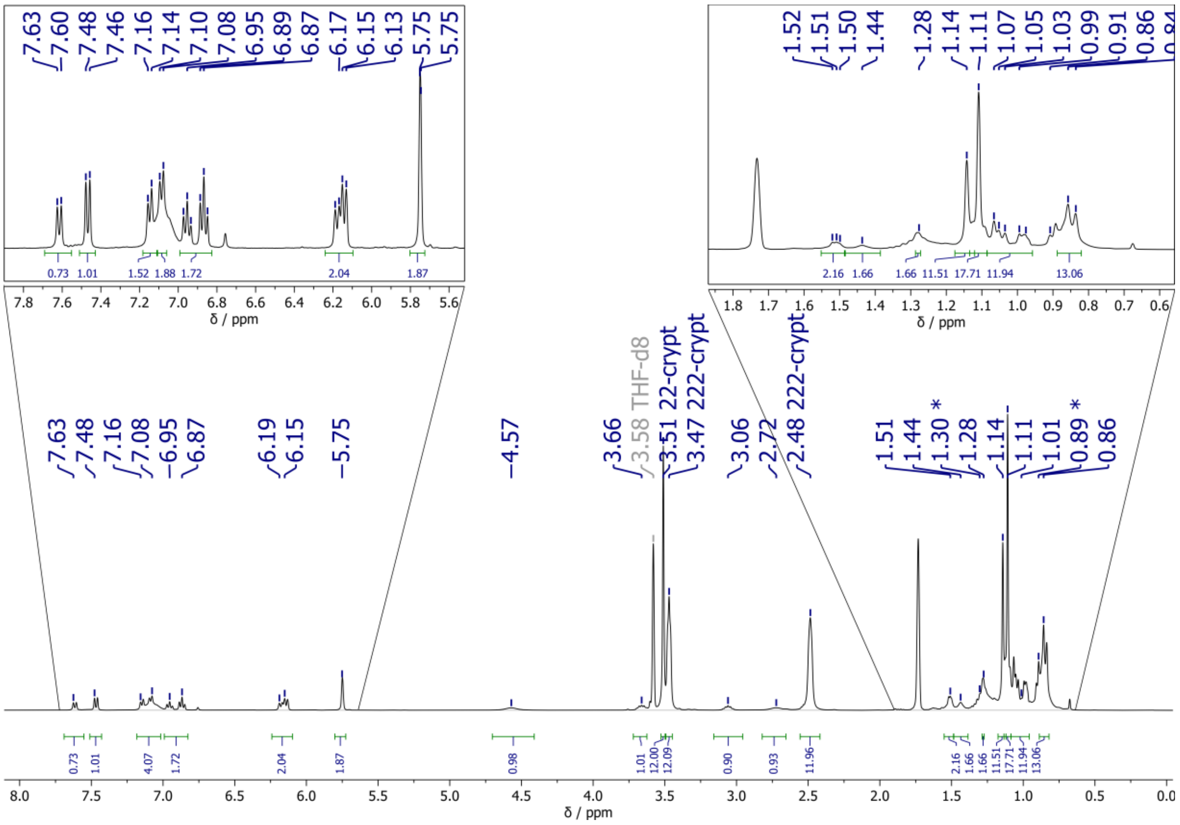

Figure S22. ^{1}H NMR spectrum of isolated **4**, THF-d_8, –15 °C. Hexane is marked with *.

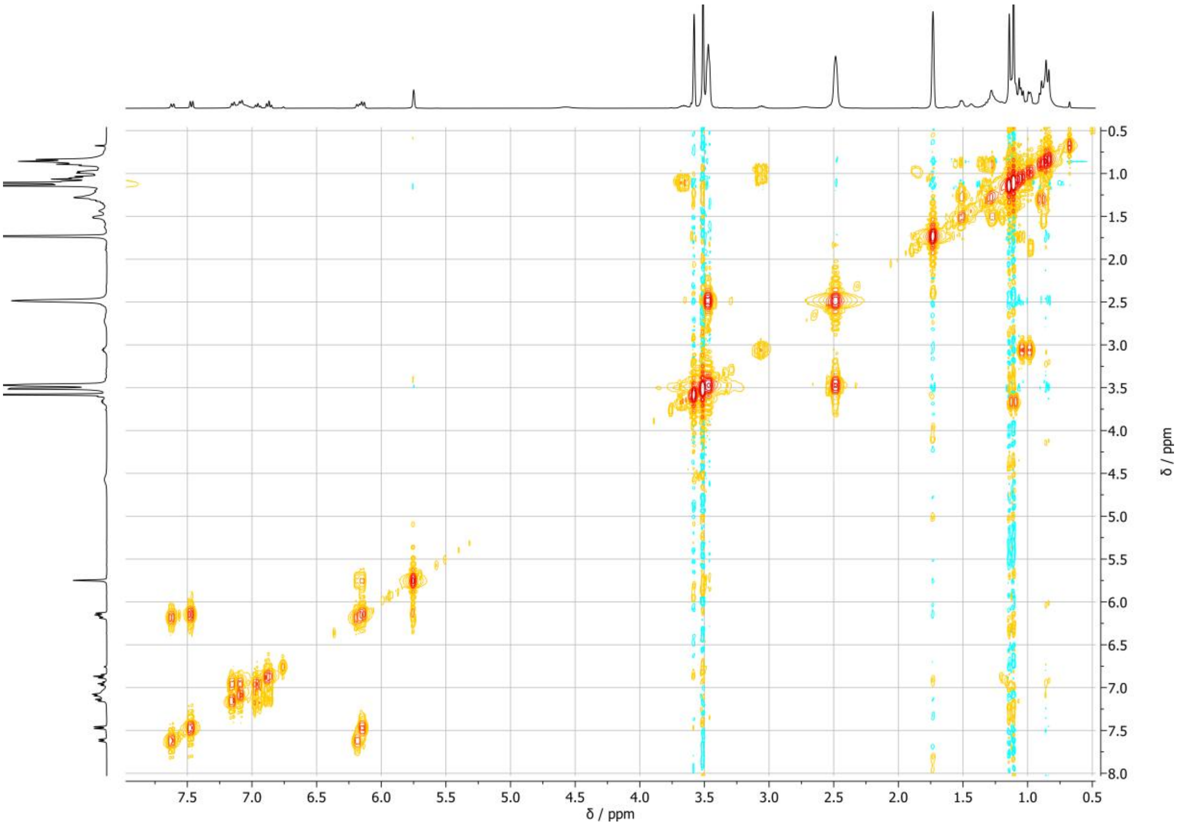

Figure S23. COSY spectrum of isolated **4**, THF-d_8, –15 °C.

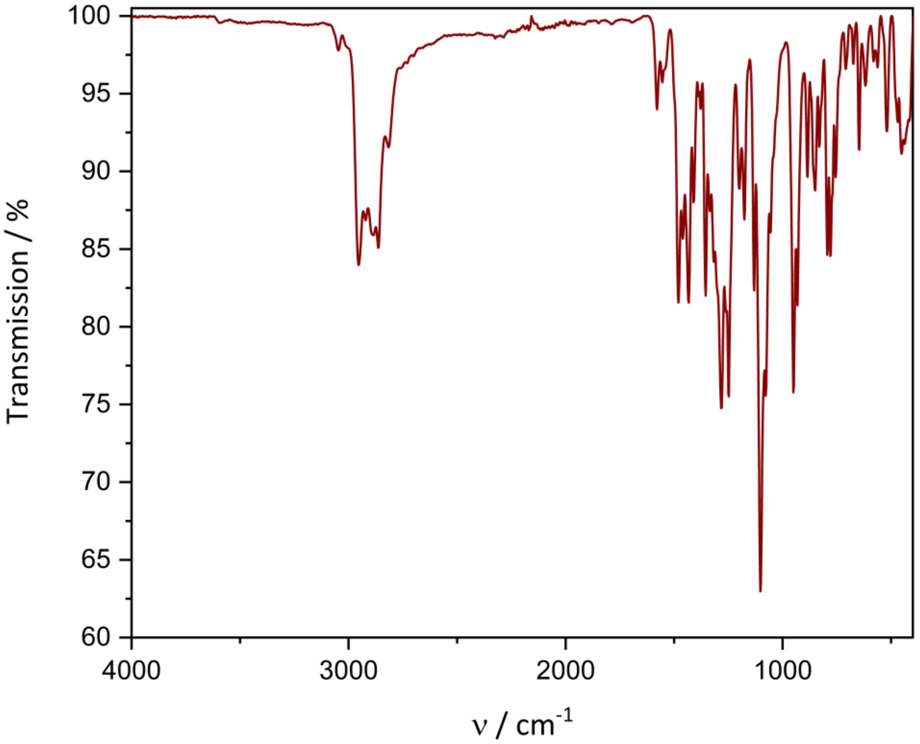

Figure S24. ATR-IR spectrum of **4**, solid, 25 °C.

NMR and IR spectroscopy of 5

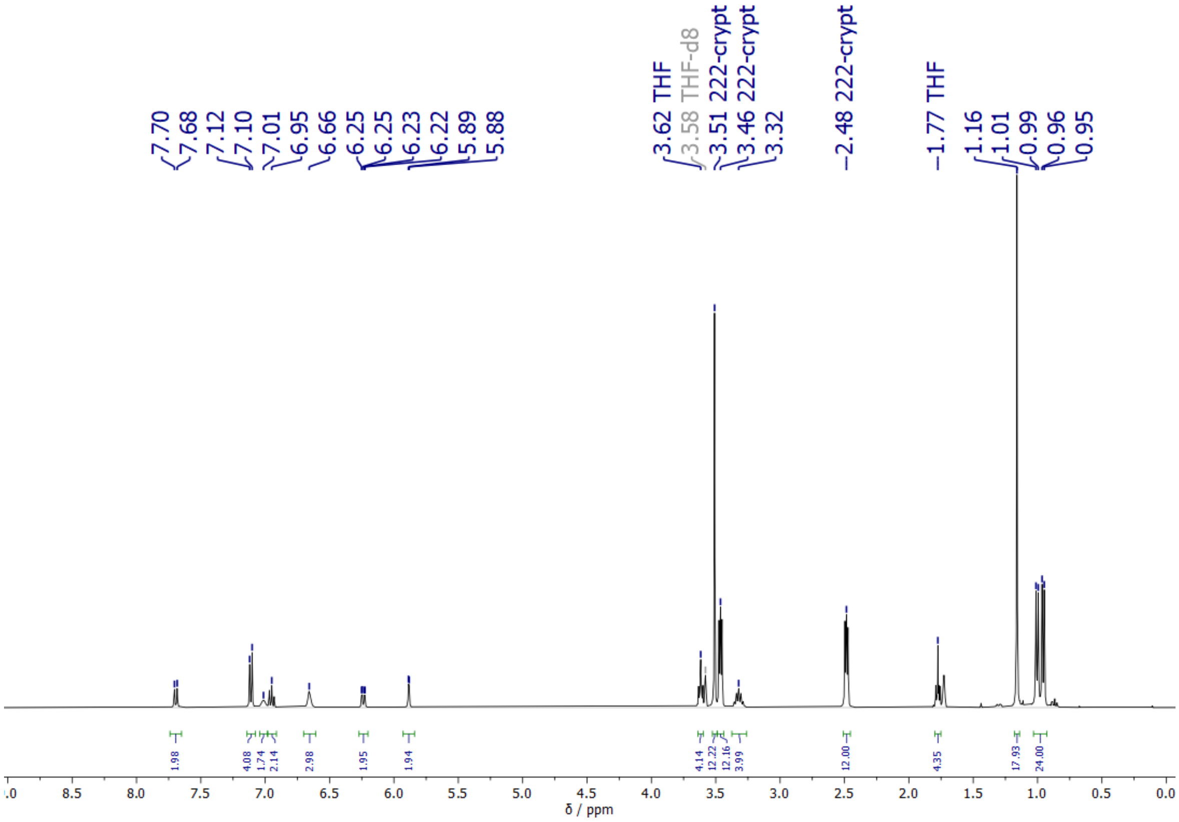

Figure S25. ^{1}H NMR spectrum of **5** with $(PhSe)_2$ after 30 min, THF-d_8, 25 °C.

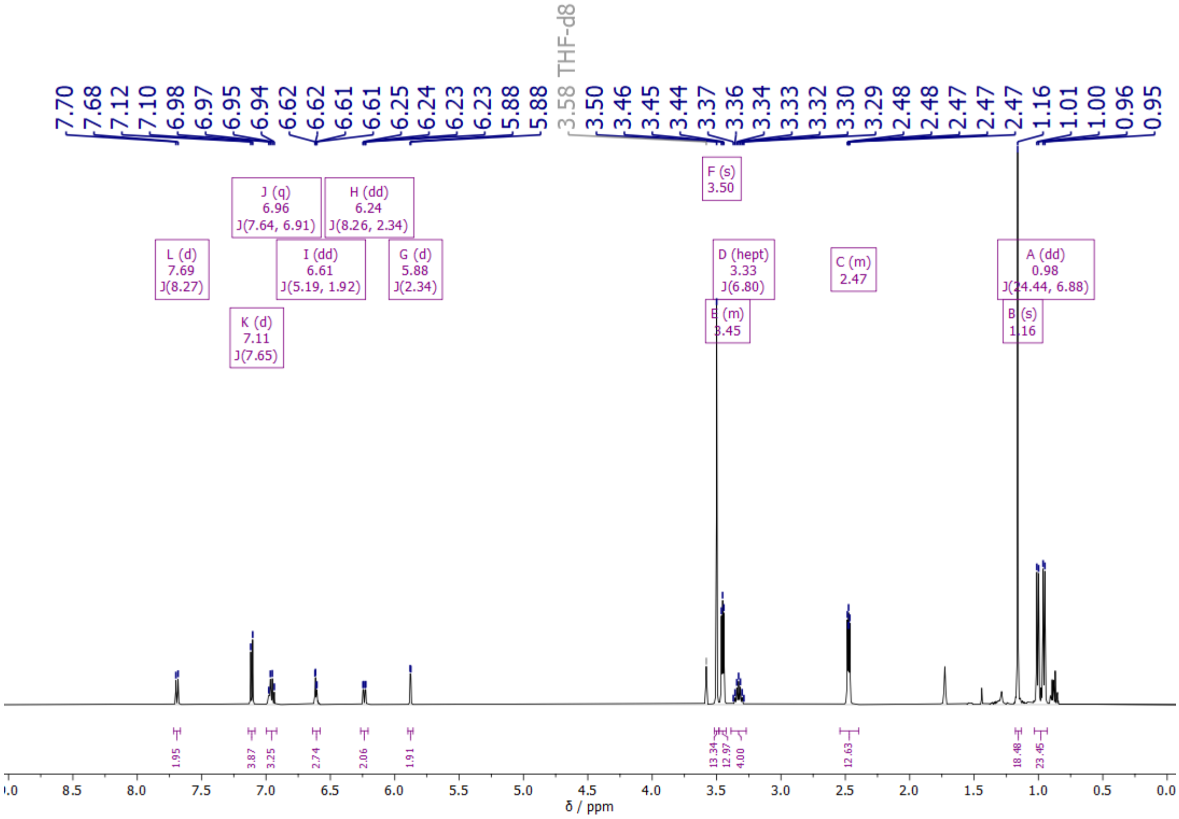

Figure S26. ^{1}H NMR spectrum of isolated **5**, THF-d_8, 25 °C.

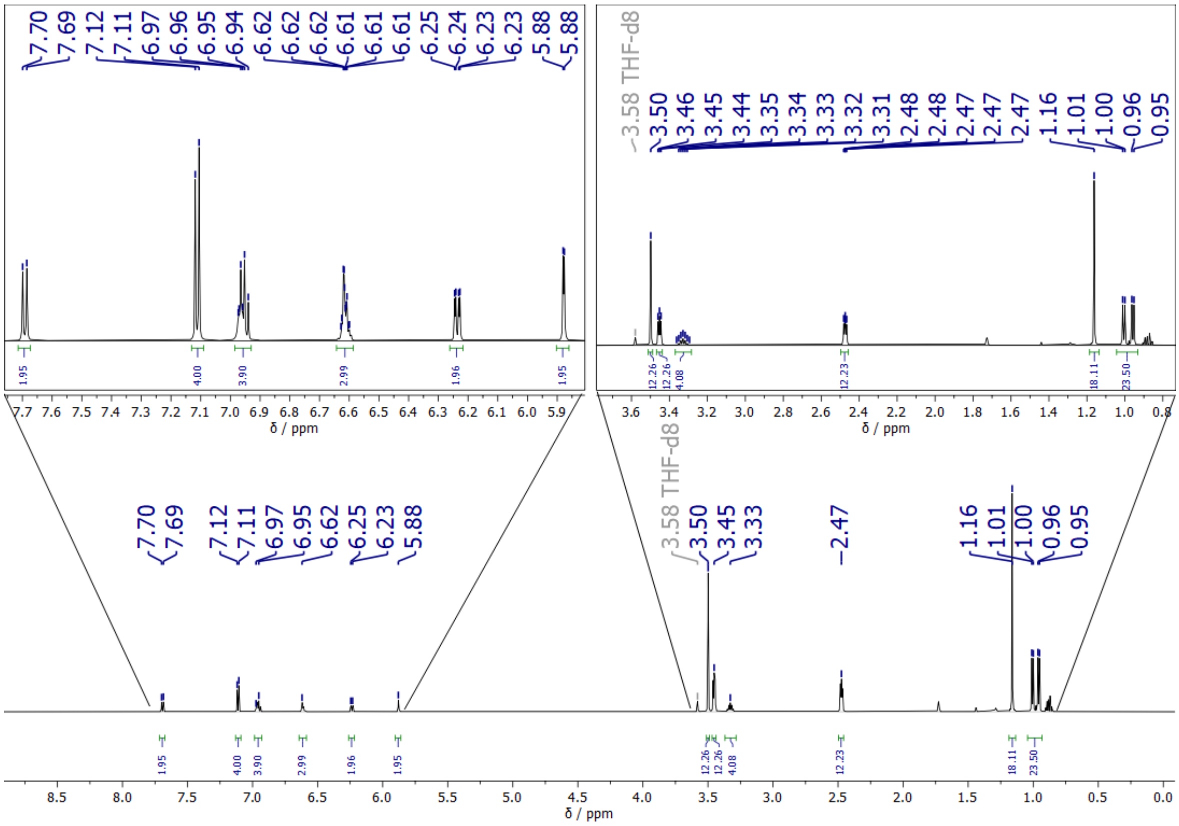

Figure S27. ^{1}H NMR spectrum of isolated **5**, THF-d_8, 25 °C.

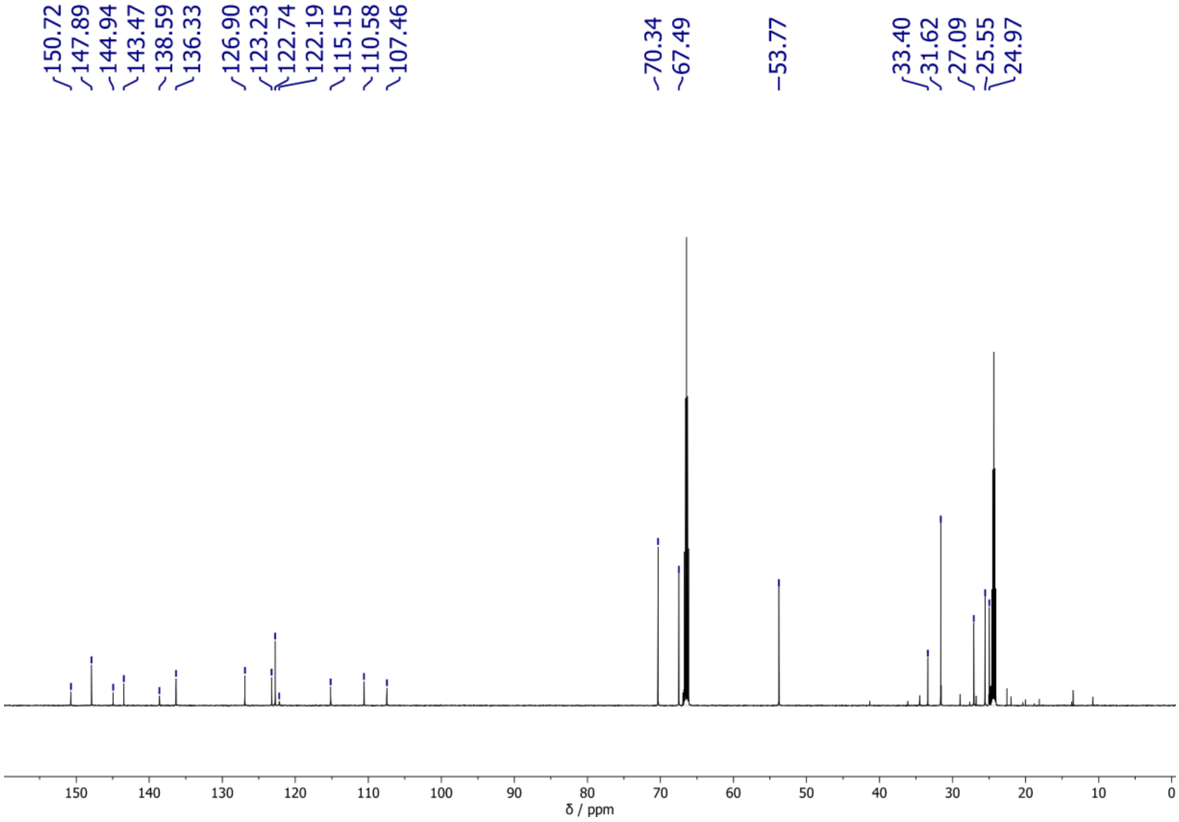

Figure S28. $^{13}C\{^{1}H\}$ NMR spectrum of isolated **5**, THF-d_8, 25 °C.

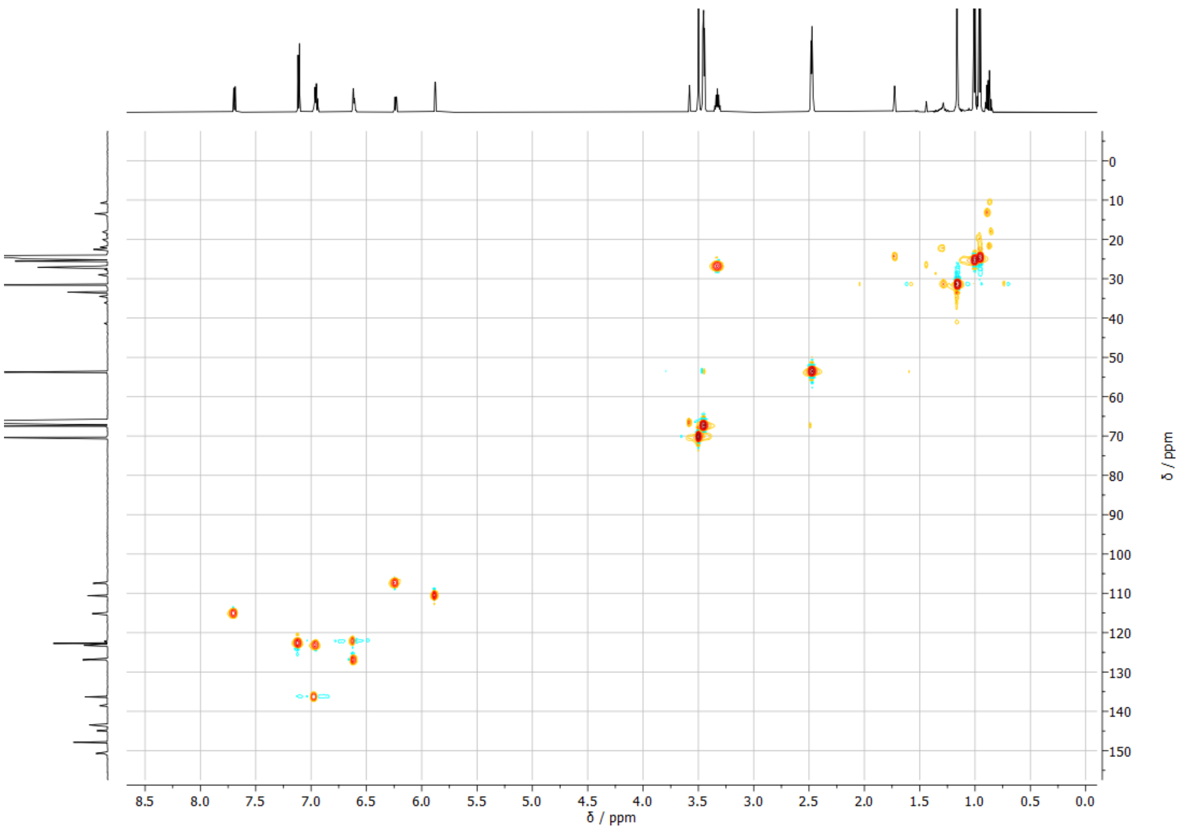

Figure S29. HSQC spectrum of isolated **5**, THF-d_8, 25 °C.

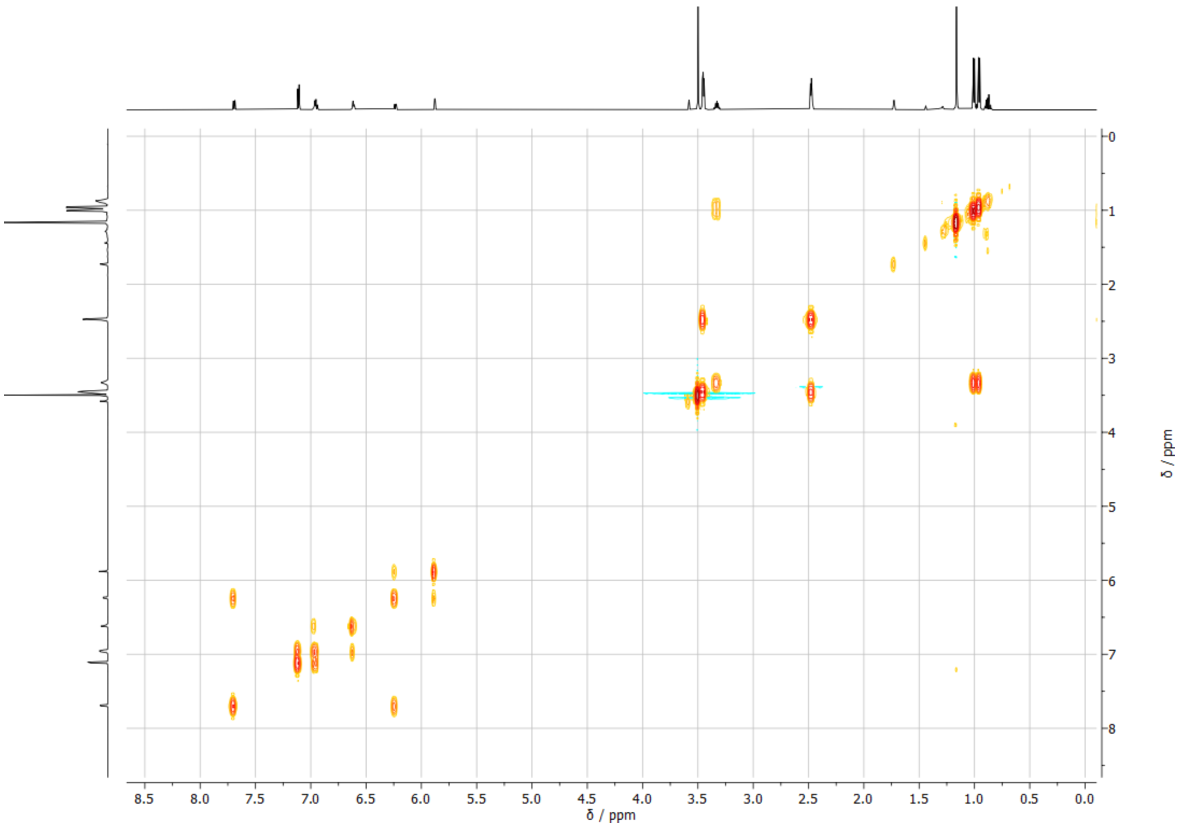

Figure S30. HSQC spectrum of isolated **5**, THF-d_8, 25 °C.

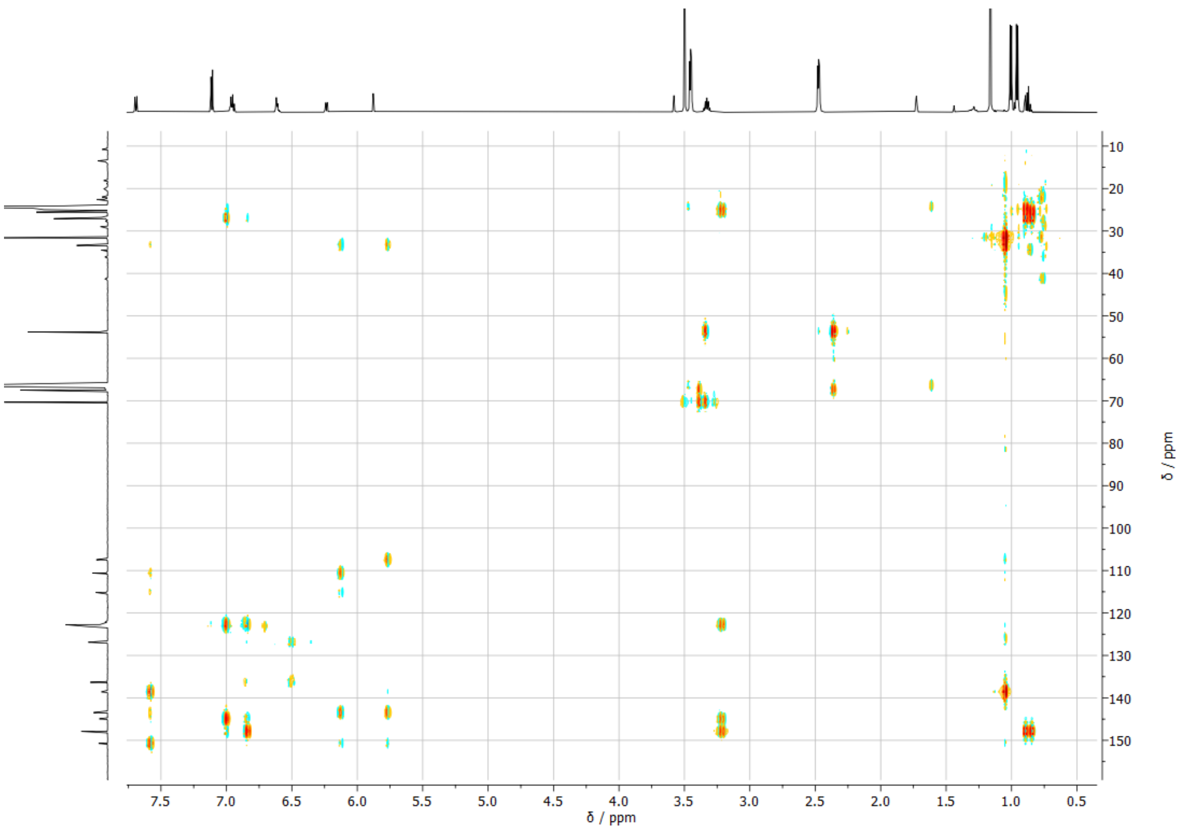

Figure S31. HSQC spectrum of isolated **5**, THF-d8, 25 °C.

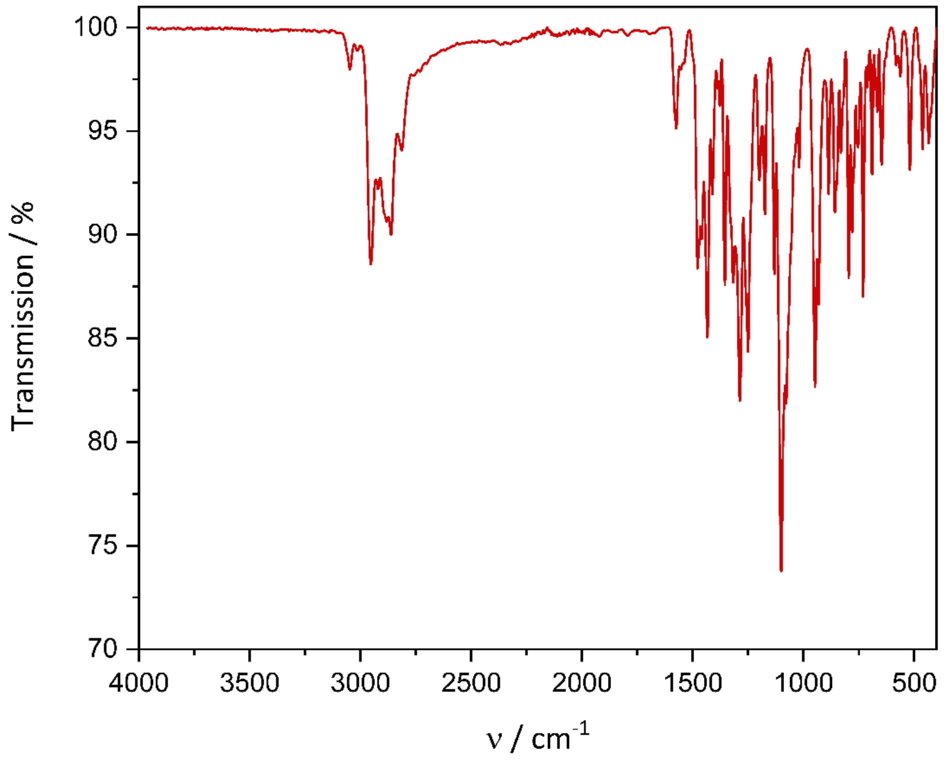

Figure S32. ATR-IR spectrum of **5**, solid, 25 °C.

Single Crystal X-ray Diffraction

Crystallographic Details of 2

A clear dark blue, plate-shaped crystal was mounted on a MiTeGen micromount with perfluoroether oil. Data for **2** were collected from a shock-cooled single crystal at 100.00 K on a Bruker D8 VENTURE dual wavelength Mo/Cu three-circle diffractometer with a microfocus sealed X-ray tube using a mirror optics as monochromator and a Bruker PHOTON III detector. The diffractometer used Mo K_{α} radiation (λ = 0.71073 Å). All data were integrated with SAINT V8.40B and a multi-scan absorption correction using SADABS 2016/2 was applied.[12,13] The structure was solved by direct methods with SHELXT 2018/2 and refined by full-matrix least-squares methods against F^2 using SHELXL-2019/2.[14,15] All hydrogen atoms were refined isotropic on calculated positions using a riding model with their U_{iso} values constrained to 1.5 times the U_{eq} of their pivot atoms for terminal sp^3 carbon atoms and 1.2 times for all other carbon atoms.

Disordered moieties were refined using bond lengths restraints and displacement parameter restraints. One of the tert-butyl groups of the pincer ligand exhibited disorder which was freely refined. A heavily disordered hexane molecule was treated with the SQUEEZE procedure implemented in olex2.[16]

Crystallographic data for the structures reported in this paper have been deposited with the Cambridge Crystallographic Data Centre.[17] CCDC 2467330 contain the supplementary crystallographic data for this paper. These data can be obtained free of charge from The Cambridge Crystallographic Data Centre via www.ccdc.cam.ac.uk/structures. This report was generated using FinalCif.[18]

Table S1. Crystal data and structure refinement for **2**.

CCDC number	2467330
Empirical formula	$C_{50}H_{72}Bi_1N_3$
Formula weight	924.130
Temperature [K]	100.00
Crystal system	triclinic
Space group (number)	$P\bar{1}$ (2)
a [Å]	10.2367(13)
b [Å]	14.0390(16)
c [Å]	17.218(2)
α [°]	102.459(5)
β [°]	92.367(5)
γ [°]	107.869(4)
Volume [Å^3]	2284.1(5)
Z	2
ρ_{calc} [gcm^{-3}]	1.344
μ [mm^{-1}]	3.888
F(000)	945.828
Crystal size [mm^3]	0.02×0.08×0.09
Crystal colour	clear dark blue
Crystal shape	plate
Radiation	Mo K_{α} (λ=0.71073 Å)
2θ range [°]	4.42 to 56.70 (0.75 Å)
Index ranges	$-13 \le h \le 13$<br>$-18 \le k \le 18$<br>$-22 \le l \le 22$
Reflections collected	135079
Independent reflections	11400<br>R_{int} = 0.0738<br>R_{sigma} = 0.0320
Completeness to θ = 25.2417°	99.9 %
Data / Restraints / Parameters	11400 / 6 / 478
Absorption correction T_{min}/T_{max} (method)	0.5849 / 0.7457 (multi-scan)
Goodness-of-fit on F^2	1.0117
Final R indexes [$I \ge 2\sigma(I)$]	R_1 = 0.0218<br>wR_2 = 0.0519
Final R indexes [all data]	R_1 = 0.0252<br>wR_2 = 0.0533
Largest peak/hole [eÅ^{-3}]	0.66/−0.70

Table S2. Bond lengths and angles for **2**.

Atom–Atom	Length [Å]
Bi1–N1	2.2722(19)
Bi1–N2	2.2053(18)
Bi1–N3	2.2736(19)
N1–C1	1.349(3)
N1–C21	1.431(3)
C1–C2	1.418(3)
C1–C6	1.429(3)
N2–C6	1.380(3)
N2–C8	1.383(3)
C2–C3	1.382(3)
N3–C7	1.351(3)
N3–C33	1.430(3)
C3–C4	1.410(3)
C3–C13	1.533(3)
C4–C5	1.374(3)
C5–C6	1.412(3)
C7–C8	1.433(3)
C7–C12	1.418(3)
C8–C9	1.406(3)
C9–C10	1.373(3)
C10–C11	1.413(3)
C11–C12	1.380(3)
C11–C17	1.534(3)
C13–C14	1.540(4)
C13–C15	1.531(4)
C13–C16	1.541(4)
C17–C18	1.617(6)
C17–C19	1.546(5)
C17–C20	1.453(5)
C17–C18A	1.617(6)
C17–C19A	1.420(6)
C17–C20A	1.571(6)
C21–C22	1.408(3)
C21–C26	1.405(3)
C22–C23	1.386(3)
C22–C27	1.522(3)
C23–C24	1.391(4)
C24–C25	1.369(4)
C25–C26	1.406(3)
C26–C30	1.513(3)
C27–C28	1.533(4)
C27–C29	1.515(4)
C30–C31	1.525(4)
C30–C32	1.542(4)
C33–C34	1.404(3)
C33–C38	1.405(3)
C34–C35	1.400(3)
C34–C39	1.521(3)
C35–C36	1.386(4)
C36–C37	1.379(4)
C37–C38	1.400(3)
C38–C42	1.522(3)
C39–C40	1.533(4)
C39–C41	1.530(4)
C42–C43	1.535(4)
C42–C44	1.525(4)

Atom–Atom–Atom	Angle [°]
N2–Bi1–N1	72.02(7)
N3–Bi1–N1	143.79(7)
N3–Bi1–N2	72.04(7)
C1–N1–Bi1	116.19(14)
C21–N1–Bi1	121.95(14)
C21–N1–C1	121.50(19)
C2–C1–N1	124.7(2)
C6–C1–N1	116.47(19)
C6–C1–C2	118.8(2)
C6–N2–Bi1	117.53(14)
C8–N2–Bi1	117.38(14)
C8–N2–C6	124.99(19)
C3–C2–C1	122.4(2)
C7–N3–Bi1	116.54(14)
C33–N3–Bi1	121.04(14)
C33–N3–C7	122.38(19)
C4–C3–C2	117.6(2)
C13–C3–C2	120.6(2)
C13–C3–C4	121.7(2)
C5–C4–C3	121.8(2)
C6–C5–C4	121.2(2)
N2–C6–C1	115.86(19)
C5–C6–C1	117.9(2)
C5–C6–N2	125.7(2)
C8–C7–N3	116.22(19)
C12–C7–N3	125.3(2)
C12–C7–C8	118.4(2)
C7–C8–N2	115.75(19)
C9–C8–N2	125.9(2)
C9–C8–C7	118.2(2)
C10–C9–C8	121.1(2)
C11–C10–C9	121.7(2)
C12–C11–C10	117.8(2)
C17–C11–C10	120.2(2)
C17–C11–C12	121.9(2)
C11–C12–C7	122.2(2)
C14–C13–C3	109.1(2)
C15–C13–C3	111.5(2)
C15–C13–C14	108.5(2)
C16–C13–C3	110.6(2)
C16–C13–C14	109.5(2)
C16–C13–C15	107.5(2)
C18–C17–C11	106.6(3)
C19–C17–C11	112.8(3)
C19–C17–C18	103.7(3)
C20–C17–C11	111.9(3)
C20–C17–C18	109.8(3)
C20–C17–C19	111.4(3)
C18A–C17–C11	106.3(3)
C18A–C17–C18	147.0(3)
C18A–C17–C19	61.2(3)

C18A–C17–C20	58.1(3)	C29–C27–C28	111.2(3)
C19A–C17–C11	111.6(3)	C31–C30–C26	111.5(2)
C19A–C17–C18	53.5(4)	C32–C30–C26	111.7(2)
C19A–C17–C19	52.0(4)	C32–C30–C31	110.7(2)
C19A–C17–C20	136.3(4)	C34–C33–N3	120.2(2)
C19A–C17–C18A	111.3(4)	C38–C33–N3	118.2(2)
C20A–C17–C11	112.9(3)	C38–C33–C34	121.5(2)
C20A–C17–C18	66.4(3)	C35–C34–C33	118.2(2)
C20A–C17–C19	134.1(3)	C39–C34–C33	121.4(2)
C20A–C17–C20	45.3(3)	C39–C34–C35	120.4(2)
C20A–C17–C18A	101.8(4)	C36–C35–C34	120.8(2)
C20A–C17–C19A	112.3(4)	C37–C36–C35	120.4(2)
C22–C21–N1	118.7(2)	C38–C37–C36	120.9(2)
C26–C21–N1	120.0(2)	C37–C38–C33	118.2(2)
C26–C21–C22	121.2(2)	C42–C38–C33	121.9(2)
C23–C22–C21	118.5(2)	C42–C38–C37	119.9(2)
C27–C22–C21	121.6(2)	C40–C39–C34	110.7(2)
C27–C22–C23	119.9(2)	C41–C39–C34	112.7(2)
C24–C23–C22	120.9(2)	C41–C39–C40	110.7(2)
C25–C24–C23	120.2(2)	C43–C42–C38	111.5(2)
C26–C25–C24	121.2(2)	C44–C42–C38	110.9(2)
C25–C26–C21	117.8(2)	C44–C42–C43	111.5(2)
C30–C26–C21	121.7(2)		
C30–C26–C25	120.5(2)		
C28–C27–C22	110.6(2)		
C29–C27–C22	111.6(2)		

Bonds and angles to hydrogen atoms were omitted.

Crystallographic Details of 3

A clear dark turquoise, needle-shaped crystal was mounted on a MiTeGen micromount with perfluoroether oil. Data for **3** were collected from a shock-cooled single crystal at 100(2) K on a Bruker D8 VENTURE dual wavelength Mo/Cu three-circle diffractometer with a microfocus sealed X-ray tube using a mirror optics as monochromator and a Bruker PHOTON III detector. The diffractometer was equipped with an Oxford Cryostream 800 low temperature device and used MoK_α radiation (λ = 0.71073 Å). All data were integrated with SAINT V8.40B and a multi-scan absorption correction using SADABS 2016/2 was applied.[12,13] The structure was solved by direct methods with SHELXT 2018/2 and refined by full-matrix least-squares methods against F^2 using SHELXL-2019/2.[14,15] All non-hydrogen atoms were refined with anisotropic displacement parameters. All hydrogen atoms were refined isotropic on calculated positions using a riding model with their U_{iso} values constrained to 1.5 times the U_{eq} of their pivot atoms for terminal sp^3 carbon atoms and 1.2 times for all other carbon atoms.

Disordered moieties were refined using bond lengths restraints and displacement parameter restraints. A tert-butyl and isopropyl group of the NNN pincer ligand exhibited disorder and were freely refined. Once molecule of THF co-crystallized and exhibits positional disorder which was freely refined.

Crystallographic data for the structures reported in this paper have been deposited with the Cambridge Crystallographic Data Centre.[17] CCDC 2467405 contain the supplementary crystallographic data for this paper. These data can be obtained free of charge from The Cambridge Crystallographic Data Centre via www.ccdc.cam.ac.uk/structures. This report was generated using FinalCif.[18]

Table S3. Crystal data and structure refinement for **3**.

CCDC number	2467405
Empirical formula	$C_{66}H_{102}BiKN_5O_7$
Formula weight	1325.60
Temperature [K]	100(2)
Crystal system	monoclinic
Space group (number)	$P2_1/n$ (14)
a [Å]	14.1625(8)
b [Å]	24.3411(13)
c [Å]	21.0188(13)
α [°]	90
β [°]	93.179(2)
γ [°]	90
Volume [Å^3]	7234.7(7)
Z	4
ρ_{calc} [gcm^{-3}]	1.217
μ [mm^{-1}]	2.543
F(000)	2764
Crystal size [mm^3]	0.040×0.670×0.900
Crystal colour	clear dark turquoise
Crystal shape	needle
Radiation	MoK_α (λ=0.71073 Å)
2θ range [°]	3.87 to 56.81 (0.75 Å)
Index ranges	−18 ≤ h ≤ 18<br>−32 ≤ k ≤ 32<br>−28 ≤ l ≤ 28
Reflections collected	132961
Independent reflections	17976<br>R_{int} = 0.0692<br>R_{sigma} = 0.0446
Completeness to θ = 25.242°	99.8 %
Data / Restraints / Parameters	17976 / 53 / 723
Absorption correction T_{min}/T_{max} (method)	0.4283 / 0.7457 (multi-scan)
Goodness-of-fit on F^2	1.058
Final R indexes [I≥2σ(I)]	R_1 = 0.0658<br>wR_2 = 0.1629
Final R indexes [all data]	R_1 = 0.0954<br>wR_2 = 0.1816
Largest peak/hole [eÅ^{-3}]	2.22/−2.75

Table S4. Bond lengths and angles for **3**.

**Atom–Atom**	**Length [Å]**
C20–C17	1.524(9)
C27–C28	1.463(17)
C27–C29A	1.47(3)
C27–C22	1.513(10)
C27–C29	1.518(18)
C27–C28A	1.66(2)
Bi1–N2	2.224(5)
Bi1–N3	2.303(5)
Bi1–N1	2.315(5)
K1–O3	2.791(7)
K1–O6	2.808(6)
K1–O4	2.830(6)
K1–O2	2.831(7)
K1–O5	2.839(7)
K1–O1	2.915(6)
K1–N5	3.009(7)
K1–N4	3.062(8)
K1–C53	3.490(13)
O1–C46	1.348(11)
O1–C47	1.356(14)
O2–C48	1.383(12)
O2–C49	1.422(11)
O3–C53	1.275(12)
O3–C52	1.444(10)
O4–C55	1.346(11)
O4–C54	1.465(9)
O5–C59	1.436(10)
O5–C58	1.447(12)
O6–C61	1.420(11)
O6–C60	1.433(10)
N1–C2	1.367(7)
N1–C21	1.422(7)
N2–C7	1.393(7)
N2–C1	1.407(6)
N3–C12	1.362(7)
N3–C33	1.416(7)
N4–C57	1.435(13)
N4–C51	1.442(12)
N4–C45	1.485(14)
N5–C50	1.426(12)
N5–C56	1.493(12)
N5–C62	1.496(12)
C1–C6	1.396(7)
C1–C2	1.434(7)
C2–C3	1.408(8)
C3–C4	1.383(8)
C4–C5	1.401(8)
C4–C13	1.533(8)
C5–C6	1.385(8)
C7–C8	1.397(7)
C7–C12	1.430(8)
C8–C9	1.393(7)
C9–C10	1.384(8)
C10–C11	1.393(8)
C10–C17	1.537(7)
C11–C12	1.399(8)
C13–C16	1.440(15)
C13–C15A	1.454(18)
C13–C14A	1.540(15)
C13–C15	1.543(12)
C13–C16A	1.593(19)
C13–C14	1.602(15)
C17–C18	1.535(8)
C17–C19	1.541(8)
C21–C26	1.396(9)
C21–C22	1.397(10)
C22–C23	1.402(9)
C23–C24	1.367(11)
C24–C25	1.371(11)
C25–C26	1.399(9)
C26–C30	1.512(10)
C30–C32	1.524(12)
C30–C31	1.528(11)
C33–C38	1.399(10)
C33–C34	1.405(10)
C34–C35	1.406(9)
C34–C39	1.520(11)
C35–C36	1.387(13)
C36–C37	1.351(13)
C37–C38	1.396(10)
C38–C42	1.513(12)
C39–C40	1.506(13)
C39–C41	1.520(12)
C42–C44	1.525(13)
C42–C43	1.564(14)
C45–C46	1.464(15)
C47–C48	1.506(16)
C49–C50	1.551(15)
C51–C52	1.525(14)
C53–C54	1.444(14)
C55–C56	1.518(14)
C57–C58	1.518(15)
C59–C60	1.480(13)
C61–C62	1.479(14)
O7–C63	1.399(16)
O7–C66	1.445(16)
C63–C64	1.382(18)
C64–C65	1.58(2)
C65–C66	1.395(18)
O8–C67	1.434(18)
O8–C70	1.441(18)
C67–C68	1.39(2)
C68–C69	1.57(3)
C69–C70	1.39(2)
O8A–C70A	1.424(19)
O8A–C67A	1.428(19)
C67A–C68A	1.39(2)
C68A–C69A	1.59(3)
C69A–C70A	1.39(2)

Atom–Atom–Atom	Angle [°]
C28–C27–C22	110.4(9)
C29A–C27–C22	115.6(11)
C28–C27–C29	112.6(11)
C22–C27–C29	111.2(8)
C29A–C27–C28A	102.5(14)
C22–C27–C28A	112.4(10)
N2–Bi1–N3	71.67(17)
N2–Bi1–N1	71.90(16)
N3–Bi1–N1	143.42(17)
O3–K1–O6	129.9(2)
O3–K1–O4	60.08(19)
O6–K1–O4	94.78(18)
O3–K1–O2	120.21(19)
O6–K1–O2	103.73(18)
O4–K1–O2	95.95(19)
O3–K1–O5	92.56(19)
O6–K1–O5	60.72(18)
O4–K1–O5	118.89(19)
O2–K1–O5	141.68(19)
O3–K1–O1	96.49(18)
O6–K1–O1	128.07(19)
O4–K1–O1	132.60(18)
O2–K1–O1	58.75(18)
O5–K1–O1	101.36(18)
O3–K1–N5	120.0(2)
O6–K1–N5	61.0(2)
O4–K1–N5	60.3(2)
O2–K1–N5	61.16(19)
O5–K1–N5	121.3(2)
O1–K1–N5	119.4(2)
O3–K1–N4	60.8(2)
O6–K1–N4	119.9(2)
O4–K1–N4	120.6(2)
O2–K1–N4	117.2(2)
O5–K1–N4	59.76(19)
O1–K1–N4	59.0(2)
N5–K1–N4	178.3(2)
O3–K1–C53	19.7(2)
O6–K1–C53	116.8(2)
O4–K1–C53	41.3(2)
O2–K1–C53	119.7(3)
O5–K1–C53	97.9(3)
O1–K1–C53	113.6(2)
N5–K1–C53	101.6(2)
N4–K1–C53	79.3(2)
C46–O1–C47	113.3(8)
C46–O1–K1	117.4(6)
C47–O1–K1	113.2(6)
C48–O2–C49	109.9(8)
C48–O2–K1	117.7(7)
C49–O2–K1	112.7(6)
C53–O3–C52	115.4(9)
C53–O3–K1	112.9(6)
C52–O3–K1	118.0(5)
C55–O4–C54	114.5(7)
C55–O4–K1	113.7(6)
C54–O4–K1	115.0(4)
C59–O5–C58	112.7(7)
C59–O5–K1	114.1(5)
C58–O5–K1	113.1(6)
C61–O6–C60	110.9(7)
C61–O6–K1	118.2(5)
C60–O6–K1	112.1(5)
C2–N1–C21	121.3(5)
C2–N1–Bi1	114.8(4)
C21–N1–Bi1	118.8(4)
C7–N2–C1	124.9(5)
C7–N2–Bi1	117.5(3)
C1–N2–Bi1	117.4(3)
C12–N3–C33	121.5(5)
C12–N3–Bi1	115.8(4)
C33–N3–Bi1	121.9(4)
C57–N4–C51	110.4(10)
C57–N4–C45	112.0(10)
C51–N4–C45	110.6(8)
C57–N4–K1	108.7(6)
C51–N4–K1	107.3(6)
C45–N4–K1	107.7(6)
C50–N5–C56	107.7(9)
C50–N5–C62	111.1(8)
C56–N5–C62	111.6(8)
C50–N5–K1	110.7(6)
C56–N5–K1	110.2(5)
C62–N5–K1	105.6(5)
C6–C1–N2	126.7(5)
C6–C1–C2	117.6(5)
N2–C1–C2	115.5(5)
N1–C2–C3	124.0(5)
N1–C2–C1	117.3(5)
C3–C2–C1	118.7(5)
C4–C3–C2	122.9(5)
C3–C4–C5	117.6(5)
C3–C4–C13	121.4(5)
C5–C4–C13	121.0(5)
C6–C5–C4	121.1(5)
C5–C6–C1	122.0(5)
N2–C7–C8	126.3(5)
N2–C7–C12	115.7(5)
C8–C7–C12	117.9(5)
C9–C8–C7	121.2(5)
C10–C9–C8	121.3(5)
C9–C10–C11	118.1(5)
C9–C10–C17	123.0(5)
C11–C10–C17	118.9(5)
C10–C11–C12	122.3(5)
N3–C12–C11	124.3(5)
N3–C12–C7	116.9(5)
C11–C12–C7	118.9(5)
C16–C13–C4	111.3(7)
C15A–C13–C4	110.6(9)
C15A–C13–C14A	112.6(11)
C4–C13–C14A	112.6(7)

C16–C13–C15	112.2(9)	C40–C39–C41	111.2(7)
C4–C13–C15	113.8(6)	C34–C39–C41	111.6(7)
C15A–C13–C16A	109.9(12)	C38–C42–C44	112.3(8)
C4–C13–C16A	106.6(8)	C38–C42–C43	110.0(8)
C14A–C13–C16A	104.1(10)	C44–C42–C43	112.7(8)
C16–C13–C14	107.2(10)	C46–C45–N4	115.6(8)
C4–C13–C14	108.3(7)	O1–C46–C45	110.8(8)
C15–C13–C14	103.4(9)	O1–C47–C48	112.9(11)
C20–C17–C18	107.8(5)	O2–C48–C47	109.3(9)
C20–C17–C10	112.1(5)	O2–C49–C50	110.5(8)
C18–C17–C10	110.1(5)	N5–C50–C49	113.0(9)
C20–C17–C19	108.7(5)	N4–C51–C52	115.4(8)
C18–C17–C19	109.3(5)	O3–C52–C51	108.3(7)
C10–C17–C19	108.8(5)	O3–C53–C54	121.2(12)
C26–C21–C22	120.5(5)	O3–C53–K1	47.5(5)
C26–C21–N1	119.9(6)	C54–C53–K1	86.6(7)
C22–C21–N1	119.4(6)	C53–C54–O4	105.5(7)
C21–C22–C23	118.1(6)	O4–C55–C56	112.9(8)
C21–C22–C27	121.1(6)	N5–C56–C55	109.1(8)
C23–C22–C27	120.8(7)	N4–C57–C58	114.2(10)
C24–C23–C22	121.9(7)	O5–C58–C57	106.0(10)
C23–C24–C25	119.5(6)	O5–C59–C60	110.1(7)
C24–C25–C26	121.1(7)	O6–C60–C59	108.8(7)
C21–C26–C25	118.9(7)	O6–C61–C62	107.5(8)
C21–C26–C30	122.6(6)	C61–C62–N5	115.2(8)
C25–C26–C30	118.5(6)	C63–O7–C66	106.6(14)
C26–C30–C32	114.0(7)	C64–C63–O7	104.9(14)
C26–C30–C31	109.8(7)	C63–C64–C65	108.5(15)
C32–C30–C31	110.8(7)	C66–C65–C64	100.2(14)
C38–C33–C34	119.2(6)	C65–C66–O7	110.7(15)
C38–C33–N3	119.9(6)	C67–O8–C70	100.2(18)
C34–C33–N3	120.7(6)	C68–C67–O8	108.7(19)
C33–C34–C35	118.9(7)	C67–C68–C69	102.2(17)
C33–C34–C39	121.9(6)	C70–C69–C68	104.0(18)
C35–C34–C39	119.2(7)	C69–C70–O8	109.6(18)
C36–C35–C34	120.5(8)	C70A–O8A–C67A	101.2(19)
C37–C36–C35	120.4(7)	C68A–C67A–O8A	105.8(19)
C36–C37–C38	120.8(8)	C67A–C68A–C69A	104.5(18)
C37–C38–C33	120.1(7)	C70A–C69A–C68A	100.9(18)
C37–C38–C42	119.9(7)	C69A–C70A–O8A	108(2)
C33–C38–C42	120.0(6)		
C40–C39–C34	111.0(7)		

Bonds and angles to hydrogen atoms were omitted.

Crystallographic Details of [K@222-crypt][Ph*O]

A colourless, block-shaped crystal was mounted on a MiTeGen micromount with perfluoroether oil. Data for [K@222-crypt][Ph*O] were collected from a shock-cooled single crystal at 100(2) K on a Bruker D8 VENTURE dual wavelength Mo/Cu three-circle diffractometer with a microfocus sealed X-ray tube using a mirror optics as monochromator and a Bruker PHOTON III detector. The diffractometer was equipped with an Oxford Cryostream 800 low temperature device and used MoK_α radiation (λ = 0.71073 Å). All data were integrated with SAINT V8.40B and a multi-scan absorption correction using SADABS 2016/2 was applied.[12,13] The structure was solved by direct methods with SHELXT 2018/2 and refined by full-matrix least-squares methods against F^2 using SHELXL-2019/2.[14,15] All non-hydrogen atoms were refined with anisotropic displacement parameters. All hydrogen atoms were refined isotropic on calculated positions using a riding model with their U_{iso} values constrained to 1.5 times the U_{eq} of their pivot atoms for terminal sp^3 carbon atoms and 1.2 times for all other carbon atoms.

Disordered moieties were refined using bond lengths restraints and displacement parameter restraints. The complex co-crystallizes with tetrahydrofuran, which displayed positional disorder and was freely refined.

Crystallographic data for the structures reported in this paper have been deposited with the Cambridge Crystallographic Data Centre.[17] CCDC 2467406 contain the supplementary crystallographic data for this paper. These data can be obtained free of charge from The Cambridge Crystallographic Data Centre via www.ccdc.cam.ac.uk/structures. This report was generated using FinalCif.[18]

Table S5. Crystal data and structure refinement for [K@222-crypt][Ph*O].

CCDC number	2467406
Empirical formula	$C_{44}H_{81}KN_2O_9$
Formula weight	821.20
Temperature [K]	100(2)
Crystal system	orthorhombic
Space group (number)	$P2_12_12_1$ (19)
a [Å]	10.5722(2)
b [Å]	20.5736(5)
c [Å]	21.7074(5)
α [°]	90
β [°]	90
γ [°]	90
Volume [Å^3]	4721.54(18)
Z	4
ρ_{calc} [gcm^{-3}]	1.155
μ [mm^{-1}]	0.164
F(000)	1800
Crystal size [mm^3]	0.160×0.160×0.240
Crystal colour	colourless
Crystal shape	block
Radiation	MoK_α (λ=0.71073 Å)
2θ range [°]	3.96 to 50.75 (0.83 Å)
Index ranges	−12 ≤ h ≤ 12<br>−24 ≤ k ≤ 24<br>−26 ≤ l ≤ 26
Reflections collected	57949
Independent reflections	8660<br>R_{int} = 0.0529<br>R_{sigma} = 0.0306
Completeness to θ = 25.242°	99.9 %
Data / Restraints / Parameters	8660 / 37 / 507
Absorption correction T_{min}/T_{max} (method)	0.6944 / 0.7452 (multi-scan)
Goodness-of-fit on F^2	0.989
Final *R* indexes [I≥2σ(I)]	R_1 = 0.0318<br>wR_2 = 0.0821
Final *R* indexes [all data]	R_1 = 0.0341<br>wR_2 = 0.0840
Largest peak/hole [eÅ^{-3}]	0.41/−0.26
Flack X parameter	0.027(11)

Table S6. Bond lengths and angles for [K@222-crypt][Ph*O].

**Atom–Atom**	**Length [Å]**
K1–O7	2.7835(16)
K1–O3	2.8018(17)
K1–O5	2.8074(17)
K1–O6	2.8344(17)
K1–O2	2.8459(17)
K1–O4	2.8528(17)
K1–N1	3.000(2)
K1–N2	3.013(2)
O1–C1	1.293(3)
O2–C21	1.418(3)
O2–C20	1.425(3)
O3–C22	1.423(3)
O3–C23	1.426(3)
O4–C27	1.418(3)
O4–C26	1.424(3)
O5–C28	1.420(3)
O5–C29	1.426(3)
O6–C32	1.421(3)
O6–C33	1.431(3)
O7–C34	1.419(3)
O7–C35	1.435(3)
N1–C31	1.469(3)
N1–C19	1.476(3)
N1–C25	1.483(3)
N2–C24	1.468(3)
N2–C30	1.470(3)
N2–C36	1.471(3)
C1–C2	1.442(3)
C1–C6	1.444(3)
C2–C3	1.396(3)
C2–C7	1.533(3)
C3–C4	1.391(3)
C4–C5	1.397(3)
C4–C11	1.540(3)
C5–C6	1.392(3)
C6–C15	1.540(3)
C7–C10	1.536(3)
C7–C9	1.538(3)
C7–C8	1.544(3)
C11–C12	1.528(3)
C11–C13	1.532(3)
C11–C14	1.536(3)
C15–C17	1.531(3)
C15–C16	1.539(3)
C15–C18	1.542(3)
C19–C20	1.507(4)
C21–C22	1.492(4)
C23–C24	1.512(4)
C25–C26	1.502(4)
C27–C28	1.496(4)
C29–C30	1.503(4)
C31–C32	1.508(4)
C33–C34	1.486(4)
C35–C36	1.499(3)
O8–C40	1.417(15)
O8–C37	1.422(9)
C37–C38	1.519(6)
C38–C39	1.484(7)
C39–C40	1.561(9)
O8A–C40A	1.422(19)
O8A–C37A	1.427(17)
C37A–C38A	1.496(10)
C38A–C39A	1.456(13)
C39A–C40A	1.472(15)
O9–C44	1.473(10)
O9–C41	1.421(7)
C41–C42	1.513(6)
C42–C43	1.528(5)
C43–C44	1.463(6)
O9A–C44A	1.458(18)
O9A–C41A	1.43(2)
C41A–C42A	1.478(18)
C42A–C43A	1.491(18)
C43A–C44A	1.448(18)

**Atom–Atom–Atom**	**Angle [°]**
O7–K1–O3	93.20(5)
O7–K1–O5	95.39(5)
O3–K1–O5	103.52(5)
O7–K1–O6	60.75(5)
O3–K1–O6	115.66(6)
O5–K1–O6	134.01(5)
O7–K1–O2	131.96(5)
O3–K1–O2	59.75(5)
O5–K1–O2	127.45(5)
O6–K1–O2	94.01(5)
O7–K1–O4	117.51(5)
O3–K1–O4	145.09(5)
O5–K1–O4	59.99(5)
O6–K1–O4	94.83(5)
O2–K1–O4	103.82(5)
O7–K1–N1	119.66(6)
O3–K1–N1	119.90(6)
O5–K1–N1	119.68(6)
O6–K1–N1	59.61(6)
O2–K1–N1	61.02(5)
O4–K1–N1	60.44(6)
O7–K1–N2	60.09(5)
O3–K1–N2	60.48(5)
O5–K1–N2	60.17(5)
O6–K1–N2	120.19(5)
O2–K1–N2	119.37(5)
O4–K1–N2	119.34(5)
N1–K1–N2	179.61(6)
C21–O2–C20	111.7(2)
C21–O2–K1	112.22(14)
C20–O2–K1	117.67(15)
C22–O3–C23	110.69(18)
C22–O3–K1	117.48(13)

C23–O3–K1	116.95(14)
C27–O4–C26	111.35(19)
C27–O4–K1	114.03(14)
C26–O4–K1	114.50(15)
C28–O5–C29	111.3(2)
C28–O5–K1	115.02(14)
C29–O5–K1	120.86(14)
C32–O6–C33	111.7(2)
C32–O6–K1	118.32(15)
C33–O6–K1	112.59(14)
C34–O7–C35	110.87(18)
C34–O7–K1	114.69(14)
C35–O7–K1	120.02(13)
C31–N1–C19	110.5(2)
C31–N1–C25	108.6(2)
C19–N1–C25	108.5(2)
C31–N1–K1	111.20(15)
C19–N1–K1	107.11(15)
C25–N1–K1	110.91(16)
C24–N2–C30	109.66(19)
C24–N2–C36	109.81(18)
C30–N2–C36	110.8(2)
C24–N2–K1	109.85(14)
C30–N2–K1	108.02(13)
C36–N2–K1	108.64(13)
O1–C1–C2	121.6(2)
O1–C1–C6	121.69(19)
C2–C1–C6	116.67(19)
C3–C2–C1	120.0(2)
C3–C2–C7	120.55(19)
C1–C2–C7	119.48(19)
C4–C3–C2	123.3(2)
C3–C4–C5	116.8(2)
C3–C4–C11	122.9(2)
C5–C4–C11	120.2(2)
C6–C5–C4	123.3(2)
C5–C6–C1	119.91(19)
C5–C6–C15	120.83(19)
C1–C6–C15	119.25(19)
C2–C7–C10	112.90(18)
C2–C7–C9	110.05(18)
C10–C7–C9	107.17(18)
C2–C7–C8	110.14(18)
C10–C7–C8	107.13(18)
C9–C7–C8	109.33(18)
C12–C11–C13	107.5(2)
C12–C11–C14	107.6(2)
C13–C11–C14	108.8(2)
C12–C11–C4	112.5(2)

C13–C11–C4	109.65(18)
C14–C11–C4	110.68(19)
C17–C15–C16	106.68(19)
C17–C15–C6	113.00(19)
C16–C15–C6	109.90(18)
C17–C15–C18	107.25(18)
C16–C15–C18	109.47(18)
C6–C15–C18	110.40(18)
N1–C19–C20	114.2(2)
O2–C20–C19	109.4(2)
O2–C21–C22	109.2(2)
O3–C22–C21	109.1(2)
O3–C23–C24	109.15(19)
N2–C24–C23	113.89(19)
N1–C25–C26	113.2(2)
O4–C26–C25	110.1(2)
O4–C27–C28	109.8(2)
O5–C28–C27	108.7(2)
O5–C29–C30	109.4(2)
N2–C30–C29	114.2(2)
N1–C31–C32	113.1(2)
O6–C32–C31	109.1(2)
O6–C33–C34	109.5(2)
O7–C34–C33	109.0(2)
O7–C35–C36	108.45(19)
N2–C36–C35	113.3(2)
C40–O8–C37	106.2(9)
O8–C37–C38	106.4(10)
C39–C38–C37	105.5(4)
C38–C39–C40	101.1(5)
O8–C40–C39	102.0(19)
C40A–O8A–C37A	108.4(15)
O8A–C37A–C38A	105(2)
C39A–C38A–C37A	102.8(8)
C38A–C39A–C40A	107.5(10)
O8A–C40A–C39A	106.6(15)
C44–O9–C41	108.2(5)
O9–C41–C42	106.9(5)
C41–C42–C43	102.3(3)
C44–C43–C42	102.0(3)
O9–C44–C43	106.3(4)
C44A–O9A–C41A	104.1(17)
O9A–C41A–C42A	108.7(16)
C41A–C42A–C43A	105.1(14)
C44A–C43A–C42A	101.2(13)
O9A–C44A–C43A	111.7(15)

Bonds and angles to hydrogen atoms were omitted.

Crystallographic Details of 5

A dark red crystal was mounted on a MiTeGen micromount with perfluoroether oil. Data for **5** were collected from a shock-cooled single crystal at 101(2) K on a Bruker D8 VENTURE dual wavelength Mo/Cu three-circle diffractometer with a microfocus sealed X-ray tube using a mirror optics as monochromator and a Bruker PHOTON III detector. The diffractometer was equipped with an Oxford Cryostream 800 low temperature device and used MoK_α radiation (λ = 0.71073 Å). All data were integrated with SAINT V8.40B and a multi-scan absorption correction using SADABS 2016/2 was applied.[12,13] The structure was solved by direct methods with SHELXT 2018/2 and refined by full-matrix least-squares methods against F^2 using SHELXL-2019/2.[14,15] All non-hydrogen atoms were refined with isotropic displacement parameters. All hydrogen atoms were refined isotropic on calculated positions using a riding model with their U_{iso} values constrained to 1.5 times the U_{eq} of their pivot atoms for terminal sp^3 carbon atoms and 1.2 times for all other carbon atoms.

*Single crystals of compound **5** exhibit extreme sensitivity to oxygen and moisture leading to rapid decomposition. This was evident by an observed color change from dark red to black and a loss of crystallinity. After numerous attempts, we successfully collected data that confirmed the proposed structure. However, the low-quality dataset could only be refined in the space group $P1$. Due to the low quality of the data, we present this dataset merely as proof of connectivity between atoms and refrain from discussing bond lengths and angles. Nevertheless, the obtained structure is in line with our NMR spectroscopic and combustion analysis results.*

Crystallographic data for the structures reported in this paper have **not** been deposited with the Cambridge Crystallographic Data Centre.[17] This report was generated using FinalCif.[18]

Table S7. Crystal data and structure refinement for **5**.

CCDC number	Not submitted
Empirical formula	$C_{136}H_{197}Bi_2K_2N_{10}O_{12}Se_2$
Formula weight	2818.10
Temperature [K]	101(2)
Crystal system	triclinic
Space group (number)	$P1$ (1)
a [Å]	14.805(2)
b [Å]	14.830(2)
c [Å]	18.843(3)
α [°]	81.872(5)
β [°]	83.377(4)
γ [°]	89.728(4)
Volume [Å^3]	4068.1(10)
Z	1
ρ_{calc} [gcm^{-3}]	1.150
μ [mm^{-1}]	2.709
F(000)	1451
Crystal size [mm^3]	0.020×0.170×0.180
Crystal colour	Dark red
Crystal shape	block
Radiation	MoK_α (λ=0.71073 Å)
2θ range [°]	3.90 to 41.96 (0.99 Å)
Index ranges	−14 ≤ h ≤ 14<br>−14 ≤ k ≤ 14<br>−18 ≤ l ≤ 18
Reflections collected	41621
Independent reflections	15147<br>R_{int} = 0.0489<br>R_{sigma} = 0.0684
Completeness to θ = 20.978°	98.4 %
Data / Restraints / Parameters	15147 / 3 / 684
Absorption correction T_{min}/T_{max} (method)	Multi-scan (multi-scan)
Goodness-of-fit on F^2	1.080
Final R indexes [I≥2σ(I)]	R_1 = 0.0930<br>wR_2 = 0.2365
Final R indexes [all data]	R_1 = 0.1055<br>wR_2 = 0.2493
Largest peak/hole [eÅ^{-3}]	6.45/−6.50
Flack X parameter	0.077(4)

Table S8. Bond lengths and angles for **5**.

Atom–Atom	Length [Å]
Bi1_1–N3_1	2.21(3)
Bi1_1–N1_1	2.22(2)
Bi1_1–N2_1	2.24(2)
Bi1_1–Se1_1	2.873(4)
Se1_1–C45_1	1.93(3)
N1_1–C2_1	1.41(4)
N1_1–C21_1	1.43(4)
C1_1–C6_1	1.35(4)
C1_1–C2_1	1.41(4)
C1_1–N2_1	1.43(4)
N2_1–C11_1	1.38(4)
C2_1–C3_1	1.34(4)
N3_1–C33_1	1.39(4)
N3_1–C12_1	1.41(4)
C3_1–C4_1	1.39(5)
C4_1–C5_1	1.44(5)
C4_1–C7_1	1.58(4)
C5_1–C6_1	1.38(4)
C7_1–C9_1	1.51(5)
C7_1–C8_1	1.52(5)
C7_1–C10_1	1.54(5)
C11_1–C16_1	1.36(4)
C11_1–C12_1	1.47(4)
C12_1–C13_1	1.42(4)
C13_1–C14_1	1.40(4)
C14_1–C15_1	1.41(5)
C14_1–C17_1	1.60(4)
C15_1–C16_1	1.38(5)
C17_1–C20_1	1.41(5)
C17_1–C19_1	1.41(5)
C17_1–C18_1	1.72(5)
C21_1–C26_1	1.40(5)
C21_1–C22_1	1.42(5)
C22_1–C23_1	1.44(6)
C22_1–C27_1	1.53(5)
C23_1–C24_1	1.29(6)
C24_1–C25_1	1.35(7)
C25_1–C26_1	1.40(5)
C26_1–C30_1	1.45(5)
C27_1–C28_1	1.50(8)
C27_1–C29_1	1.56(6)
C30_1–C31_1	1.49(5)
C30_1–C32_1	1.57(6)
C33_1–C38_1	1.41(5)
C33_1–C34_1	1.42(5)
C34_1–C35_1	1.39(5)
C34_1–C39_1	1.52(5)
C35_1–C36_1	1.37(6)
C36_1–C37_1	1.37(6)
C37_1–C38_1	1.40(6)
C38_1–C42_1	1.49(6)
C39_1–C40_1	1.54(5)
C39_1–C41_1	1.55(5)
C42_1–C43_1	1.45(7)
C42_1–C44_1	1.59(8)
C45_1–C50_1	1.35(5)
C45_1–C46_1	1.37(5)
C46_1–C47_1	1.38(6)
C47_1–C48_1	1.41(6)
C48_1–C49_1	1.32(6)
C49_1–C50_1	1.35(6)
Bi1_2–N2_2	2.19(3)
Bi1_2–N3_2	2.24(3)
Bi1_2–N1_2	2.29(2)
Bi1_2–Se1_2	2.876(4)
Se1_2–C45_2	1.92(4)
N1_2–C2_2	1.39(4)
N1_2–C21_2	1.48(4)
C1_2–C6_2	1.38(4)
C1_2–N2_2	1.39(4)
C1_2–C2_2	1.44(4)
N2_2–C11_2	1.41(4)
C2_2–C3_2	1.39(4)
N3_2–C12_2	1.42(4)
N3_2–C33_2	1.45(4)
C3_2–C4_2	1.39(4)
C4_2–C5_2	1.39(4)
C4_2–C7_2	1.57(4)
C5_2–C6_2	1.39(4)
C7_2–C8_2	1.51(5)
C7_2–C10_2	1.52(5)
C7_2–C9_2	1.55(5)
C11_2–C16_2	1.34(4)
C11_2–C12_2	1.41(4)
C12_2–C13_2	1.43(4)
C13_2–C14_2	1.37(4)
C14_2–C15_2	1.40(5)
C14_2–C17_2	1.53(5)
C15_2–C16_2	1.41(4)
C17_2–C18_2	1.53(5)
C17_2–C20_2	1.53(5)
C17_2–C19_2	1.56(5)
C21_2–C26_2	1.36(4)
C21_2–C22_2	1.49(5)
C22_2–C23_2	1.41(5)
C22_2–C27_2	1.48(5)
C23_2–C24_2	1.42(5)
C24_2–C25_2	1.26(5)
C25_2–C26_2	1.41(5)
C26_2–C30_2	1.52(5)
C27_2–C28_2	1.47(6)
C27_2–C29_2	1.57(6)
C30_2–C32_2	1.50(6)
C30_2–C31_2	1.52(5)
C33_2–C34_2	1.36(5)
C33_2–C38_2	1.44(5)
C34_2–C35_2	1.35(5)
C34_2–C39_2	1.51(5)
C35_2–C36_2	1.45(6)

C36_2–C37_2	1.37(5)
C37_2–C38_2	1.38(5)
C38_2–C42_2	1.53(5)
C39_2–C41_2	1.46(6)
C39_2–C40_2	1.56(5)
C42_2–C44_2	1.53(5)
C42_2–C43_2	1.54(6)
C45_2–C46_2	1.35(5)
C45_2–C50_2	1.39(5)
C46_2–C47_2	1.42(5)
C47_2–C48_2	1.38(6)
C48_2–C49_2	1.43(6)
C49_2–C50_2	1.37(6)
K1_3–O6_3	2.77(3)
K1_3–O1_3	2.81(2)
K1_3–O3_3	2.83(3)
K1_3–O5_3	2.84(2)
K1_3–O2_3	2.86(2)
K1_3–O4_3	2.87(2)
K1_3–N1_3	2.99(3)
K1_3–N2_3	3.01(3)
O1_3–C2_3	1.40(4)
O1_3–C3_3	1.43(4)
N1_3–C13_3	1.48(5)
N1_3–C1_3	1.48(5)
N1_3–C7_3	1.49(5)
C1_3–C2_3	1.52(5)
O2_3–C4_3	1.41(5)
O2_3–C5_3	1.43(4)
N2_3–C18_3	1.45(5)
N2_3–C12_3	1.47(5)
N2_3–C6_3	1.48(5)
O3_3–C9_3	1.41(4)
O3_3–C8_3	1.43(5)
C3_3–C4_3	1.46(5)
O4_3–C11_3	1.42(4)
O4_3–C10_3	1.44(4)
O5_3–C15_3	1.41(5)
O5_3–C14_3	1.47(5)
C5_3–C6_3	1.48(6)
O6_3–C17_3	1.41(5)
O6_3–C16_3	1.44(4)
C7_3–C8_3	1.47(5)
C9_3–C10_3	1.51(5)
C11_3–C12_3	1.47(5)
C13_3–C14_3	1.52(6)
C15_3–C16_3	1.48(5)
C17_3–C18_3	1.50(6)
K1_4–O3_4	2.78(3)
K1_4–O6_4	2.80(3)
K1_4–O4_4	2.82(3)
K1_4–O2_4	2.84(3)
K1_4–O1_4	2.86(3)
K1_4–O5_4	2.86(2)
K1_4–N2_4	2.99(3)
K1_4–N1_4	3.04(3)
O1_4–C3_4	1.38(5)

O1_4–C2_4	1.38(5)
N1_4–C13_4	1.41(5)
N1_4–C7_4	1.48(5)
N1_4–C1_4	1.51(5)
C1_4–C2_4	1.48(6)
O2_4–C4_4	1.38(5)
O2_4–C5_4	1.41(5)
N2_4–C18_4	1.43(5)
N2_4–C6_4	1.49(5)
N2_4–C12_4	1.50(5)
O3_4–C9_4	1.42(5)
O3_4–C8_4	1.43(5)
C3_4–C4_4	1.52(6)
O4_4–C10_4	1.36(4)
O4_4–C11_4	1.48(5)
O5_4–C15_4	1.41(5)
O5_4–C14_4	1.46(4)
C5_4–C6_4	1.44(6)
O6_4–C17_4	1.37(4)
O6_4–C16_4	1.44(4)
C7_4–C8_4	1.52(6)
C9_4–C10_4	1.53(5)
C11_4–C12_4	1.52(6)
C13_4–C14_4	1.50(6)
C15_4–C16_4	1.52(5)
C17_4–C18_4	1.51(5)

**Atom–Atom–Atom**	**Angle [°]**
N3_1–Bi1_1–N1_1	141.4(9)
N3_1–Bi1_1–N2_1	73.9(9)
N1_1–Bi1_1–N2_1	71.7(9)
N3_1–Bi1_1–Se1_1	104.1(8)
N1_1–Bi1_1–Se1_1	96.3(7)
N2_1–Bi1_1–Se1_1	95.8(7)
C45_1–Se1_1–Bi1_1	106.7(10)
C2_1–N1_1–C21_1	116(2)
C2_1–N1_1–Bi1_1	115.3(18)
C21_1–N1_1–Bi1_1	128(2)
C6_1–C1_1–C2_1	121(3)
C6_1–C1_1–N2_1	124(3)
C2_1–C1_1–N2_1	115(3)
C11_1–N2_1–C1_1	129(3)
C11_1–N2_1–Bi1_1	117.9(18)
C1_1–N2_1–Bi1_1	112.9(19)
C3_1–C2_1–N1_1	128(3)
C3_1–C2_1–C1_1	116(3)
N1_1–C2_1–C1_1	115(2)
C33_1–N3_1–C12_1	118(3)
C33_1–N3_1–Bi1_1	122(2)
C12_1–N3_1–Bi1_1	117.0(19)
C2_1–C3_1–C4_1	124(3)
C3_1–C4_1–C5_1	118(3)
C3_1–C4_1–C7_1	121(3)
C5_1–C4_1–C7_1	120(3)
C6_1–C5_1–C4_1	117(3)
C1_1–C6_1–C5_1	122(3)
C9_1–C7_1–C8_1	110(3)

C9_1–C7_1–C10_1	110(3)
C8_1–C7_1–C10_1	109(3)
C9_1–C7_1–C4_1	113(3)
C8_1–C7_1–C4_1	111(3)
C10_1–C7_1–C4_1	104(3)
C16_1–C11_1–N2_1	125(3)
C16_1–C11_1–C12_1	120(3)
N2_1–C11_1–C12_1	115(2)
N3_1–C12_1–C13_1	128(3)
N3_1–C12_1–C11_1	116(2)
C13_1–C12_1–C11_1	116(3)
C14_1–C13_1–C12_1	123(3)
C13_1–C14_1–C15_1	116(3)
C13_1–C14_1–C17_1	121(3)
C15_1–C14_1–C17_1	121(3)
C16_1–C15_1–C14_1	123(3)
C11_1–C16_1–C15_1	121(3)
C20_1–C17_1–C19_1	118(3)
C20_1–C17_1–C14_1	111(3)
C19_1–C17_1–C14_1	110(3)
C20_1–C17_1–C18_1	106(3)
C19_1–C17_1–C18_1	113(3)
C14_1–C17_1–C18_1	97(2)
C26_1–C21_1–C22_1	117(3)
C26_1–C21_1–N1_1	121(3)
C22_1–C21_1–N1_1	122(3)
C21_1–C22_1–C23_1	119(3)
C21_1–C22_1–C27_1	118(3)
C23_1–C22_1–C27_1	123(4)
C24_1–C23_1–C22_1	119(5)
C23_1–C24_1–C25_1	125(5)
C24_1–C25_1–C26_1	118(4)
C21_1–C26_1–C25_1	121(3)
C21_1–C26_1–C30_1	122(3)
C25_1–C26_1–C30_1	116(3)
C28_1–C27_1–C22_1	111(4)
C28_1–C27_1–C29_1	109(5)
C22_1–C27_1–C29_1	110(3)
C26_1–C30_1–C31_1	117(3)
C26_1–C30_1–C32_1	113(3)
C31_1–C30_1–C32_1	111(3)
N3_1–C33_1–C38_1	119(3)
N3_1–C33_1–C34_1	125(3)
C38_1–C33_1–C34_1	117(3)
C35_1–C34_1–C33_1	121(3)
C35_1–C34_1–C39_1	118(3)
C33_1–C34_1–C39_1	121(3)
C36_1–C35_1–C34_1	122(4)
C35_1–C36_1–C37_1	119(4)
C36_1–C37_1–C38_1	122(4)
C37_1–C38_1–C33_1	120(4)
C37_1–C38_1–C42_1	120(4)
C33_1–C38_1–C42_1	120(4)
C34_1–C39_1–C40_1	113(3)
C34_1–C39_1–C41_1	111(3)
C40_1–C39_1–C41_1	107(3)
C43_1–C42_1–C38_1	114(5)

C43_1–C42_1–C44_1	109(5)
C38_1–C42_1–C44_1	110(4)
C50_1–C45_1–C46_1	115(3)
C50_1–C45_1–Se1_1	125(3)
C46_1–C45_1–Se1_1	119(3)
C45_1–C46_1–C47_1	120(4)
C46_1–C47_1–C48_1	120(5)
C49_1–C48_1–C47_1	119(5)
C48_1–C49_1–C50_1	118(4)
C45_1–C50_1–C49_1	127(4)
N2_2–Bi1_2–N3_2	73.1(10)
N2_2–Bi1_2–N1_2	71.7(9)
N3_2–Bi1_2–N1_2	139.9(9)
N2_2–Bi1_2–Se1_2	94.3(7)
N3_2–Bi1_2–Se1_2	103.7(8)
N1_2–Bi1_2–Se1_2	97.5(5)
C45_2–Se1_2–Bi1_2	105.6(10)
C2_2–N1_2–C21_2	122(2)
C2_2–N1_2–Bi1_2	113.1(18)
C21_2–N1_2–Bi1_2	122.6(17)
C6_2–C1_2–N2_2	127(3)
C6_2–C1_2–C2_2	117(3)
N2_2–C1_2–C2_2	116(3)
C1_2–N2_2–C11_2	126(2)
C1_2–N2_2–Bi1_2	115.1(19)
C11_2–N2_2–Bi1_2	119.1(19)
C3_2–C2_2–N1_2	125(3)
C3_2–C2_2–C1_2	120(3)
N1_2–C2_2–C1_2	115(2)
C12_2–N3_2–C33_2	118(3)
C12_2–N3_2–Bi1_2	116(2)
C33_2–N3_2–Bi1_2	122(2)
C2_2–C3_2–C4_2	121(3)
C3_2–C4_2–C5_2	119(3)
C3_2–C4_2–C7_2	118(3)
C5_2–C4_2–C7_2	122(3)
C6_2–C5_2–C4_2	120(3)
C1_2–C6_2–C5_2	123(3)
C8_2–C7_2–C10_2	108(3)
C8_2–C7_2–C9_2	110(3)
C10_2–C7_2–C9_2	107(3)
C8_2–C7_2–C4_2	109(3)
C10_2–C7_2–C4_2	111(3)
C9_2–C7_2–C4_2	112(3)
C16_2–C11_2–N2_2	126(3)
C16_2–C11_2–C12_2	118(3)
N2_2–C11_2–C12_2	115(2)
C11_2–C12_2–N3_2	116(3)
C11_2–C12_2–C13_2	120(3)
N3_2–C12_2–C13_2	124(3)
C14_2–C13_2–C12_2	119(3)
C13_2–C14_2–C15_2	121(3)
C13_2–C14_2–C17_2	118(3)
C15_2–C14_2–C17_2	122(3)
C14_2–C15_2–C16_2	119(3)
C11_2–C16_2–C15_2	123(3)
C14_2–C17_2–C18_2	110(3)

C14_2–C17_2–C20_2	111(3)
C18_2–C17_2–C20_2	109(3)
C14_2–C17_2–C19_2	108(3)
C18_2–C17_2–C19_2	112(3)
C20_2–C17_2–C19_2	108(3)
C26_2–C21_2–N1_2	122(3)
C26_2–C21_2–C22_2	123(3)
N1_2–C21_2–C22_2	113(3)
C23_2–C22_2–C27_2	125(4)
C23_2–C22_2–C21_2	111(3)
C27_2–C22_2–C21_2	123(3)
C22_2–C23_2–C24_2	122(4)
C25_2–C24_2–C23_2	122(4)
C24_2–C25_2–C26_2	122(4)
C21_2–C26_2–C25_2	119(3)
C21_2–C26_2–C30_2	120(3)
C25_2–C26_2–C30_2	121(3)
C28_2–C27_2–C22_2	114(4)
C28_2–C27_2–C29_2	109(4)
C22_2–C27_2–C29_2	103(3)
C32_2–C30_2–C26_2	108(3)
C32_2–C30_2–C31_2	114(3)
C26_2–C30_2–C31_2	112(3)
C34_2–C33_2–C38_2	119(3)
C34_2–C33_2–N3_2	123(3)
C38_2–C33_2–N3_2	118(3)
C35_2–C34_2–C33_2	121(4)
C35_2–C34_2–C39_2	118(3)
C33_2–C34_2–C39_2	121(3)
C34_2–C35_2–C36_2	121(4)
C37_2–C36_2–C35_2	117(3)
C36_2–C37_2–C38_2	122(4)
C37_2–C38_2–C33_2	119(3)
C37_2–C38_2–C42_2	119(3)
C33_2–C38_2–C42_2	122(3)
C41_2–C39_2–C34_2	115(3)
C41_2–C39_2–C40_2	111(3)
C34_2–C39_2–C40_2	110(3)
C38_2–C42_2–C44_2	111(3)
C38_2–C42_2–C43_2	110(3)
C44_2–C42_2–C43_2	111(3)
C46_2–C45_2–C50_2	119(3)
C46_2–C45_2–Se1_2	121(3)
C50_2–C45_2–Se1_2	121(3)
C45_2–C46_2–C47_2	123(3)
C48_2–C47_2–C46_2	115(4)
C47_2–C48_2–C49_2	123(4)
C50_2–C49_2–C48_2	117(5)
C49_2–C50_2–C45_2	122(4)
O6_3–K1_3–O1_3	131.7(8)
O6_3–K1_3–O3_3	126.4(7)
O1_3–K1_3–O3_3	97.1(7)
O6_3–K1_3–O5_3	59.8(7)
O1_3–K1_3–O5_3	93.7(7)
O3_3–K1_3–O5_3	103.1(7)
O6_3–K1_3–O2_3	92.4(7)
O1_3–K1_3–O2_3	60.9(7)
O3_3–K1_3–O2_3	136.3(7)
O5_3–K1_3–O2_3	114.7(7)
O6_3–K1_3–O4_3	103.5(7)
O1_3–K1_3–O4_3	117.6(7)
O3_3–K1_3–O4_3	59.9(7)
O5_3–K1_3–O4_3	144.8(7)
O2_3–K1_3–O4_3	95.4(7)
O6_3–K1_3–N1_3	120.1(8)
O1_3–K1_3–N1_3	60.6(7)
O3_3–K1_3–N1_3	60.3(7)
O5_3–K1_3–N1_3	61.2(8)
O2_3–K1_3–N1_3	120.7(8)
O4_3–K1_3–N1_3	119.1(8)
O6_3–K1_3–N2_3	61.0(8)
O1_3–K1_3–N2_3	119.3(8)
O3_3–K1_3–N2_3	118.6(9)
O5_3–K1_3–N2_3	120.0(9)
O2_3–K1_3–N2_3	59.4(8)
O4_3–K1_3–N2_3	59.7(9)
N1_3–K1_3–N2_3	178.8(10)
C2_3–O1_3–C3_3	113(3)
C2_3–O1_3–K1_3	118.9(19)
C3_3–O1_3–K1_3	115.3(19)
C13_3–N1_3–C1_3	110(3)
C13_3–N1_3–C7_3	110(3)
C1_3–N1_3–C7_3	109(3)
C13_3–N1_3–K1_3	110(2)
C1_3–N1_3–K1_3	108(2)
C7_3–N1_3–K1_3	110(2)
N1_3–C1_3–C2_3	113(3)
C4_3–O2_3–C5_3	113(3)
C4_3–O2_3–K1_3	112(2)
C5_3–O2_3–K1_3	120(2)
C18_3–N2_3–C12_3	111(3)
C18_3–N2_3–C6_3	111(3)
C12_3–N2_3–C6_3	110(3)
C18_3–N2_3–K1_3	106(2)
C12_3–N2_3–K1_3	110(2)
C6_3–N2_3–K1_3	110(2)
O1_3–C2_3–C1_3	109(3)
C9_3–O3_3–C8_3	111(3)
C9_3–O3_3–K1_3	117(2)
C8_3–O3_3–K1_3	119(2)
O1_3–C3_3–C4_3	110(3)
C11_3–O4_3–C10_3	112(3)
C11_3–O4_3–K1_3	116(2)
C10_3–O4_3–K1_3	111.7(19)
O2_3–C4_3–C3_3	114(3)
C15_3–O5_3–C14_3	110(3)
C15_3–O5_3–K1_3	115(2)
C14_3–O5_3–K1_3	115(2)
O2_3–C5_3–C6_3	108(3)
C17_3–O6_3–C16_3	113(3)
C17_3–O6_3–K1_3	121(2)
C16_3–O6_3–K1_3	120.2(19)
C5_3–C6_3–N2_3	115(4)
C8_3–C7_3–N1_3	113(3)

O3_3–C8_3–C7_3	113(4)
O3_3–C9_3–C10_3	106(3)
O4_3–C10_3–C9_3	111(3)
O4_3–C11_3–C12_3	109(3)
N2_3–C12_3–C11_3	115(3)
N1_3–C13_3–C14_3	114(3)
O5_3–C14_3–C13_3	107(3)
O5_3–C15_3–C16_3	114(3)
O6_3–C16_3–C15_3	109(3)
O6_3–C17_3–C18_3	111(4)
N2_3–C18_3–C17_3	116(3)
O3_4–K1_4–O6_4	133.3(8)
O3_4–K1_4–O4_4	59.6(7)
O6_4–K1_4–O4_4	94.6(7)
O3_4–K1_4–O2_4	125.2(8)
O6_4–K1_4–O2_4	96.6(8)
O4_4–K1_4–O2_4	102.4(8)
O3_4–K1_4–O1_4	102.8(8)
O6_4–K1_4–O1_4	117.1(8)
O4_4–K1_4–O1_4	144.2(8)
O2_4–K1_4–O1_4	60.2(7)
O3_4–K1_4–O5_4	93.7(7)
O6_4–K1_4–O5_4	61.0(7)
O4_4–K1_4–O5_4	115.6(8)
O2_4–K1_4–O5_4	136.2(8)
O1_4–K1_4–O5_4	95.2(7)
O3_4–K1_4–N2_4	120.5(8)
O6_4–K1_4–N2_4	60.5(7)
O4_4–K1_4–N2_4	61.9(8)
O2_4–K1_4–N2_4	59.4(8)
O1_4–K1_4–N2_4	118.4(8)
O5_4–K1_4–N2_4	120.8(8)
O3_4–K1_4–N1_4	60.9(8)
O6_4–K1_4–N1_4	118.7(7)
O4_4–K1_4–N1_4	119.4(8)
O2_4–K1_4–N1_4	120.0(8)
O1_4–K1_4–N1_4	60.8(8)
O5_4–K1_4–N1_4	58.6(7)
N2_4–K1_4–N1_4	178.6(9)
C3_4–O1_4–C2_4	110(3)
C3_4–O1_4–K1_4	115(2)
C2_4–O1_4–K1_4	117(3)
C13_4–N1_4–C7_4	113(3)
C13_4–N1_4–C1_4	110(3)
C7_4–N1_4–C1_4	107(3)
C13_4–N1_4–K1_4	113(2)
C7_4–N1_4–K1_4	106(2)
C1_4–N1_4–K1_4	108(2)
C2_4–C1_4–N1_4	117(3)
C4_4–O2_4–C5_4	113(3)
C4_4–O2_4–K1_4	113(2)
C5_4–O2_4–K1_4	120(2)
C18_4–N2_4–C6_4	113(3)
C18_4–N2_4–C12_4	106(3)
C6_4–N2_4–C12_4	111(3)
C18_4–N2_4–K1_4	107(2)
C6_4–N2_4–K1_4	108(2)
C12_4–N2_4–K1_4	110(2)
O1_4–C2_4–C1_4	112(4)
C9_4–O3_4–C8_4	111(3)
C9_4–O3_4–K1_4	119(2)
C8_4–O3_4–K1_4	120(2)
O1_4–C3_4–C4_4	110(3)
C10_4–O4_4–C11_4	111(3)
C10_4–O4_4–K1_4	115(2)
C11_4–O4_4–K1_4	114(2)
O2_4–C4_4–C3_4	112(4)
C15_4–O5_4–C14_4	114(3)
C15_4–O5_4–K1_4	112(2)
C14_4–O5_4–K1_4	117.3(19)
O2_4–C5_4–C6_4	110(4)
C17_4–O6_4–C16_4	110(3)
C17_4–O6_4–K1_4	119(2)
C16_4–O6_4–K1_4	115(2)
C5_4–C6_4–N2_4	114(4)
N1_4–C7_4–C8_4	112(3)
O3_4–C8_4–C7_4	110(4)
O3_4–C9_4–C10_4	106(3)
O4_4–C10_4–C9_4	113(3)
O4_4–C11_4–C12_4	111(3)
N2_4–C12_4–C11_4	111(3)
N1_4–C13_4–C14_4	112(4)
O5_4–C14_4–C13_4	111(3)
O5_4–C15_4–C16_4	112(3)
O6_4–C16_4–C15_4	107(3)
O6_4–C17_4–C18_4	109(3)
N2_4–C18_4–C17_4	117(4)

Bonds and angles to hydrogen atoms were omitted.

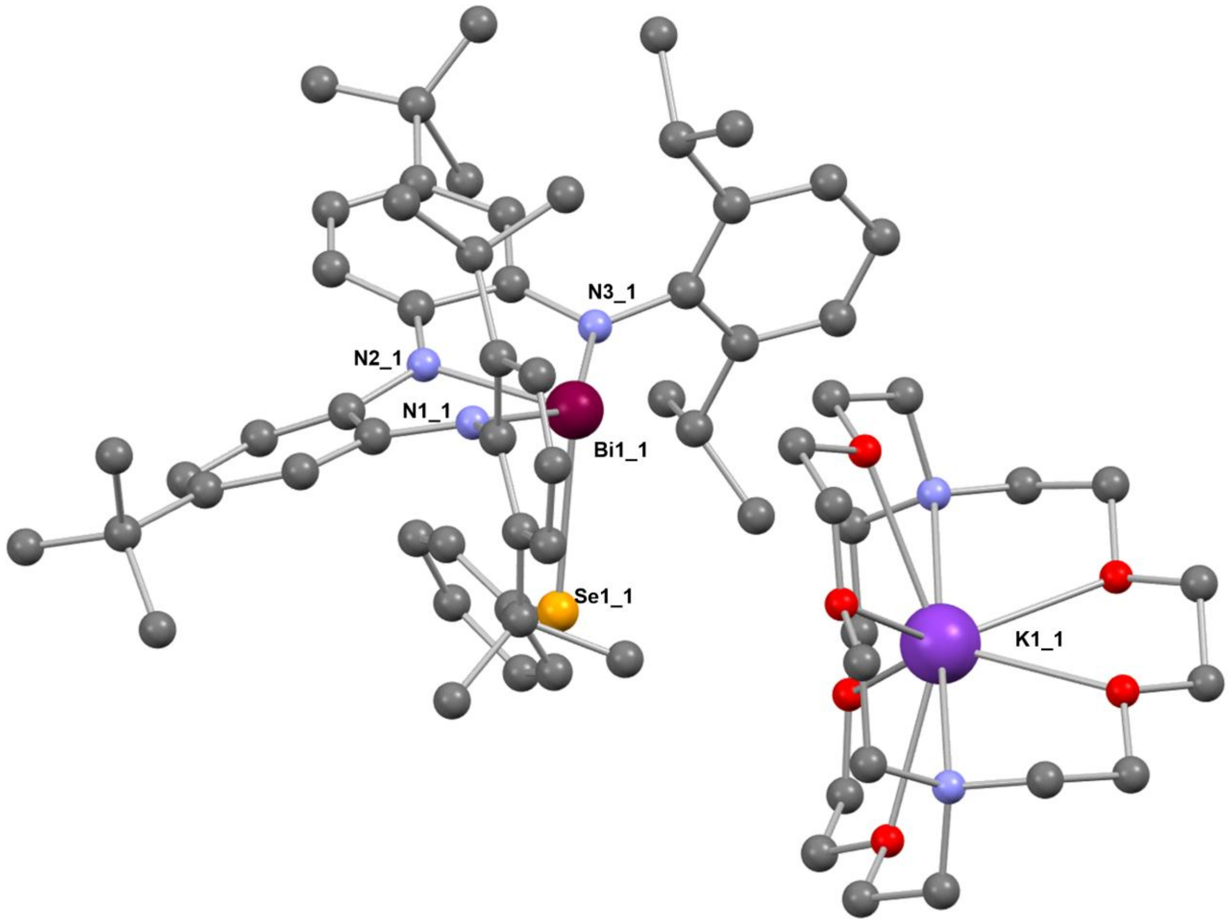

Figure S33. Molecular structure of **5** in the solid state derived by SCXRD, hydrogen atoms, and one compound and cation molecule of the asymmetric unit were omitted for clarity.

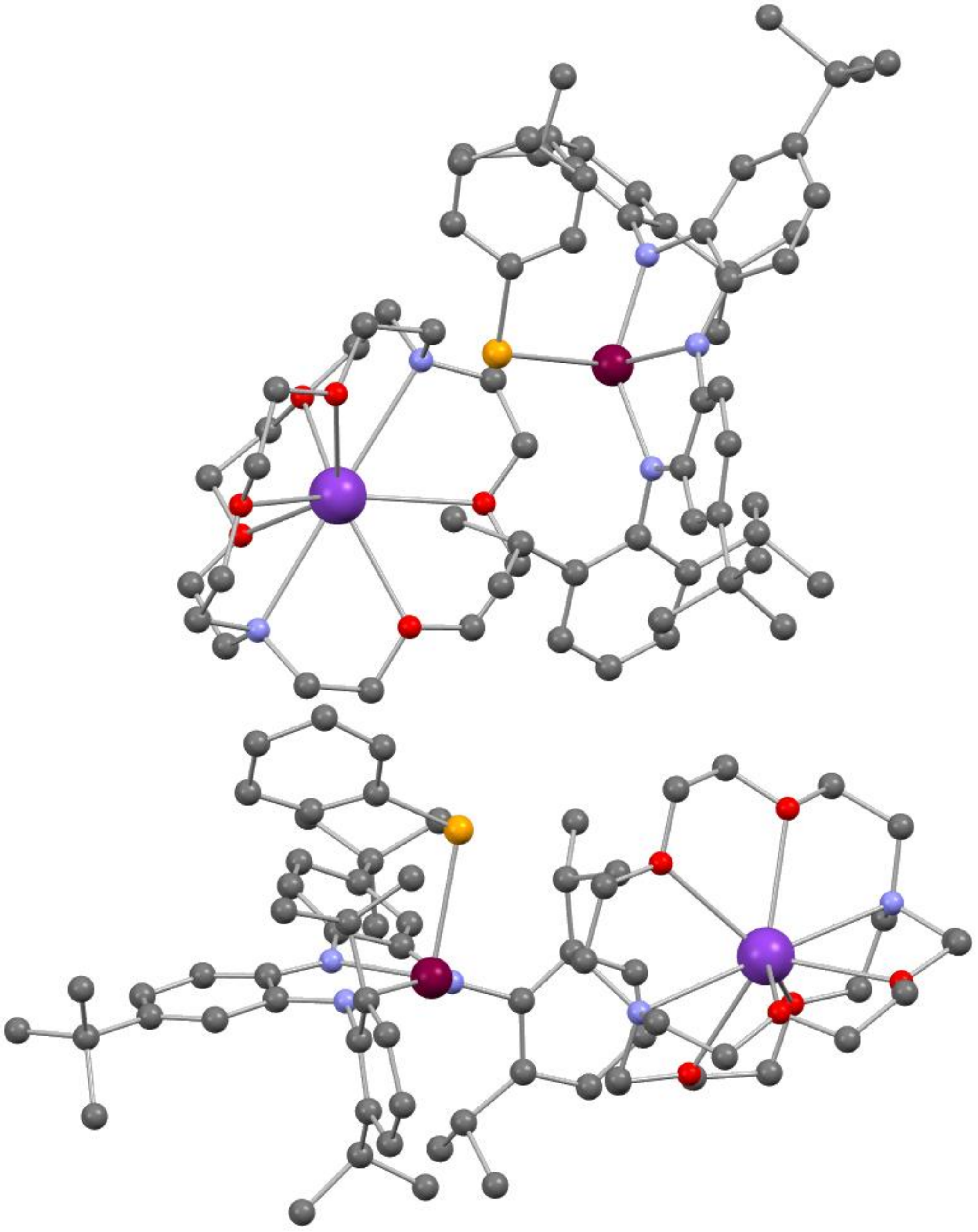

Figure S34. Full content of the asymmetric unit of the dataset showing two molecules of **5** and two [K@222-crypt] cations.

Cyclic voltammetry

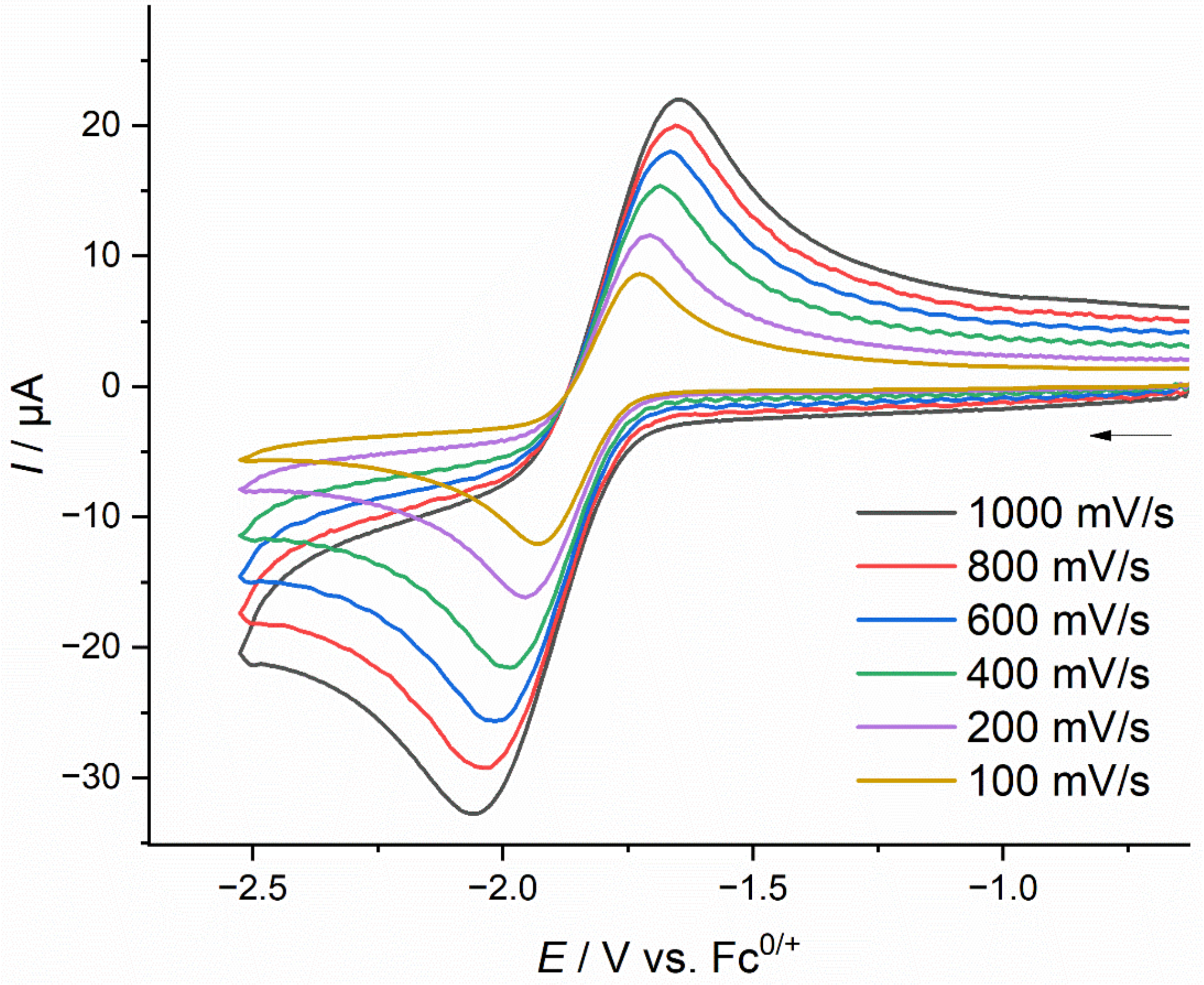

Figure S35. Cyclic voltammogram of **2**, 1 mM, 0.1 M $[NBu_4][PF_6]$, THF, 25 °C.

EPR spectroscopy

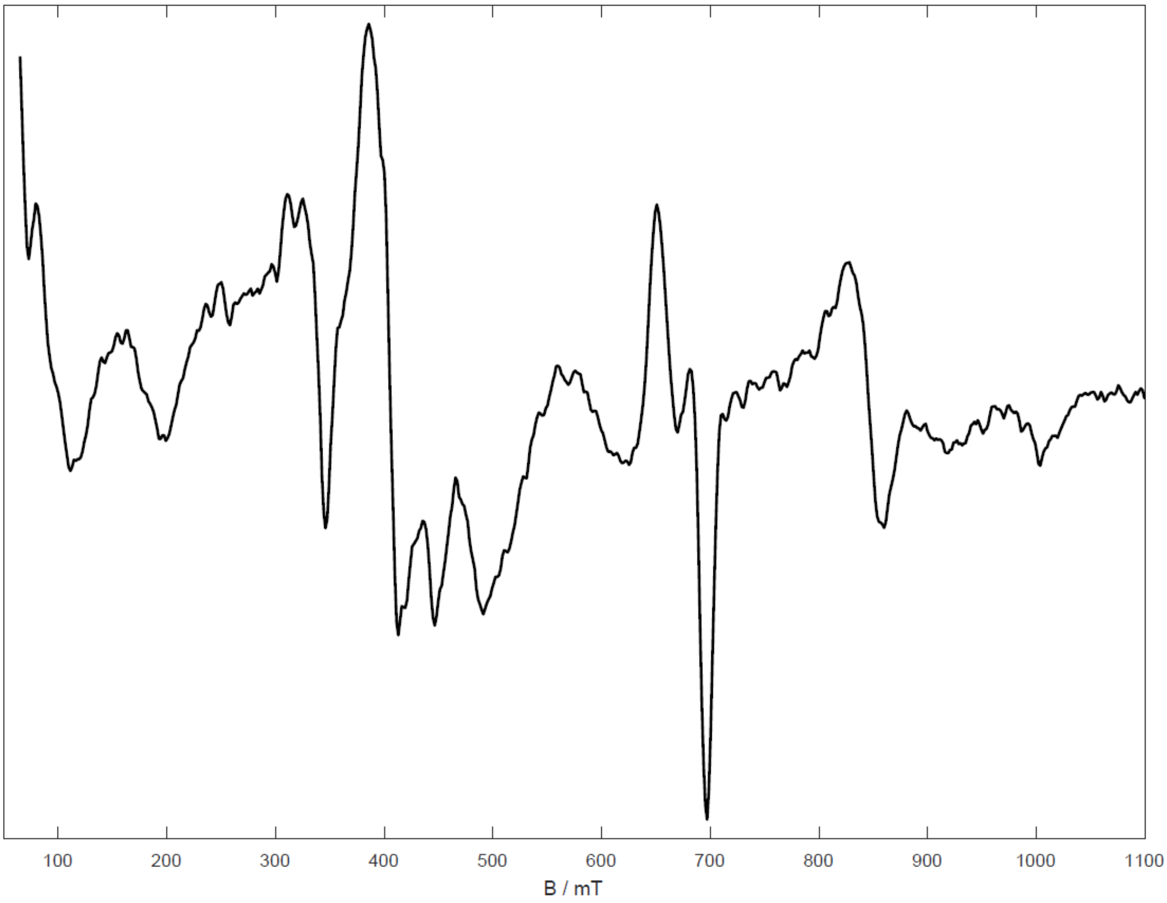

Figure S36. X-Band EPR spectrum of **3**, 2-MeTHF.

The X-band spectrum (9.5 GHz) showed a wide distribution of signals (ranging from 50-1050 mT), but no clear resolution of the hyperfine coupling (hfc) to ^{209}Bi (I = 9/2) could be achieved. This limitation results from the very large Bi hyperfine interaction, as also observed in other isolable Bi(II) compounds.

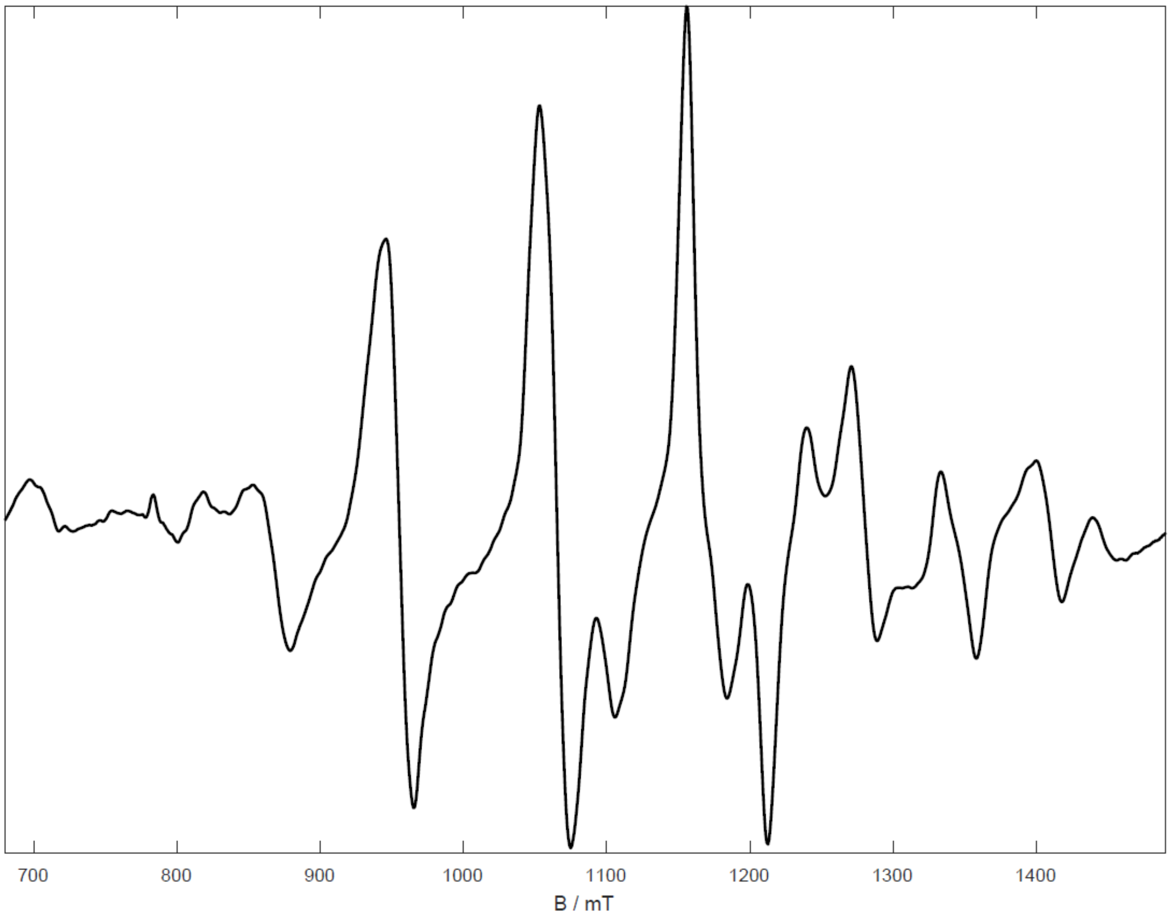

Figure S37. Q-Band EPR spectrum of **3**, 2-MeTHF.

Q-band measurements (34 GHz) revealed a complex pattern of multiple lines; however, due to the limited magnetic field range, the full spectrum could not be resolved.

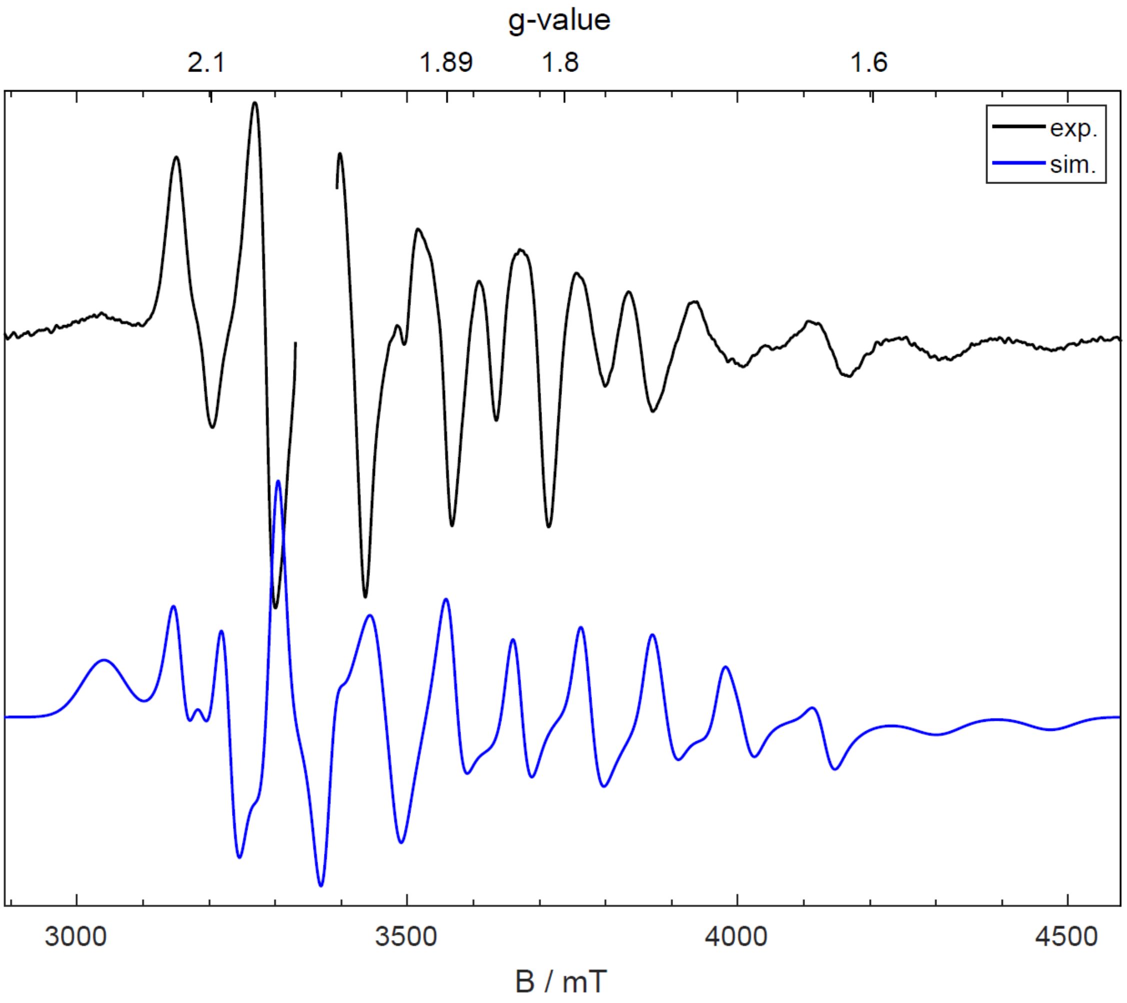

Figure S38. W-Band EPR spectrum of **3**, 2-MeTHF, a sharp signal arising from a paramagnetic manganese containing impurity was cut out from the spectrum (3300–3400 mT).

Closer inspection of the W-band spectrum reveals three resolved lines at the high-field edge, with a splitting of about 140 mT, indicating partial resolution of the g_z component. In contrast, the low-field region remains crowded with overlapping lines and smaller splittings (ca. 110 mT), suggesting that full resolution of the individual g components has not yet been achieved. The combination of a larger hfc component at higher field and two similar and smaller hfc's at lower field indicates axial symmetry of the spectrum. This reflects the planar structure of the compound and the predominantly axial character of the singly occupied molecular orbital (SOMO). The large splittings observed are consistent with strong a hyperfine interaction with ^{209}Bi. With a nuclear spin of *I* = 9/2, ten lines per *g*-component would be expected. However, overlapping due to insufficient *g*-anisotropy combined with the large hyperfine coupling leads to fewer resolved lines. Preliminary simulation attempts based on the W-band spectrum yielded *g*-values of [1.88, 1.83, 1.79] and a ^{209}Bi hyperfine coupling of [2400, 2650, 4000] MHz, underscoring the dominant role of the bismuth center in the electronic structure of the compound.

With an isotropic ^{209}Bi hyperfine coupling of A_{iso}(^{209}Bi) = 3017 MHz the hfc belongs to the larger group of those reported neutral and cationic Bi radicals[19–21] An estimate of the spin population in the 6s, 6p orbitals of the Bi can be made by using tabulated values[22] and using the isotropic and axial part of the hfc. With A_{iso} = 3017 MHz and A_{iso}(^{209}Bi) = 70810 MHz (taking into account for g_{av}=1.83) for a 100% populated 6s orbital, we get a population of ρ_{6s} = 0.04 in the 6s orbital. Accordingly, an estimation with A_{6p}(^{209}Bi) = 608 MHz (scaled for g_{iso} and an angular factor of 2/5) for 100% spin density reveals ρ_{6p}=0.81. It should be noted that the 6p-density is highly sensitive to slight variations in the anisotropic coupling parameters; therefore, this value represents an upper limit. Nevertheless, it clearly demonstrates the dominant Bi(6p) character of the radical, as confirmed by DFT calculations.

SQUID magnetometry

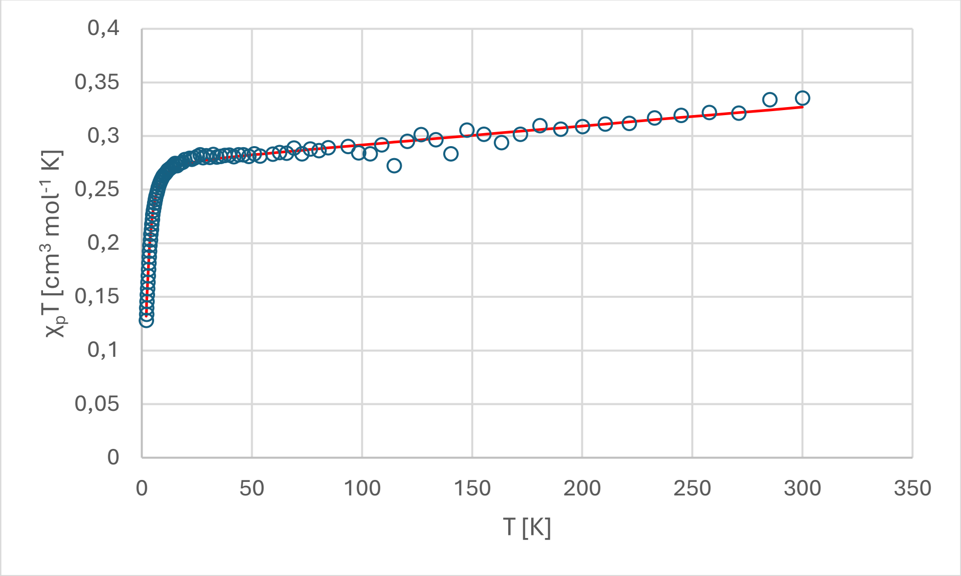

Figure S39. Effective magnetic moment in dependence on the temperature as obtained from the SQUID measurement (blue circles) and as simulated (red line).

Table S9. Experimental magnetic data obtained from the SQUID measurement.[a]

T [K]	Magnetic Field [Oe]	Magnetic Moment [emu]	χ_{mol} [cm³ mol⁻¹]	χ_{p}[b] [cm³ mol⁻¹]	μ_{eff} [μB]
2.000	69999.70	0.03205	0.06318	0.06402	1.0119
2.104	69999.70	0.03181	0.06271	0.06355	1.0340
2.213	69999.70	0.03156	0.06222	0.06306	1.0565
2.328	69999.70	0.03133	0.06176	0.06260	1.0796
2.448	69999.70	0.03103	0.06116	0.06201	1.1019
2.576	69999.70	0.03060	0.06033	0.06117	1.1225
2.710	69999.70	0.03019	0.05951	0.06035	1.1436
2.850	69999.70	0.02974	0.05861	0.05946	1.1642
2.998	69999.70	0.02925	0.05766	0.05850	1.1844
3.153	69999.70	0.02874	0.05666	0.05750	1.2042
3.317	69999.70	0.02817	0.05552	0.05636	1.2228
3.490	69999.70	0.02755	0.05430	0.05514	1.2406
3.671	69999.70	0.02692	0.05306	0.05391	1.2581
3.862	69999.70	0.02623	0.05170	0.05255	1.2739
4.062	69999.70	0.02559	0.05044	0.05128	1.2907
4.274	69999.70	0.02483	0.04894	0.04978	1.3044

4.495	69999.70	0.02412	0.04755	0.04839	1.3189
4.728	69999.70	0.02340	0.04613	0.04697	1.3327
4.974	69999.70	0.02267	0.04468	0.04552	1.3456
5.235	69999.70	0.02190	0.04316	0.04400	1.3573
5.506	69999.70	0.02112	0.04163	0.04248	1.3676
5.790	69999.70	0.02037	0.04014	0.04099	1.3776
6.093	69999.70	0.01960	0.03863	0.03948	1.3869
6.406	69999.70	0.01887	0.03720	0.03804	1.3960
6.738	69999.70	0.01813	0.03573	0.03657	1.4039
7.089	69999.70	0.01739	0.03427	0.03511	1.4109
7.457	69999.70	0.01669	0.03290	0.03375	1.4186
7.843	69999.70	0.01602	0.03159	0.03243	1.4262
8.206	69999.70	0.01545	0.03045	0.03129	1.4330
8.695	69999.70	0.01467	0.02892	0.02976	1.4387
9.133	69999.70	0.01404	0.02768	0.02852	1.4433
9.603	69999.70	0.01344	0.02649	0.02733	1.4488
10.103	69999.70	0.01282	0.02527	0.02612	1.4526
10.627	69999.70	0.01221	0.02406	0.02491	1.4549
11.179	69999.70	0.01166	0.02299	0.02383	1.4597
11.759	69999.70	0.01114	0.02195	0.02279	1.4641
12.370	69999.70	0.01060	0.02090	0.02174	1.4665
13.012	69999.70	0.01010	0.01992	0.02076	1.4699
13.687	69999.70	0.00962	0.01896	0.01980	1.4723
14.398	69999.70	0.00919	0.01811	0.01896	1.4775
15.147	69999.70	0.00876	0.01727	0.01811	1.4811
15.931	69999.70	0.00824	0.01625	0.01709	1.4758
16.759	69999.70	0.00787	0.01551	0.01636	1.4807
17.628	69999.70	0.00747	0.01473	0.01558	1.4819
18.543	69999.70	0.00711	0.01401	0.01485	1.4841
19.506	69999.70	0.00680	0.01341	0.01425	1.4912
21.584	69999.70	0.00613	0.01209	0.01294	1.4943
22.704	69999.70	0.00579	0.01141	0.01226	1.4919
23.883	69999.70	0.00551	0.01085	0.01170	1.4946
25.123	69999.70	0.00524	0.01034	0.01118	1.4989
26.427	69999.70	0.00499	0.00985	0.01069	1.5030

27.799	69999.70	0.00468	0.00922	0.01006	1.4956
29.242	69999.70	0.00446	0.00879	0.00963	1.5006
30.760	69999.70	0.00419	0.00826	0.00910	1.4965
32.357	69999.70	0.00401	0.00790	0.00874	1.5037
34.037	69999.70	0.00375	0.00739	0.00824	1.4973
35.804	69999.70	0.00355	0.00700	0.00785	1.4988
37.659	69999.70	0.00337	0.00664	0.00748	1.5012
39.618	69999.70	0.00318	0.00627	0.00712	1.5017
41.674	69999.70	0.00299	0.00589	0.00673	1.4982
43.837	69999.70	0.00284	0.00560	0.00644	1.5031
46.112	69999.70	0.00268	0.00528	0.00612	1.5028
48.505	69999.70	0.00251	0.00495	0.00579	1.4991
51.022	69999.70	0.00239	0.00471	0.00555	1.5052
53.671	69999.70	0.00223	0.00440	0.00524	1.4997
59.387	69999.70	0.00199	0.00392	0.00477	1.5048
62.469	69999.70	0.00188	0.00371	0.00456	1.5088
65.711	69999.70	0.00176	0.00348	0.00432	1.5068
69.121	69999.70	0.00169	0.00334	0.00418	1.5198
72.705	69999.70	0.00155	0.00305	0.00390	1.5053
76.476	69999.70	0.00148	0.00292	0.00376	1.5174
80.445	69999.70	0.00138	0.00271	0.00356	1.5129
84.621	69999.70	0.00130	0.00257	0.00341	1.5200
93.630	69999.70	0.00114	0.00226	0.00310	1.5232
98.489	69999.70	0.00104	0.00204	0.00288	1.5073
103.604	69999.70	0.00096	0.00189	0.00273	1.5051
108.979	69999.70	0.00093	0.00183	0.00268	1.5271
114.632	69999.70	0.00078	0.00153	0.00237	1.4754
120.585	69999.70	0.00081	0.00160	0.00245	1.5356
126.846	69999.70	0.00078	0.00153	0.00238	1.5525
133.433	69999.70	0.00070	0.00138	0.00222	1.5395
140.372	69999.70	0.00060	0.00117	0.00202	1.5050
147.648	69999.70	0.00062	0.00122	0.00207	1.5626
155.312	69999.70	0.00056	0.00110	0.00194	1.5532
163.374	69999.70	0.00048	0.00095	0.00180	1.5325
171.858	69999.70	0.00046	0.00091	0.00175	1.5526

180.784	69999.70	0.00044	0.00087	0.00171	1.5735
190.169	69999.70	0.00039	0.00077	0.00161	1.5655
200.048	69999.70	0.00036	0.00070	0.00154	1.5715
210.437	69999.70	0.00032	0.00063	0.00148	1.5772
221.361	69999.70	0.00029	0.00057	0.00141	1.5788
232.853	69999.70	0.00026	0.00052	0.00136	1.5919
244.961	69999.70	0.00023	0.00046	0.00130	1.5973
257.670	69999.70	0.00021	0.00041	0.00125	1.6043
271.053	69999.70	0.00017	0.00034	0.00119	1.6031
285.131	69999.70	0.00017	0.00033	0.00117	1.6341
299.968	69999.70	0.00014	0.00027	0.00112	1.6374
2.000	69999.70	0.03205	0.06318	0.06402	1.0119
2.104	69999.70	0.03181	0.06271	0.06355	1.0340
2.213	69999.70	0.03156	0.06222	0.06306	1.0565

[a] m = 9.6 mg [b] χ_p = molar magnetic susceptibility after correction for the diamagnetism of the sample ($\chi_{dia} = -843.05 \cdot 10^{-6}$ cm^3 mol^{-1}, calculated according to literature[6])

Table S10. Fit parameters for the simulation of the SQUID data.

average g factor	g_{avg}	1.710
	error	0.001
temperature-independent paramagnetism [cm^3 mol^{-1}]	TIP	0.000176
	error	0.000004

X-ray absorption spectroscopy

Table S11. Characteristic XAS-energies for **2**, **3** and $BiPh_3$.

Compound	L_1-edge / eV	L_1-whiteline/ eV	L_3-edge/ eV	L_3-whiteline/ eV
**2**	16386.6	16394.9	13417.8	13438.9
**3**	16386.6	16394.9	13419.0	13437.8
**$BiPh_3$**	16388.5	16397.5	13412.0	13442.0

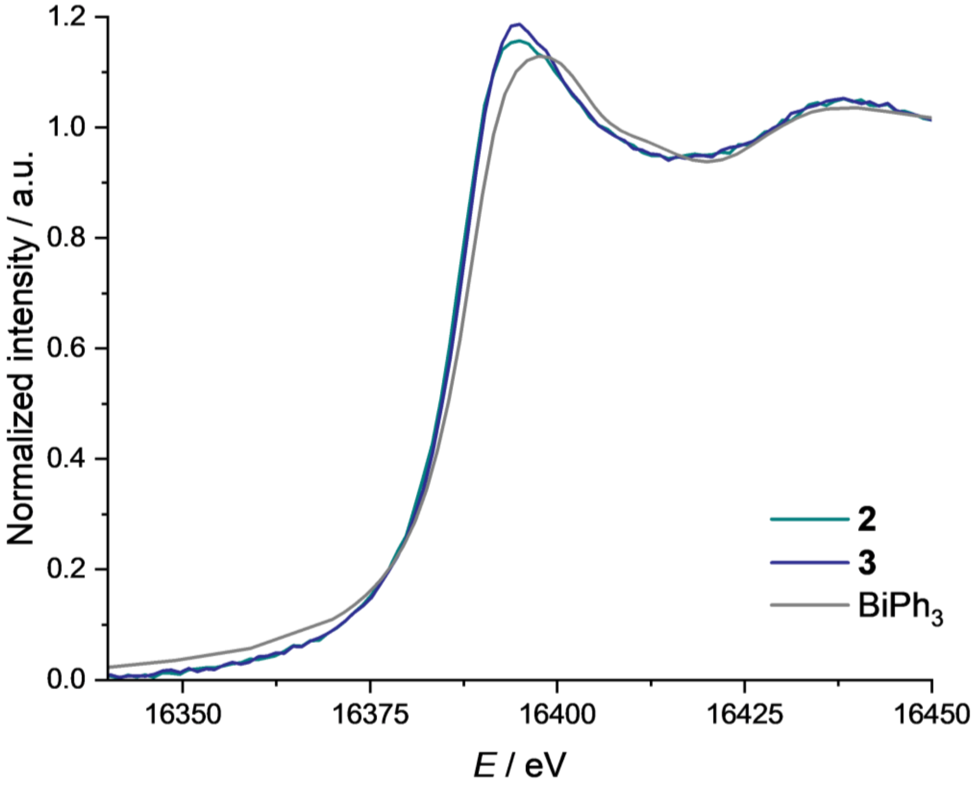

Figure S40. Bi L_1-edge XANES spectra of **2**, **3** and $BiPh_3$.

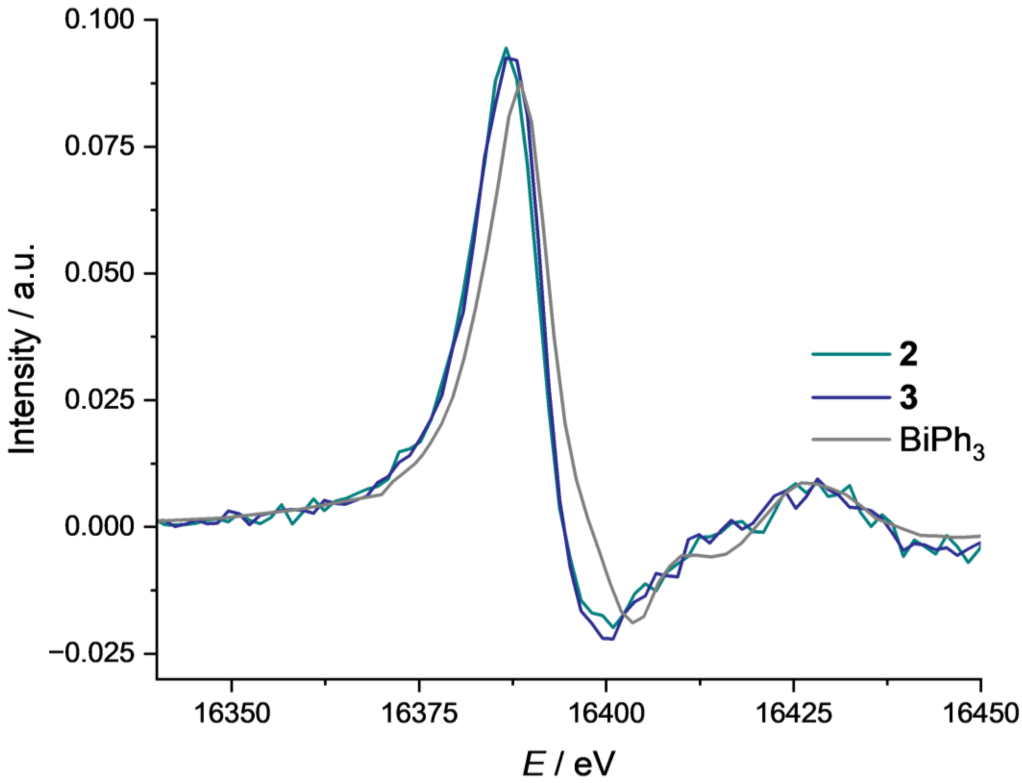

Figure S41. First derivative of the Bi L_1-edge XANES spectra of **2**, **3** and $BiPh_3$.

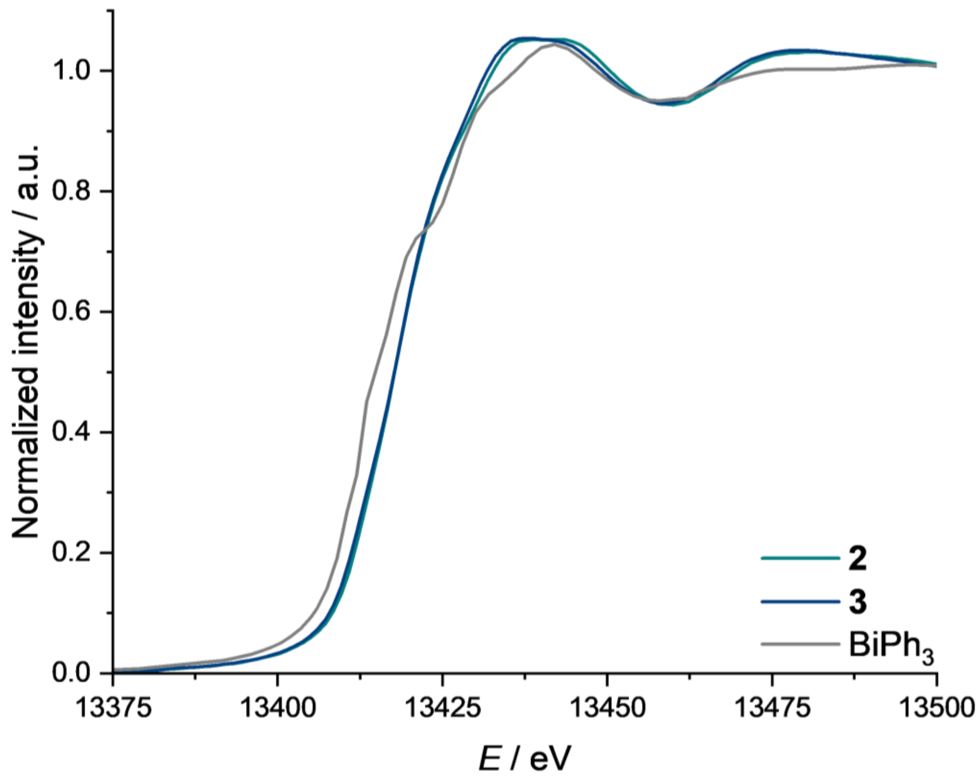

Figure S42. Bi L_3-edge XANES spectra of **2**, **3** and $BiPh_3$.

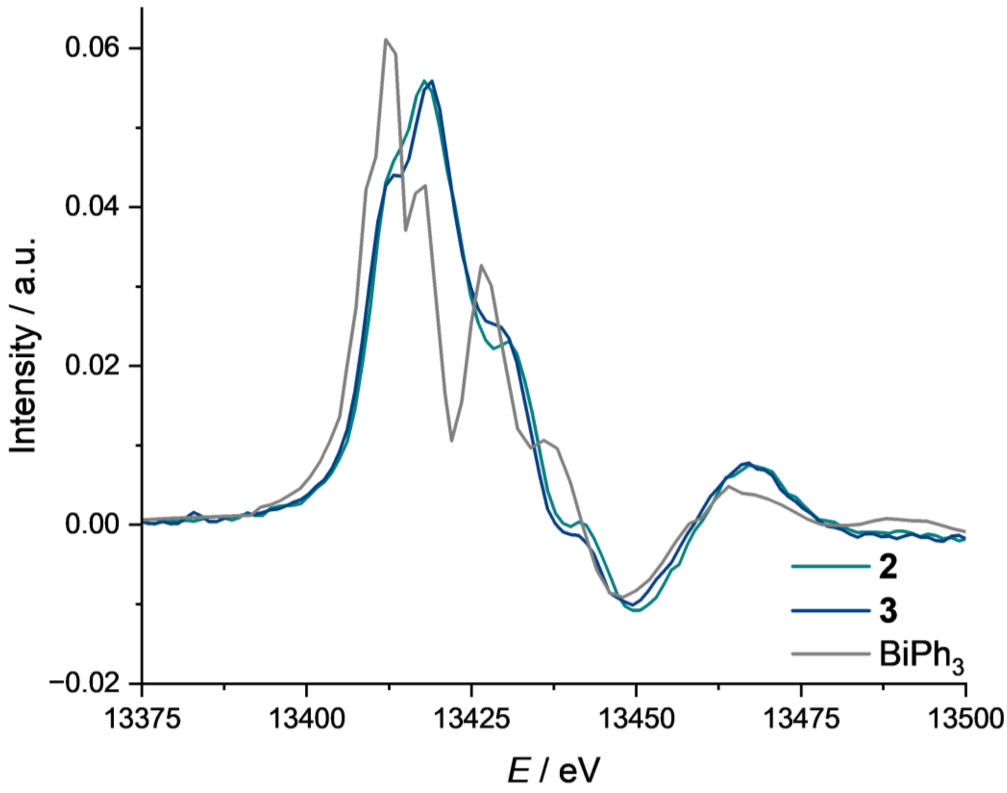

Figure S43. First derivative of the Bi L_3-edge XANES spectra of **2**, **3** and $BiPh_3$.

		Scattering path	Type	N	σ^2 (Å^2)	R_{eff} (Å)	R_{fit} (Å)	delr (Å)
Fit of structure 2 to sample 2								
		Bi...N1.1	Single scattering	2	0.00501	2.2265	2.2550	0.0285
**S_0^2**	1.029	Bi...N3.1	Single scattering	1	0.00501	2.2432	2.2717	0.0285
**E_0**	3.92	Bi...C6.1	Single scattering	4	0.00462	3.0970	3.1118	0.0148
**R-factor**	0.007	Bi...C22.1	Single scattering	2	0.00462	3.1952	3.2100	0.0148
**Red. χ^2**	9.73	Bi...N1.1...C6.1	Obtuse triangle	8	0.00598	3.3447	3.4009	0.0562
		Bi...N2.1...C22.1	Obtuse triangle	4	0.00598	3.4228	3.4790	0.0562
Fit of structure 3 to sample 3								
		Bi...N1.1	Single scattering	1	0.00634	2.2447	2.2532	0.0085
**S_0^2**	1.103	Bi...N2.1	Single scattering	2	0.00634	2.3042	2.3127	0.0085
**E_0**	3.28	Bi...C6.1	Single scattering	4	0.00621	3.1349	3.1476	0.0127
**R-factor**	0.007	Bi...C11.1	Single scattering	2	0.00621	3.2440	3.2567	0.0127
**Red. χ^2**	8.52	Bi...N1.1...C6.1	Obtuse triangle	8	0.00489	3.3883	3.4471	0.0588
		Bi...N3.1...C11.1	Obtuse triangle	4	0.00489	3.4732	3.5320	0.0588

Results from Bi L_3-edge EXAFS fitted parameters, for each scattering path, including degeneracy (**N**), the half path length (D), which in case of single scattering paths it is called interatomic distance (**R_{eff}**), the mean square relative displacement (MSRD), also known as EXAFS Debye-Waller factor (**σ^2**), the amplitude reduction factor **S_0^2**, and **E_0**.

In both cases a Hanning-type window with dk = 2 was chosen to perform the Fourier transformation. FT was performed over the k-space range between 2 to 11.5 Å^{-1} for samples **2** and **3**. The R-range for the fit between model structures (derived by DFT) and samples was between 1.5 and 3.6 Å, for samples **2** and **3**. Upon reduction an elongation of the Bi–N bonds (R_{fit}) is observed due to population of a Bi–NNN π*-antibonding orbital.

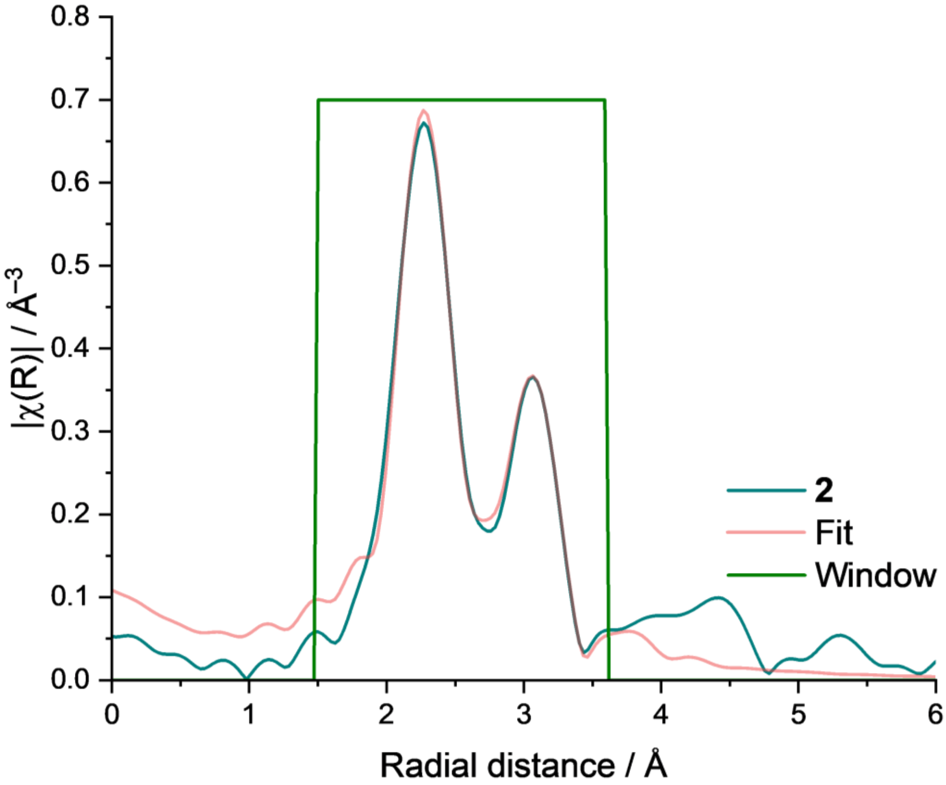

Figure S44. Bi L_3-edge EXAFS spectrum and fit of **2**.

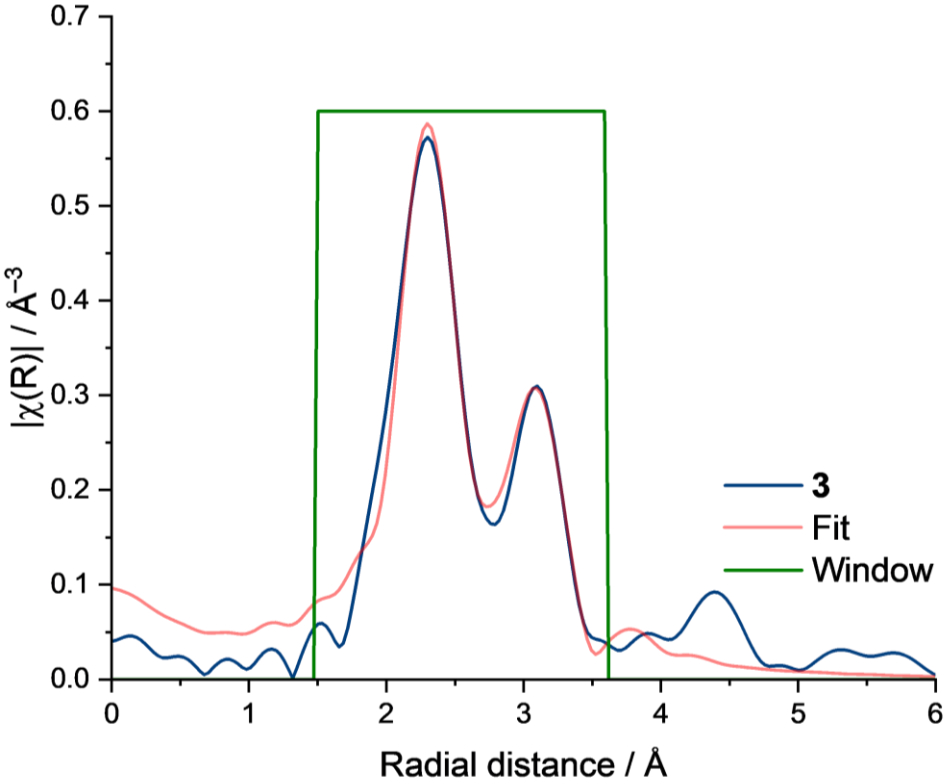

Figure S45. Bi L_3-edge EXAFS spectrum and fit of **3**.

Density functional theory calculations

All DFT calculations were performed with the ORCA 6.0.1 program package. Geometry optimizations were carried out with the PBE0 hybrid functional, using the RIJCOSX approximation and def2-SVP basis set for all atoms except Bi and Se, which were described with def2-TZVP. Solvent effects (tetrahydrofuran) were included via the SMD continuum model. Dispersion interactions were treated with the D3BJ correction. Geometry optimizations were performed with the TightOpt criterion, and vibrational frequency calculations were used to confirm that minimum structures were obtained. Single-point calculations on the optimized geometries were performed at the PBE0/ZORA level with the ZORA-def2-TZVPP basis set for all light atoms and the SARC-ZORA-TZVPP basis set for bismuth and Se, together with SARC/J auxiliary basis sets. Scalar relativistic effects were treated with the ZORA Hamiltonian, including finite nucleus and picture-change corrections. Spin–orbit coupling was included using the SOMF(1X) operator . The *g*-tensor was obtained from the EPRNMR module, with isotropic, dipolar, and orbital contributions analyzed for bismuth. Solvent effects (THF) were included using the SMD model, and basis functions on Bi were decontracted to improve magnetic properties. All calculations employed the SOSCF/KDIIS SCF procedure with tight convergence thresholds and at least DefGrid3 integration grids.[23–39] Molecular orbitals and spin densities were visualized with ChimeraX.[40] Geometries were visualized with Mercury.[41]

The coordinates of all computed structures can be found in the deposited .xyz file.

Frontier molecular orbitals of 2

The computed frontier molecular orbitals of **2** reveal a strong interaction of the empty Bi(6p) orbital stemming from planarization towards a T-shape and the redox-active NNN pincer ligand. While the LUMO can be described as an antibonding combination of the Bi(6p) orbital and the π-manifold of the ligand, the HOMO corresponds to a bonding interaction between the two fragments. The HOMO–1 and HOMO–2 are mainly localized on the redox active ligand.

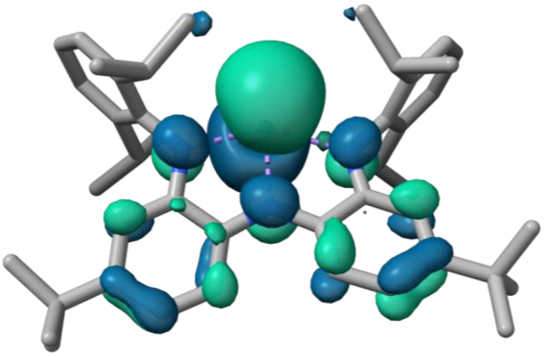

LUMO (−2.29 eV)

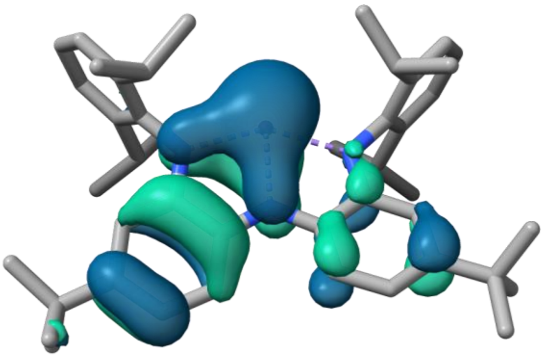

HOMO (−5.35 eV)

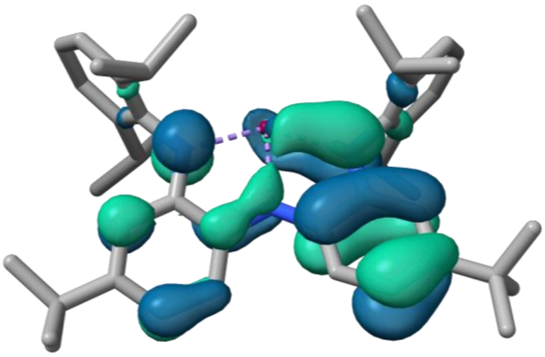

HOMO−1 (−5.43 eV)

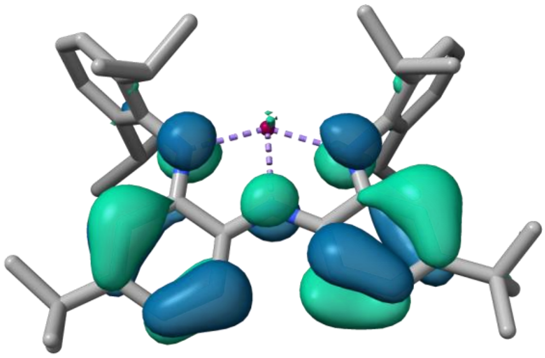

HOMO–2 (−6.26 eV)

Frontier molecular orbitals and spin densities of 3

The calculated frontier molecular orbitals and spin populations of **3** as well as the spin density plot confirm the presence of a Bi(II) radical species. The majority of spin density is located at the Bi center and its adjacent nitrogen atoms due to partial delocalization of electron density between the Bi center and the redox active ligand. The calculated *g*-tensor [1.90, 1.97, 2.00] qualitatively reproduces a low anisotropy.

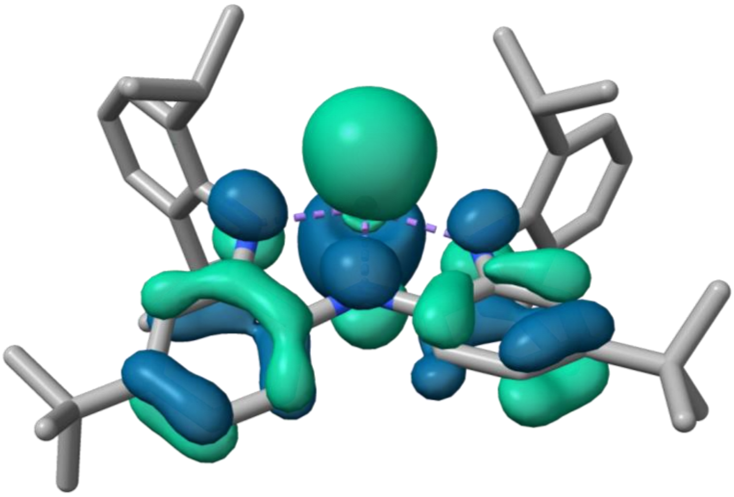

SOMO (−0.32 eV)

Table S12. Calculated spin populations of **3**.

Element	Löwdin spin population	Mulliken spin population
**Bi**	0.60	0.65
**$N_{central}$**	0.09	0.09
**$N_{flanking}$**	0.06	0.06
**Σ**	0.81	0.86

Frontier molecular orbitals of 5

The frontier molecular orbitals of **5** indicate that the extensive coupling between the Bi center and the redox active ligand observed in **2** and **3** is not present in this complex due to occupation of the vacant Bi(6p) orbital by the $PhSe^{-}$ ligand. While the LUMO can be described as a Bi–Se σ*-antibonding orbital the HOMO – HOMO−2 orbitals are ligand based.

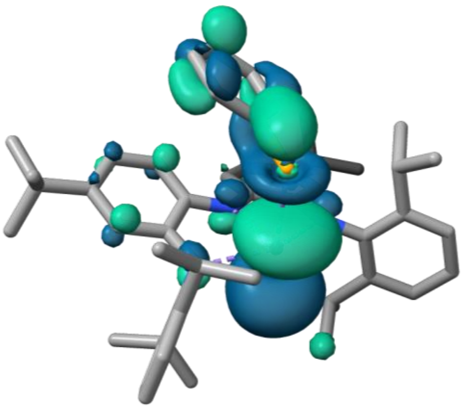

LUMO (−0.60 eV)

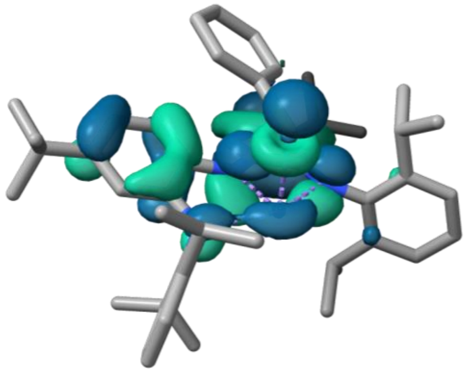

HOMO (−4.09 eV)

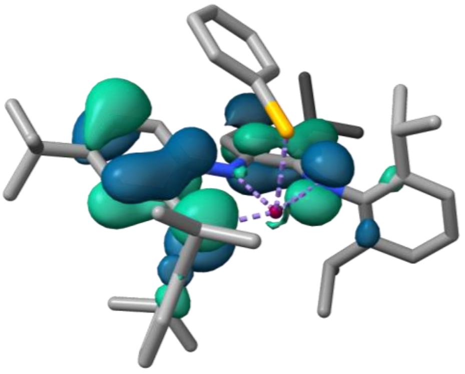

HOMO−1 (−4.74 eV)

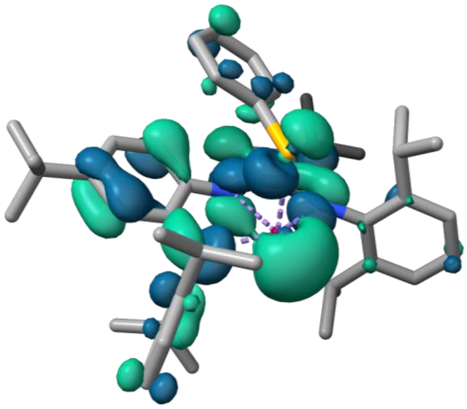

HOMO−2 (–5.30 eV)

Structural comparison of computed structures

Since the utilized redox active ligand can efficiently delocalize electron density towards the Bi center as well as accommodate excess electron density, the ligand's C–C and C–N bonds show distinct alterations that are also observed by SCXRD (see above). Since no high-resolution dataset of **5** could be obtained these changes are compared via the calculated structures of **2**, **3** and **5**.

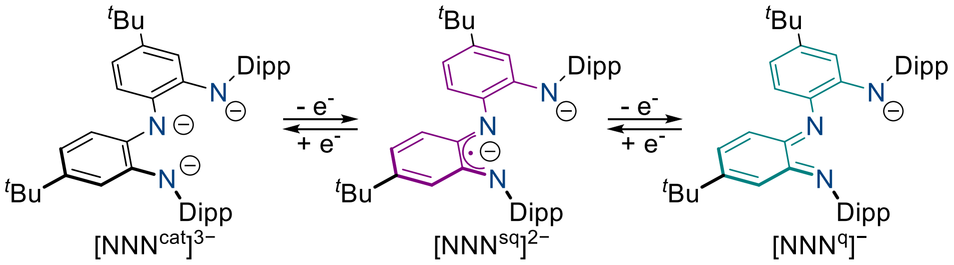

Figure S46. Possible redox states of the NNN pincer ligand.

In **2**, partial quinone character is observed in the calculated structure, in line with the molecular structure in the solid state obtained via SCXRD, indicated by featuring the shortest C–N bonds and most pronounced alterations within the ligand's aromatic C–C bonds. In **3** and **4** a significantly reduced transfer of electron density from the pincer ligand to the Bi center results in longer C–N bonds and more equidistant aromatic C–C bonds.

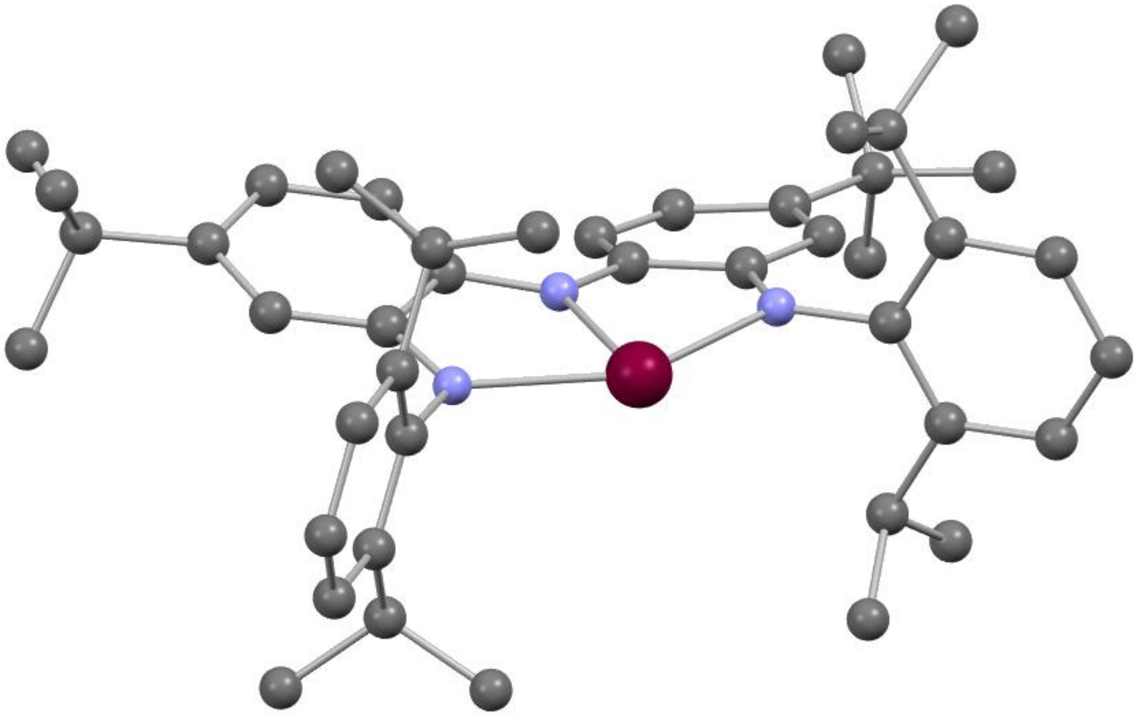

Figure S47. Calculated structure of **2**.

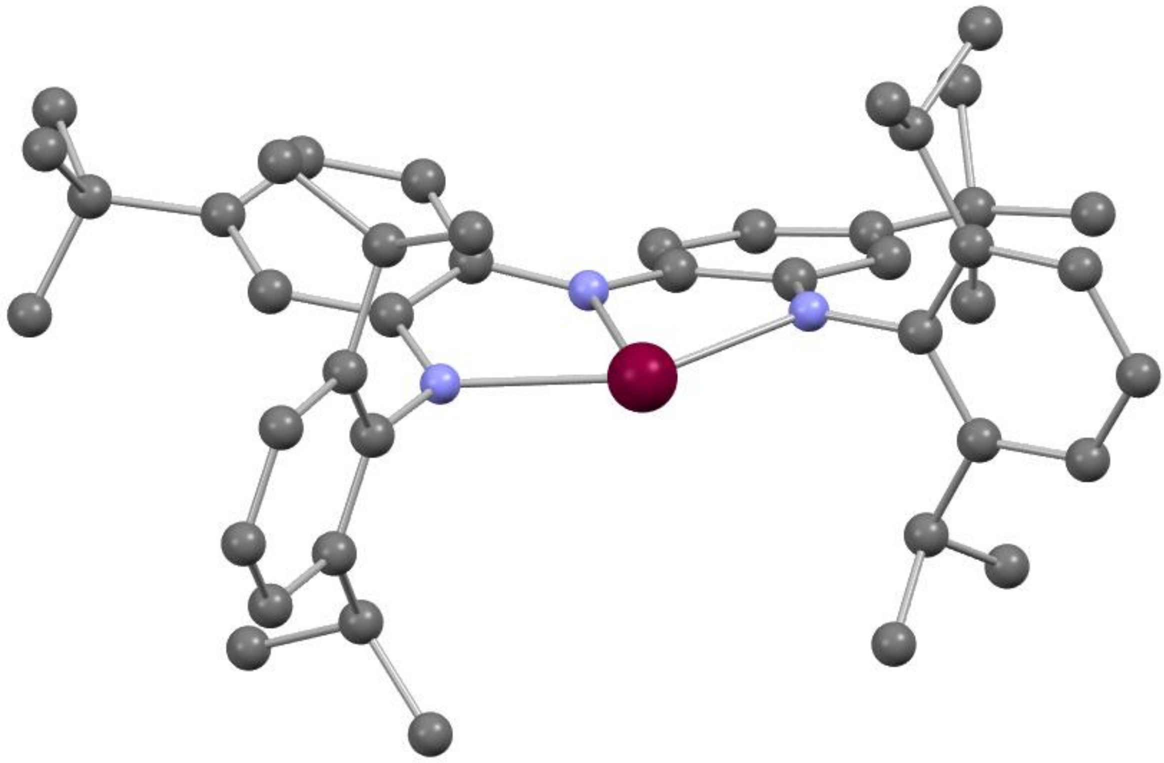

Figure S48. Calculated structure of **3**.

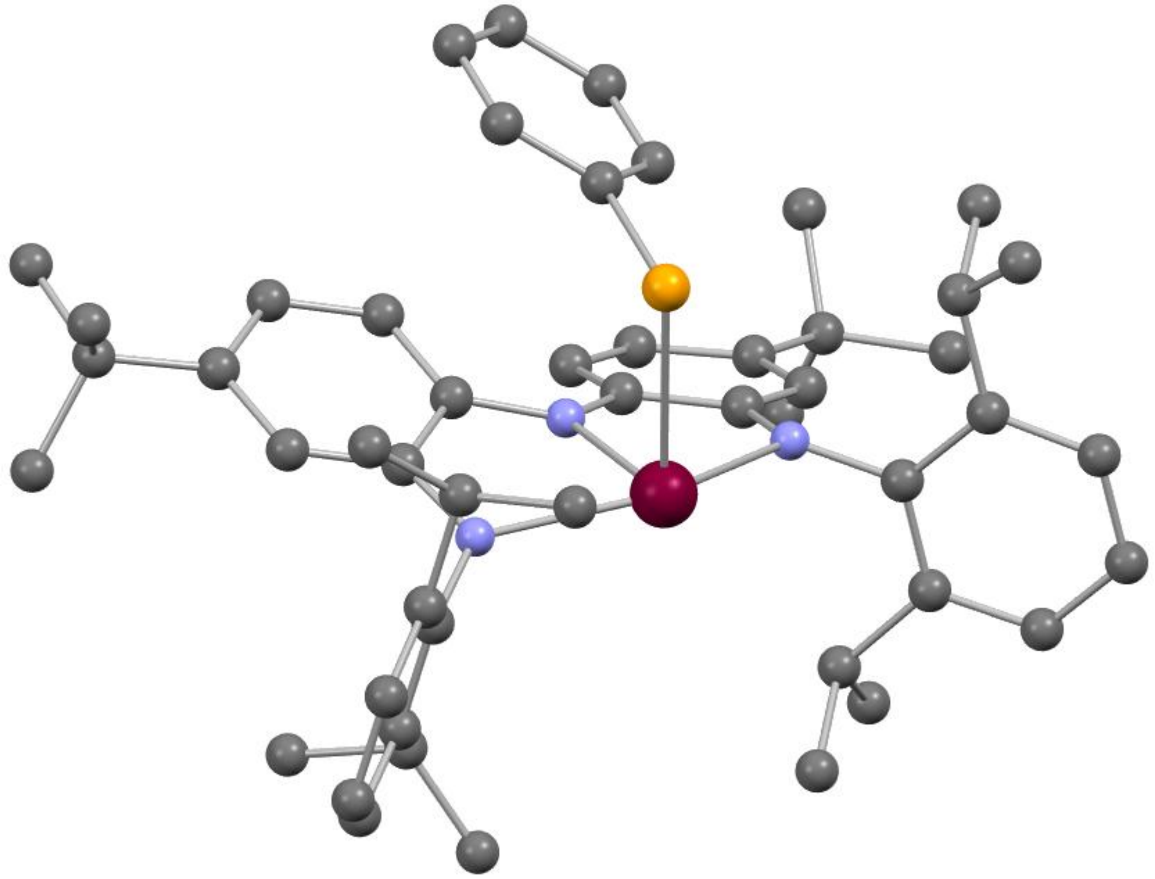

Figure S49. Calculated structure of **5**.

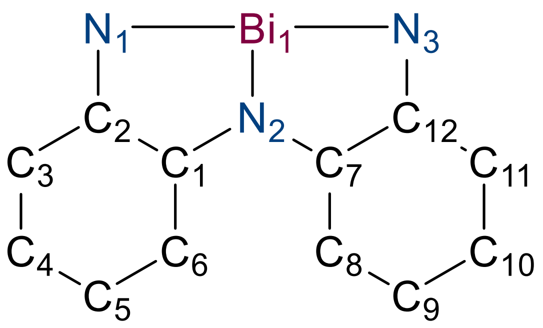

Table S13. Selected calculated bond lengths of **2** - **4**. We benchmarked our DFT methodology to reproduce the bond metrics of **3** as closely as possible.

Bond	2	3	3(EXP)	|3-3(EXP)|	5
**Bi1–N1**	2.243	2.306	2.315(5)	0.009	2.294
**Bi1–N2**	2.212	2.245	2.225(4)	0.020	2.196
**Bi1–N3**	2.241	2.303	2.303(5)	0.000	2.285
**N1–C2**	1.351	1.354	1.367(8)	0.013	1.362
**N2–C1**	1.379	1.381	1.407(6)	0.026	1.385
**N2–C7**	1.380	1.380	1.392(7)	0.012	1.383
**N3–C12**	1.352	1.354	1.363(7)	0.009	1.364
**C1–C2**	1.430	1.436	1.433(8)	0.003	1.433
**C2–C3**	1.413	1.411	1.409(7)	0.002	1.403
**C3–C4**	1.387	1.397	1.383(8)	0.014	1.402
**C4–C5**	1.412	1.402	1.400(8)	0.002	1.398
**C5–C6**	1.385	1.396	1.386(7)	0.010	1.400
**C6–C1**	1.410	1.404	1.397(8)	0.007	1.397
**C7–C8**	1.407	1.402	1.397(8)	0.005	1.402
**C8–C9**	1.389	1.401	1.393(8)	0.008	1.397
**C9–C10**	1.407	1.398	1.384(7)	0.014	1.400
**C10-C11**	1.391	1.402	1.393(8)	0.009	1.399
**C11–C12**	1.409	1.407	1.400(8)	0.002	1.406
**C12–C7**	1.433	1.440	1.429(7)	0.022	1.430

References

[1] A. Bismuto, P. Müller, P. Finkelstein, N. Trapp, G. Jeschke, B. Morandi *J. Am. Chem. Soc.* **2021**, *143*, 10642–10648.

[2] H. Podall, W. E. Foster, A. P. Giraitis *J. Org. Chem.* **1958**, *23*, 82–85.

[3] C. J. Carmalt, N. A. Compton, R. J. Errington, G. A. Fisher, I. Moenander, N. C. Norman, K. H. Whitmire, *Inorg. Synth.* **1996,** *31*, 98–101.

[4] D. L. Dodds, M. D. K. Boele, G. P. F. van Strijdonck, J. G. de Vries, P. W. N. M. van Leeuwen, P. C. J. Kamer *Eur. J. Inorg. Chem.* **2012**, *2012*, 1660–1671.

[5] A. M. Heuer, S. C. Coste, G. Singh, B. Q. Mercado, J. M. Mayer *J. Org. Chem.* **2023**, *88*, 9893–9901.

[6] G. A. Bain, J. F. Berry *J. Chem. Educ.* **2008**, *85*, 532.

[7] N. F. Chilton, R. P. Anderson, L. D. Turner, A. Soncini, K. S. Murray *J. Comput. Chem.* **2013**, *34*, 1164–1175.

[8] A. Guilherme Buzanich, M. Radtke, K. V. Yusenko, T. M. Stawski, A. Kulow, C. T. Cakir, B. Röder, C. Naese, R. Britzke, M. Sintschuk, F. Emmerling *J. Chem. Phys.* **2023**, *158*, 244202.

[9] B. Ravel, M. Newville *J. Synchrotron Radiat.* **2005**, *12*, 537–541.

[10] W. Wang, M. Bao, Y. Dai, X. Liu, C. Liu, C. Liu, Y. Su, X. Wang *Organometallics* **2022**, *41*, 680–685.

[11] K. Michiue, R. F. Jordan *J. Mol. Catal. Chem.* **2008**, *282*, 107–116.

[12] Bruker (2012). Program SAINT. Bruker AXS Inc., Madison, Wisconsin, USA.

[13] L. Krause, R. Herbst-Irmer, G. M. Sheldrick, D. Stalke *J. Appl. Crystallogr.* **2015**, *48*, 3–10.

[14] G. M. Sheldrick *Acta Crystallogr. Sect. Found. Adv.* **2015**, *71*, 3–8.

[15] G. M. Sheldrick *Acta Crystallogr. Sect. C Struct. Chem.* **2015**, *71*, 3–8.

[16] O. V. Dolomanov, L. J. Bourhis, R. J. Gildea, J. a. K. Howard, H. Puschmann *J. Appl. Crystallogr.* **2009**, *42*, 339–341.

[17] C. R. Groom, I. J. Bruno, M. P. Lightfoot, S. C. Ward *Acta Crystallogr. Sect. B Struct. Sci. Cryst. Eng. Mater.* **2016**, *72*, 171–179.

[18] D. Kratzert *Finalcif_V152* **https://dkratzert.de/finalcif.html**.

[19] R. J. Schwamm, J. R. Harmer, M. Lein, C. M. Fitchett, S. Granville, M. P. Coles *Angew. Chem. Int. Ed.* **2015**, *54*, 10630–10633.

[20] X. Yang, E. J. Reijerse, N. Nöthling, D. J. SantaLucia, M. Leutzsch, A. Schnegg, J. Cornella *J. Am. Chem. Soc.* **2023**, *145*, 5618–5623.

[21] J. Haak, J. Krüger, N. V. Abrosimov, C. Helling, S. Schulz, G. E. C. III *Inorg. Chem.* **2022**, DOI 10.1021/acs.inorgchem.2c01141.

[22] J. R. Morton, K. F. Preston *J. Magn. Reson. 1969* **1978**, *30*, 577–582.

[23] F. Neese *Chem. Phys. Lett.* **2000**, *325*, 93–98.

[24] F. Neese *J. Chem. Phys.* **2001**, *115*, 11080–11096.

[25] F. Neese *J. Comp. Chem.* **2003**, *24*, 1740–1747.

[26] F. Neese *J. Chem. Phys.* **2003**, *118*, 3939–3948.

[27] F. Neese *J. Chem. Phys.* **2005**, *122*, Art. No. 034107.

[28] S. Sinnecker, F. Neese *J. Phys. Chem. A* **2006**, *110*, 12267–12275.

[29] F. Neese, F. Wennmohs, A. Hansen, U. Becker *Chem. Phys.* **2009**, *356*, 98–109.

[30] S. Grimme, J. Antony, S. Ehrlich, H. Krieg *J. Chem. Phys.* **2010**, *132*, 154104.

[31] S. Grimme, S. Ehrlich, L. Goerigk *J. Comput. Chem.* **2011**, *32*, 1456–1465.

[32] R. Izsak, F. Neese *J. Chem. Phys.* **2011**, *135*, 144105.

[33] R. Izsak, A. Hansen, F. Neese *Molec. Phys.* **2012**, *110*, 2413–2417.
[34] R. Izsak, F. Neese, W. Klopper *J. Chem. Phys.* **2013**, *139*, DOI 10.1063/1.4819264.
[35] D. Bykov, T. Petrenko, R. Izsak, S. Kossmann, U. Becker, E. Valeev, F. Neese *Molec. Phys.* **2015**, *113*, 1961–1977.
[36] M. Garcia-Rates, F. Neese *J. Comput. Chem.* **2020**, *41*, 922–939.
[37] B. Helmich-Paris, B. de Souza, F. Neese, R. Izsák *J. Chem. Phys.* **2021**, *155*, 104109.
[38] F. Neese *WIRES Comput Molec Sci* **2022**, *12*, e1606.
[39] F. Neese, *J. Comp. Chem.* **2022**, *44*, 381–396.
[40] E. C. Meng, T. D. Goddard, E. F. Pettersen, G. S. Couch, Z. J. Pearson, J. H. Morris, T. E. Ferrin *Protein Sci.* **2023**, *32*, e4792.
[41] C. F. Macrae, I. Sovago, S. J. Cottrell, P. T. A. Galek, P. McCabe, E. Pidcock, M. Platings, G. P. Shields, J. S. Stevens, M. Towler, P. A. Wood *J. Appl. Crystallogr.* **2020**, *53*, 226–235.
